# Establishment of murine gut microbiota in gnotobiotic mice

**DOI:** 10.1016/j.isci.2021.102049

**Published:** 2021-01-12

**Authors:** Jocelyn M. Choo, Geraint B. Rogers

**Affiliations:** 1 SAHMRI Microbiome Research Laboratory, Flinders University College of Medicine and Public Health, Adelaide, SA 5000, Australia; 2 Microbiome and Host Health, South Australia Health and Medical Research Institute, North Terrace, Adelaide, SA 5000, Australia

**Keywords:** Biological Sciences, Microbiology, Microbiome

## Abstract

Determining whether associations between gut microbiota characteristics and host physiology represent causal relationships is a fundamental challenge for microbiome research. We report a detailed investigation of microbiome assembly in C57BL/6 germ-free mice across a period of 70 days and compare the effects of single and multiple rounds of gavage, using both native and antibiotic-disrupted murine donor material. Recipients of the native microbiota did not achieve compositional stability until day 28 and persistent differences to donor microbiota remained until day 70. Performing multiple rounds of gavage significantly increased the cumulative number of detected taxa (mean increase: 10.4%) and compositional similarity to donor, and significantly reduced within-group variance (p < 0.05). Multiple rounds of gavage with antibiotic-disrupted microbiota provided no substantial benefit in relation to compositional similarity to donor or within-group variance. The process of donor microbiota establishment in recipient animals is necessary before experimentation commences and is considerably influenced by donor microbiota characteristics.

## Highlights

• Gut microbiota in germ-free mice stabilize 28 days after native microbiota gavage
• Repeated gavage of native microbiota increases similarity to donor microbiota
• Establishment of antibiotic-disrupted microbiota does not improve with repeated gavage
• Donor microbiota characteristics influence fidelity of microbiota re-establishment

## Graphical abstract

**Figure:**
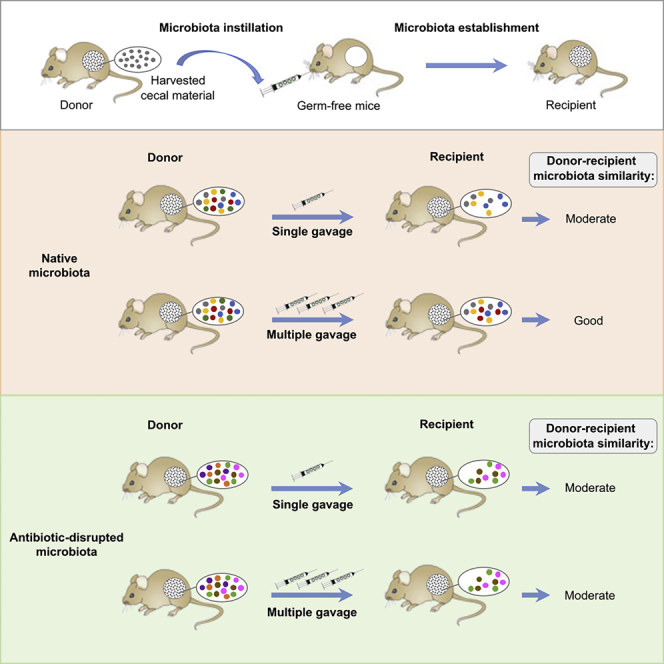

## Introduction

Much of our understanding of the influence of the gut microbiome on human physiology is derived from observational studies. However, where associations between microbiome traits and host phenotype are identified, it then becomes necessary to determine whether these relationships are causal, secondary to observed physiological phenomena, or result from parallel but independent processes.

One of the few experimental strategies available to investigate causality in host-microbiome interactions is to instil intestinal microbiota into germ-free mice and assess whether donor phenotype is recapitulated. Such a strategy is becoming increasingly popular due both to growing access to gnotobiotic facilities and to evidence that antibiotic depletion of intestinal microbiota in conventional mice prior to fecal instillation represents a relatively poor alternative (Ericsson et al., 2017; Le Roy et al., 2018). Additionally, the possibility of using other methods such as co-housing (which is commonly used in murine gut microbiota standardization) (Robertson et al., 2019), for microbiota recapitulation is diminished in most studies due the lag time between the start of an intervention and analysis of gastrointestinal contents to assess for microbiota changes. The approach of transplanting microbiota into germ-free mice has been used, for example, to demonstrate that diet-induced gut microbiota alterations can influence energy expenditure in the context of obesity (Anhe et al., 2018), the role of the gut microbiota in host physiology and immune pathways (Backhed et al., 2004), and to discern immune- and microbiota-associated effects on host susceptibility to colonization resistance (Smith et al., 2019).

Despite the utility of gut microbiota transplantation into germ-free mice as a means to understand microbiome-host relationships, there is little consistency in the manner in which the technique is performed, either for the instillation of intestinal microbiota from animal models (Anhe et al., 2018; Backhed et al., 2004) or human donors (Ridaura et al., 2013; Routy et al., 2018; Gopalakrishnan et al., 2018). In particular, the number of rounds of gavage employed varies between studies, as does the period allowed to elapse between the final gavage and the initiation of the experiment or assessment. Determination of the extent to which donor microbiota are replicated within recipient animals is commonly neglected, and where assessment is performed, it often focuses donor taxon presence/absence, rather than microbiota structure or composition.

Many of the bacterial clades that are closely associated with the regulation of host physiology are obligate anaerobes (Riva et al., 2017; Zeng et al., 2019) and are particularly susceptible to loss of viability during the processing of material for transplant (Papanicolas et al., 2019a, 2019b). Not only can failure to establish such taxa in the gut lumen of gnotobiotic recipient animals lead to divergence in microbiota composition from donor animals but it can allow the proliferation of opportunistic facultative anaerobes (Ubeda et al., 2010; Lupp et al., 2007). Such changes can have profound implications for the metabolic and immuno-regulatory properties of the gut microbiome (Arthur et al., 2012; Galipeau et al., 2015).

A number of previous studies have aimed to describe the process of intestinal microbiota assembly in germ-free mice (Gillilland et al., 2012; El Aidy et al., 2013). In particular, Gillilland and colleagues described microbiota assembly at two mucosal sites, the cecum and the jejunum, during the first 21 days following the instillation of cecal microbiota, reporting evidence of both ecological succession and site-specific effects (Gillilland et al., 2012). Aidy et al. described microbiome compositional dynamics and metabolic function in germ-free mice that received a fecal suspension over a 16-day period (El Aidy et al., 2012, 2013), again with evidence of ecological succession. However, important knowledge gaps remain, including what the effects of multiple rounds of microbiota instillation are and what considerations are necessary when attempting to establish substantially modified donor microbiota.

We investigated the dynamics of donor microbiome assembly in the gut of recipient germ-free mice, including a comparison of the effects of single and multiple rounds of gavage (Figure S1). In addition to the assessment of microbiota transplantation using the native microbiota, an antibiotic-disrupted microbiota was included to represent a modified microbiota that is commonly used to associate the gut microbiota with changes in pathophysiology (Nobel et al., 2015; Sun et al., 2019).

## Results

### Impact of antibiotic exposure on donor microbiota composition

Compared to native donor microbiota, antibiotic-disrupted donor microbiota exhibited lower microbial richness (native = 136 ± 15; antibiotic-disrupted = 78 ± 7), but higher evenness (native = 0.813 ± 0.028; antibiotic-disrupted = 0.85 ± 0.008) and diversity (native = 8.80 ± 0.98; antibiotic-disrupted = 10.34 ± 2.79) (Figures S2A–S2C, respectively). Phylum-level comparisons indicated lower relative abundance of Actinobacteria and Firmicutes and higher relative abundance of Bacteroidetes and Verrucomicrobia in donor microbiota of the antibiotic-exposed mice when compared to microbiota from non-exposed animals (Mann-Whitney test, p < 0.05) (Figure S2D). Bacterial genera including *Lactobacillus, Turicibacter, Faecalibaculum, Bifidobacterium,* and *Enterorhabdus* were predominant in the donor native microbiota but were depleted in the antibiotic-disrupted microbiota (Figure S2E). In contrast, a bloom in *Blautia, Akkermansia* and a taxon in the Muribaculaceae family were observed in the donor antibiotic-disrupted microbiota. Members of the Lachnospiraceae and Ruminococcaceae families were present in both native and antibiotic-disrupted microbiota, although the relative abundance of specific taxa including Lachnospiraceae NK4A136, Lachnospiraceae_UCG-006, *Lachnoclostridium, Eubacterium xylanophilum,* and *Anaerostipes*, differed between these groups.

### Establishment of gut bacterial abundance and microbiota structure

**Figure 1:**
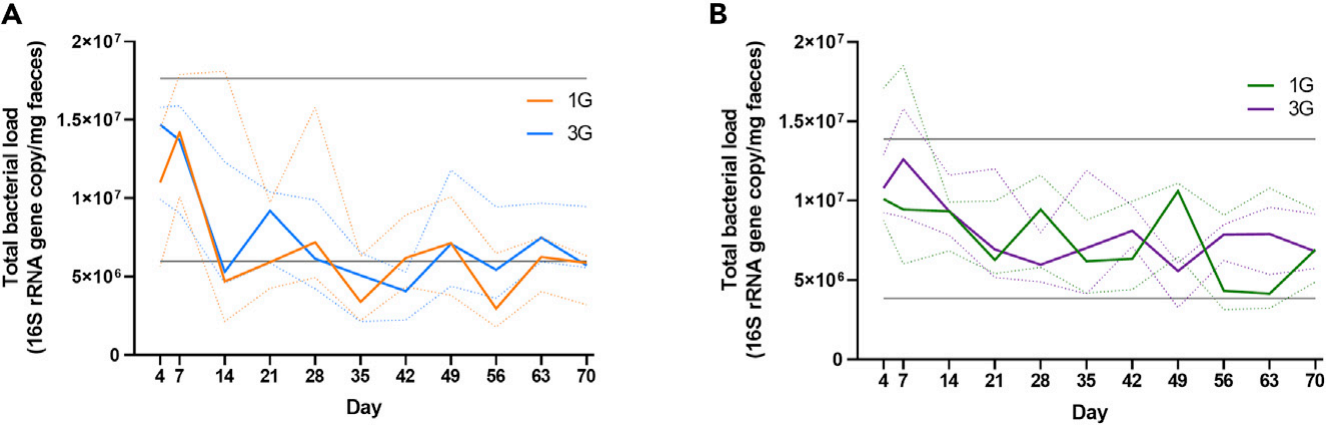
Bacterial load in fecal samples following establishment of gut microbiota in recipient germ-free mice Determination of fecal total bacterial load in recipient mice that received (A) intact native microbiota or (B) microbiota derived from antibiotic-disrupted mice as a single (1G) or three rounds (3G) of gavage. Bacterial load was determined using quantitative PCR of the 16S rRNA gene. The top and bottom gray lines denote the maximum and minimum bacterial load of donor fecal samples. Solid lines and dotted lines denote median values and interquartile ranges, respectively. Statistical comparison between groups were performed using the Mann-Whitney test at a level of p < 0.05 for significance.

In mice that received native donor microbiota, total bacterial load (estimated based on the number of 16S rRNA gene copies) peaked between day four (D4) and day seven (D7) after the initial gavage, before declining to D14 (Figure 1A). From D14 to D70, fecal bacterial load remained stable, averaging 6.6 x 10^6^ (standard deviation (SD): ± 4.3 x10^6^) bacterial cells/mg feces. In mice that received antibiotic-disrupted microbiota, the pattern of an increase in bacterial load following instillation was similar to that seen with native microbiota, with the D14 to D70 average fecal bacterial load at 6.6 x 10^6^ (SD: ± 3.1 x10^6^) bacterial cells/mg feces (Figure 1B). The total bacterial load did not significantly differ between mice that received one or three rounds of gavage of the native or antibiotic-disrupted microbiota.

The evenness and diversity (Faith's phylogenetic diversity) of the microbial community in recipients that received single or multiple gavages of the native microbiota, were higher when compared to the donor inoculum at all timepoints (Figures S3A and S3B). While dynamic changes in alpha diversity measures were observed between recipients that received single or multiple gavages of the native microbiota throughout the 70-day study, microbial diversity was significantly higher for the multiple gavage group at D21 (p = 0.032). In recipients of the antibiotic-disrupted microbiota, the microbial community of recipient mice (single or multiple gavage group) had lower evenness, but higher diversity, in comparison to their donor (Figures S3C and S3D). Significant differences in microbial evenness and diversity between the single and multiple gavage groups were observed particularly during the later timepoints (D63 and D70), as well as at D42 for microbial diversity (p < 0.05).

### Donor taxa representation within recipient fecal microbiota

**Figure 2:**
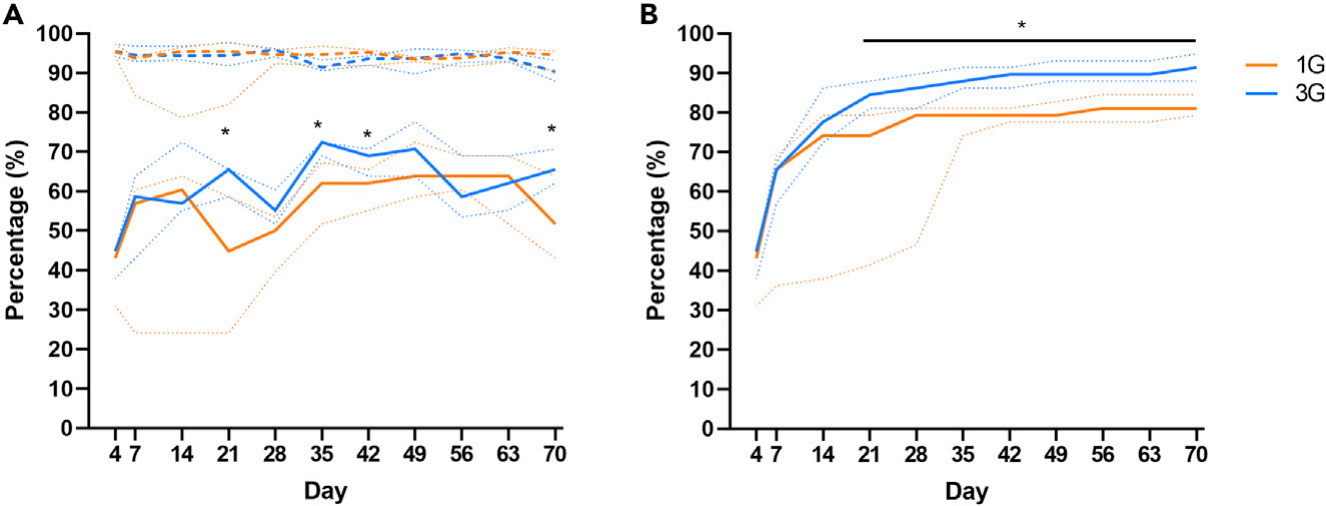
Representation and relative abundance of donor bacterial taxa in mice that received intact native microbiota (A) Percentage of donor taxa represented in recipient germ-free mice that received one (1G) or three (3G) rounds or fecal gavage of the native microbiota (solid line). The total relative abundance of donor taxa in the recipient mice at each time points were determined (dashed line). (B) The cumulative detection of donor bacterial taxa in recipient mice were determined. Solid or dashed lines denote median values, while the dotted lines denote interquartile ranges. Statistical comparison between groups were performed using the Mann-Whitney test at a level of p < 0.05 for significance.

**Figure 3:**
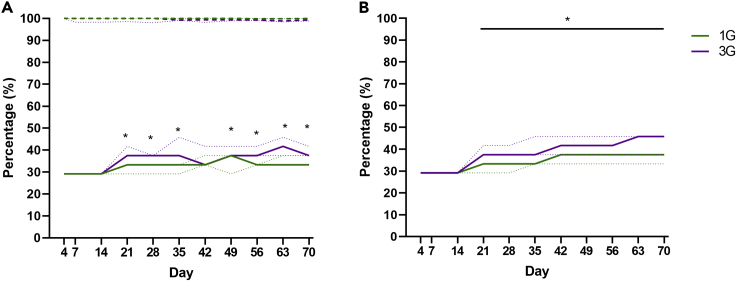
Representation and relative abundance of donor bacterial taxa and in mice receiving material from antibiotic-disrupted mice (A) Percentage of donor taxa represented in recipient germ-free mice that received one (1G) or three (3G) rounds or fecal gavage of the antibiotic-disrupted microbiota (solid line). The total relative abundance of donor taxa in the recipient mice at each time points were determined (dashed line). (B) The cumulative detection of donor bacterial taxa in recipient mice were determined. Solid or dashed lines denote median values, while the dotted lines denote interquartile ranges. Statistical comparison between groups were performed using the Mann-Whitney test at a level of p < 0.05 for significance.

Initial assessment of donor-recipient microbiota similarity was based on the percentage of donor taxa that were detectable in recipient feces. Similar trends were observed for recipients of one and three rounds of gavage, with detected taxa rising steeply from D4 (median, interquartile range (IQR): 1G = 43.1%, 32.8–44.0; and 3G = 44.8%, 40.5–44.8) to D14 (median, IQR: 1G = 60.3%, 39.7–62.9; and 3G = 56.9%, 56.0–69.0) (Figure 2A). At D21, the percentage of donor taxa detected was significantly higher in mice that received three rounds of gavage, compared to those that received one (Mann-Whitney test, p < 0.05). This trend remained at all timepoints bar one (D56), and was significant at D35, D42, and at the end of the study period (D70) (median, IQR: 1G = 51.7%, 46.6–60.4; 3G = 65.5%, 62.9–69.0; p < 0.05). In recipients of antibiotic-disrupted microbiota, detected donor taxa were significantly higher in mice that received multiple gavages. The difference between recipients of single and multiple rounds of gavage remained significant throughout the 70-day study (median, IQR: 1G = 33.3%, 33.3–37.5; 3G = 37.5%, 37.5–39.6; p < 0.05), except at D42 (Figure 3A). In recipients of the native and antibiotic-disrupted microbiota, taxa that were of donor origin represented at least 89.9% and 95.5% of the bacterial relative abundances in recipients across D4 to D70, respectively (Figures 2A and 3A, dotted lines). These results confirmed that majority of the taxa in recipients were transferred from the donors, as expected. The remaining taxa were found to be of low relative abundance in recipients (mean relative abundance <0.1%), and therefore may not have been detected in the donor material.

The percentage of donor taxa detectable in recipient animals fluctuated over the 70-day period of assessment (Figure 2A). As mice were housed in a controlled environment in which the only route of bacterial acquisition was fecal gavage, intermittent detection of individual taxa was likely to be due to sampling bias. Assessment of the relative abundance of taxa in relation to their presence or intermittent absence at each time point supported this hypothesis (Figure S4), with the latter taxa relative abundance being significantly closer to the threshold of detection (median, IQR: presence = 0.005, 0.001–0.013; absence = 0, 0 - 0.001; Mann-Whitney test, p < 0.05). We therefore also assessed the percentage of donor taxa detected as a cumulative measure. For the native microbiota, median percentage of donor taxa detected increased rapidly from D4 to D14 in recipients of both multiple gavage and single gavage groups (Figure 2B). From D21, the percentage donor taxa detected was significantly higher in recipients of multiple gavages compared to single gavage at all timepoints until D70 (median difference at D70 = 10.4%; Mann-Whitney test, p < 0.05). In recipients of the antibiotic-disrupted microbiota, the median percentage of donor taxa remained similar from D4 to D14 for both single or multiple gavage groups (Figure 3B). Multiple gavage of the antibiotic-disrupted microbiota was also associated with a detection of significantly higher median percentage of donor taxa compared to single gavage from D21 until the end of the 70-day study (median difference at D70 = 8.3%; Mann-Whitney test, p < 0.05). At the end of the 70-day the study, the cumulative representation of donor taxa in recipient mice that received multiple gavages of the antibiotic-disrupted microbiota were lower (Median: 45.8% [IQR 37.5–45.8]) compared to those that received the native microbiota (Median: 91.4% [IQR 88.8–94.0]).

### Establishment of donor microbiota composition

**Figure 4:**
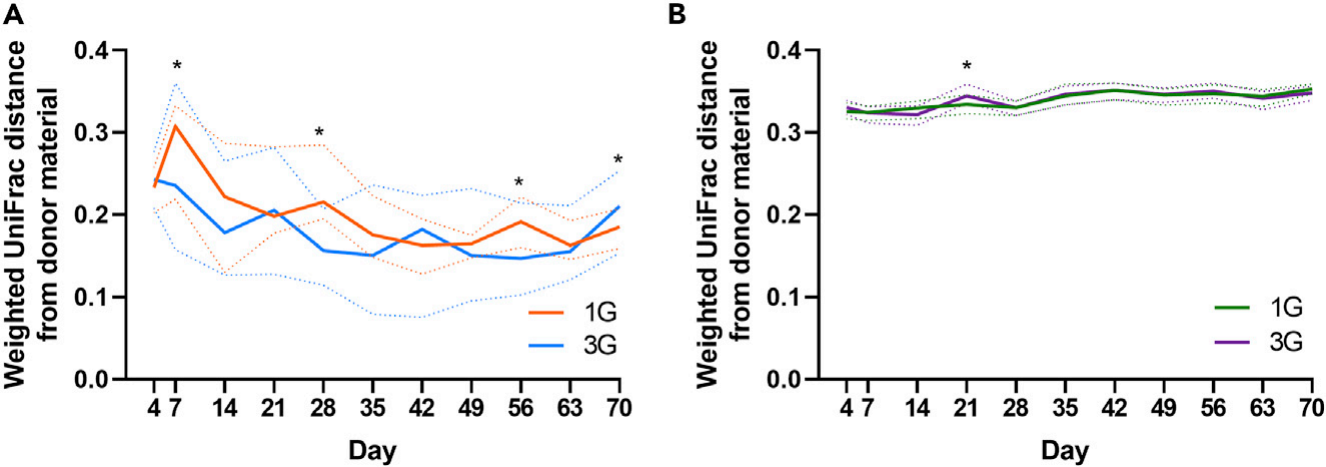
Compositional similarity of the donor microbiota and recipient microbiota Weighted UniFrac distance between the microbiota of recipient mice that received (A) native microbiota or (B) microbiota derived from antibiotic-disrupted mice, and their respective donor microbiota throughout the 70-day study period. Recipient mice received either one round (1G) or three rounds (3G) of donor microbiota. The solid and dotted lines denote the median and interquartile ranges, respectively. Statistical comparison between the 1G and 3G groups at each time point was performed using the Mann-Whitney test (p < 0.05).

**Figure 5:**
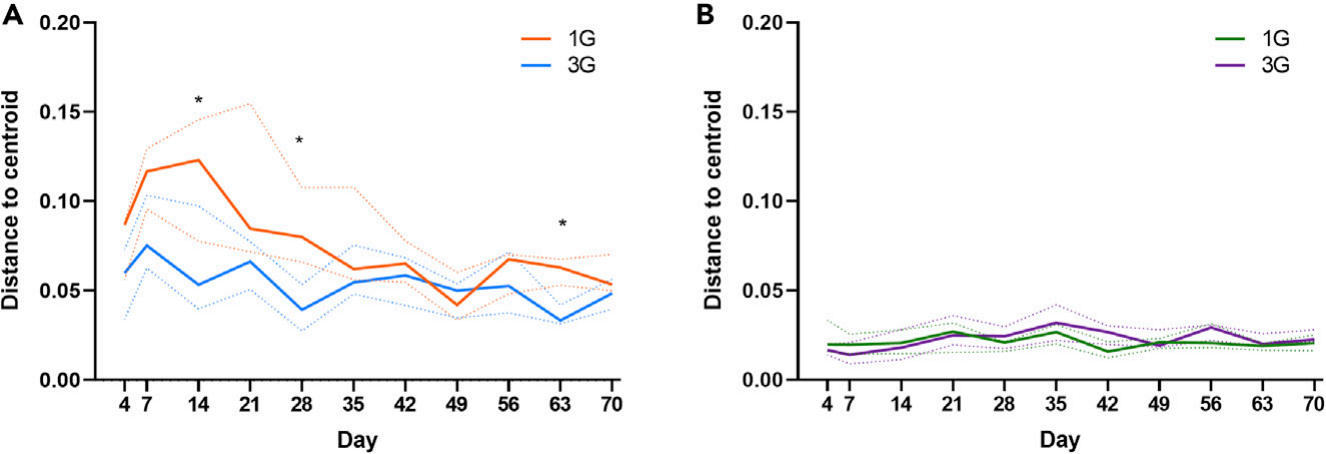
Distance to group centroid of the recipient microbiota Compositional variance of microbiota among recipient mice that received (A) native microbiota or (B) microbiota derived from antibiotic-disrupted mice were determined based on distances to their respective group centroid at each time point throughout the 70-day study period. Recipient mice received either one round (1G) or three rounds (3G) of donor microbiota. The solid and dotted lines denote the median and interquartile ranges, respectively. Statistical comparison between the 1G and 3G groups at each time point was performed using the Mann-Whitney test (p < 0.05).

Recipient-donor microbiota similarity was assessed based on weighted UniFrac distance (Figure S5). Broadly, compositional distance of the native microbiota recipient to the donor decreased (increased similarity) in the multiple gavage and single gavage groups to D35 (Figure 4A). Recipients of a single gavage of the native microbiota had lower similarity to donor (higher weighted UniFrac distance) compared to recipients of multiple gavages (median [IQR]: 0.20 [0.16–0.23] vs 0.18 [0.15–0.22], respectively), with the exception at D42 and D70 during the study (Figure 4A). Differences between single and multiple gavage groups achieved significance at D7, D28, D56 and D70 (Mann-Whitney test, p < 0.05). Microbiota composition between consecutive timepoints also differed significantly only for the multiple gavage group at D28 to D35 (PERMANOVA p = 0.021), after which, no further significant variation was observed (PERMANOVA p < 0.05). Recipients of the antibiotic-disrupted microbiota showed substantially less variation in similarity when compared to those observed for recipients of native microbiota (Figure 4B). Donor-recipient microbiota similarity did not differ significantly between single and multiple gavage groups that received antibiotic-disrupted microbiota (median interquartile range [IQR]: 0.34 [0.33–0.35] for both groups), except at D21 (Mann-Whitney test, p < 0.05). Microbiota composition between consecutive time points in these animals did not differ significantly, except for D63 to D70 in recipients of single gavage (PERMANOVA p > 0.05). No compositional variance were detected between the cages within each group at all time points. Inter-individual microbiota variation between group members was assessed based on distance to group centroid. In recipients of native microbiota, distance to group centroid peaked at D7, and declining thereafter (Figure 5A). Distance to centroid was higher at all but one time point (D49) in mice that received one round of fecal gavage compared to those that received three, achieving statistical significance at D14, D28, and D63 (Mann-Whitney test, p < 0.05). Distance to group centroid in recipients of antibiotic-disrupted microbiota did not differ significantly between single and multiple gavage groups (Figure 5B).

### Temporal dynamics in taxon relative abundance

**Figure 6:**
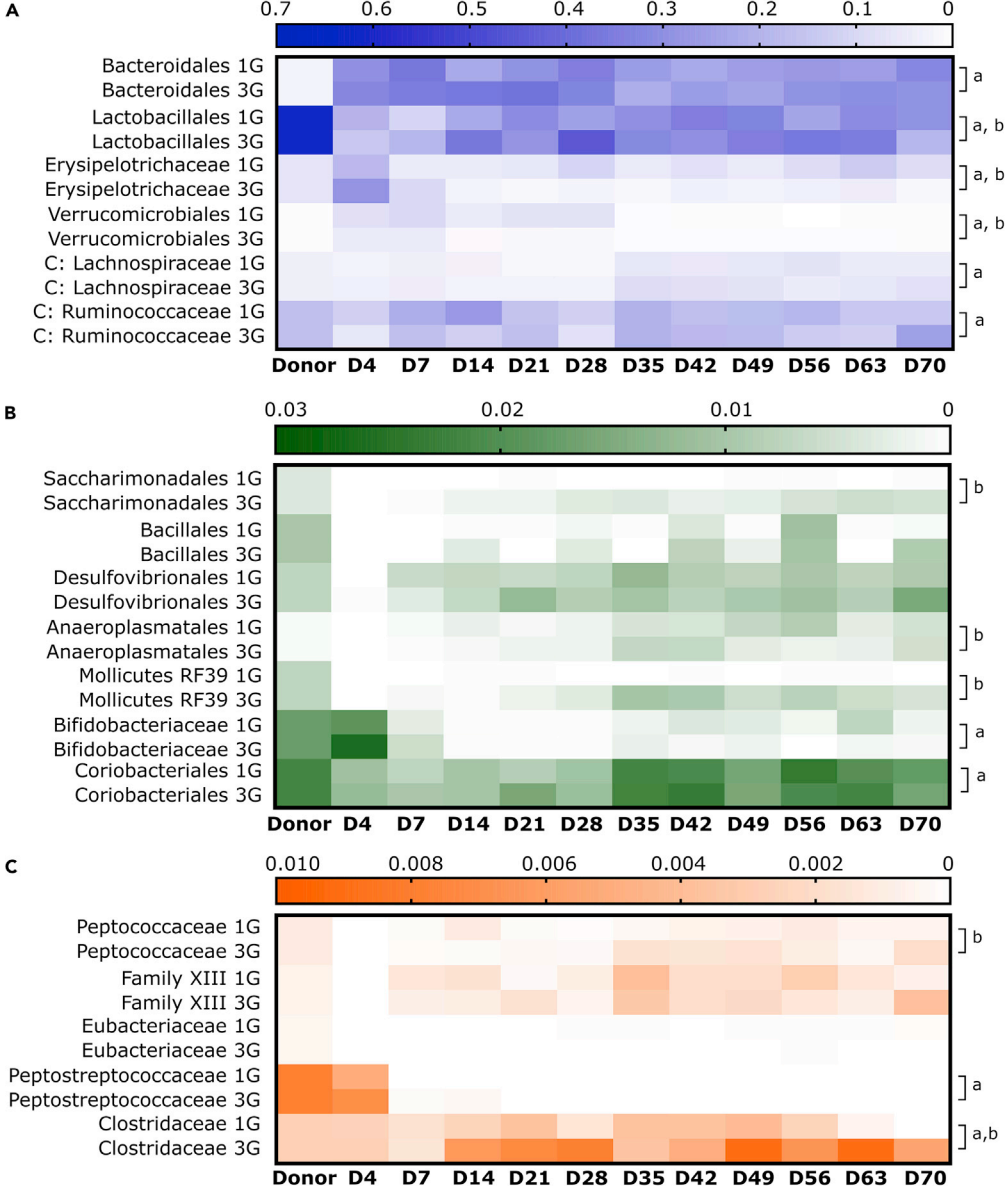
Relative abundance of donor taxa in recipient mice receiving the native microbiota Heatmap of relative abundance of taxa at the order level in recipient that received one (1G) or three gavages (3G) of the native microbiota based on (A) high relative abundance taxa (>0.1 relative abundance) or (B) low relative abundance taxa (<0.1 relative abundance). (C) Bacterial taxa within the Clostridiales order were plotted at the family level. Significant differences between 1G and 3G groups during initial colonization (D4 to D21) or in at least two time points during D28 to D70 were indicated were determined based on a linear mixed-effects model (*lmerTest*) in R (denoted as *a* or *b,* respectively, p < 0.05). Color gradient of the heatmap were based on mean relative abundance values.

Temporal changes in taxon relative abundance differed substantially between phylogenetic clades. For native microbiota, higher donor-recipient compositional distances were observed between D4 to D21 compared to later timepoints, while compositional variation in the single gavage recipients was also higher compared to those receiving multiple gavages. During this period of instability, variations between the single and multiple gavage groups were driven by alterations in the relative abundance of Bacteroidales, Lactobacillales, Erysipelotrichaceae, Verrucomicrobiales, Lachnospiraceae, Bifidobacteriaceae, Coriobacteriales, and Peptostreptococcaceae (p < 0.05) (Figures 6A–6C; line graph in Figure S6). The relative abundances of Lactobacillales, Erysipelotrichaceae, Clostridaceae, Ruminococcaceae, Anaeroplasmatales, Mollicutes RF39, and Peptococcaceae also significantly differed between these groups in at least two or more time points between D28 to D70 (p < 0.05). Bacterial taxa temporal dynamics were broadly consistent between single and multiple gavage groups for other bacterial taxa. When compared to the donor microbiota, the relative abundance of Bacteroidales remained higher, while Lactobacillales were lower in recipients throughout the study period (Figure 6A). The relative abundance of Erysipelotrichaceae and Verrucomicrobiales peaked at D4 following instillation and then declined to near donor levels (Figure 6A), whereas Bifidobacteriaceae and Peptostreptococcaceae declined to D21 and remained low or undetectable thereafter (Figures 6B and 6C). In contrast, Saccharimonadales, Bacillales, Desulfovibrionales, and Anaeroplasmatales were not detectable at D4 but increased gradually over time. Less variation was observed in the relative abundance of other taxa. At the phylum level, the relative abundance of the most prevalent phylum, Firmicutes, was reduced by an average of 30.3% (3G, D70) in the recipient when compared to the donor, while the relative abundance of the second most prevalent phylum, Bacteroidetes, increased by an average of 29.1% (3G, D70) (Figure S7). Phylum-level differences between donor and recipient microbiota were explained largely by the relative abundance of *Lactobacillus* and uncultured members of Muribaculaceae, respectively.

**Figure 7:**
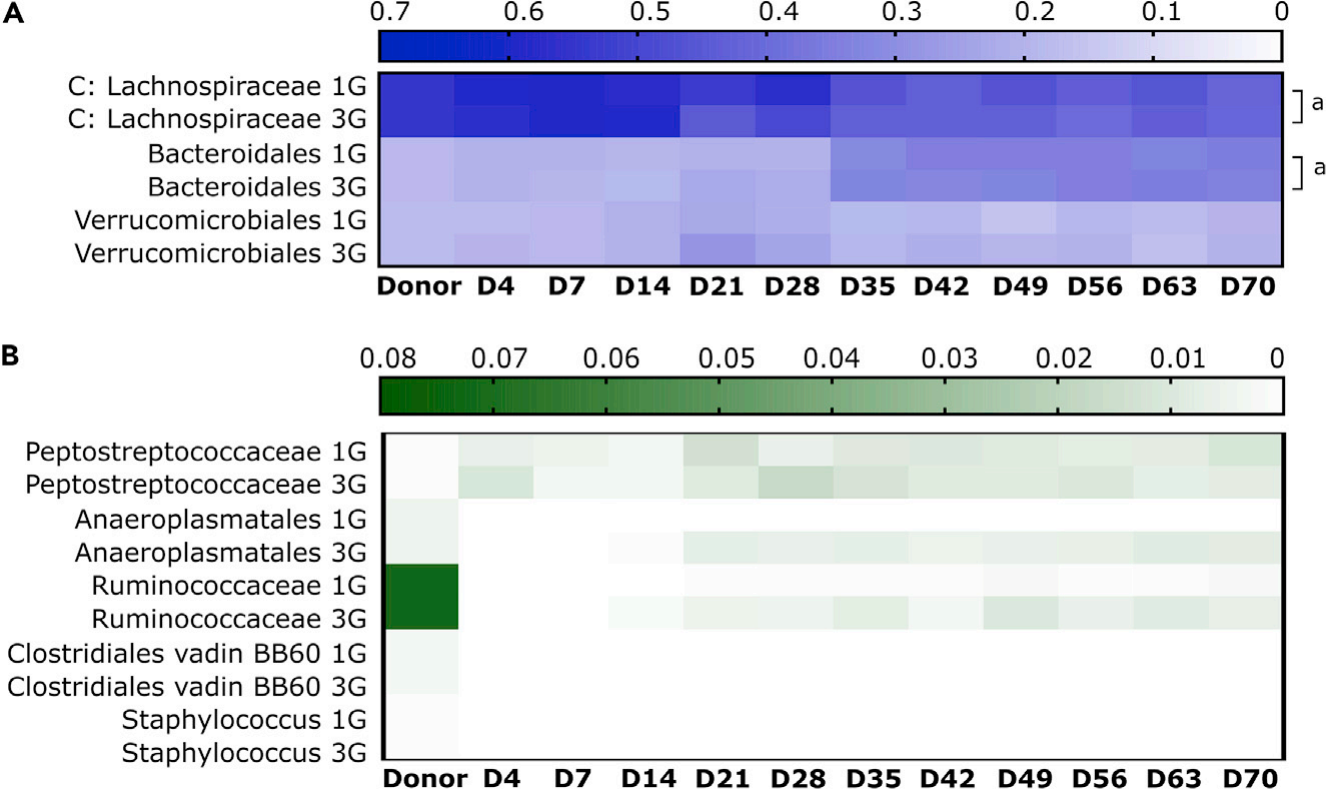
Relative abundance of donor taxa in recipient mice receiving the antibiotic-disrupted microbiota Heatmap of relative abundance of taxa at the order level in recipient that received one (1G) or three gavages (3G) of the antibiotic-disrupted microbiota were plotted at the order level. Bacterial taxa in the Clostridiales order were plotted at the family level, while order levels comprising a single taxa were plotted according to the genus level. Bacterial taxa were plotted according to (A) high relative abundance (>0.1 relative abundance) and (B) low relative abundance taxa (<0.1 relative abundance). Significant differences between 1G and 3G groups during initial colonization (D4 to D21) or in at least two timepoints during D28 to D70 were indicated were determined based on a linear mixed-effects model (*lmerTest*) in R (denoted as *a* or *b,* respectively, p < 0.05). Color gradient of the heatmap were based on mean relative abundance values.

Less variation in bacterial family relative abundance were observed in recipients of antibiotic-disrupted microbiota (Figures 7A and 7B, line graph in Figure S8). The relative abundance of dominant families (Lachnospiraceae, Bacteroidales, and Verrucomicrobiales) were broadly stable throughout the 70 day study, although significant relative abundance differences were observed for Lachnospiraceae and Bacteroidales between single and multiple gavage groups during the initial period of colonization (D4 to D21) (p < 0.05). Ruminococcaceae significantly differed between these groups in at least two or more time points between D28 to D70 (p < 0.05). In contrast to recipients of native donor microbiota, the relative abundance of Ruminococcaceae in both single and multiple gavage groups fell substantially following instillation until D7, before partially recovering, when compared to donor.

### Temporal dynamics in taxon absolute abundance and fecal pH

Quantitative polymerase chain reaction (PCR) was used to determine the absolute abundance of three genera, *Blautia* (Lachnospiraceae)*, Bifidobacterium* (Bifidobacteriaceae), and *Akkermansia* (Verrucomicrobiales), which were the dominant members of bacterial families that showed substantial relative abundance changes in recipients of native or antibiotic-disrupted microbiota (Figure S9). The dynamics of absolute abundance changes for these genera were broadly consistent with their relative abundances. Levels of *Bifidobacterium* and *Akkermansia* declined from D4 and were almost undetectable by D14 in recipients of the native microbiota. In contrast, consistent levels of *Blautia* were observed in recipients of the antibiotic-disrupted microbiota.

As members of the gut microbiota, including *Bifidobacterium,* Clostridaceae, and Peptostreptococcaceae, can strongly influence fecal pH, which in turn can influence the ability of other gut bacteria to grow (Henrick et al., 2018; Ilhan et al., 2017), fecal pH was assessed. Temporal variation in fecal pH was observed in recipients of the native microbiota. Following microbiota instillation at D4, fecal pH decreased from pH 7.1 and pH 7.3 to pH 6.5 and pH 6.4 in the single and multiple gavage groups, respectively (Figure S10). These levels increased by D7 and remained consistent for the multiple gavage group until D28, whereas larger variation across subsequent time points were observed for the single gavage group.

## Discussion

Our goal was to characterize microbiota assembly in gnotobiotic mice following the instillation of donor fecal material. In doing so, we aimed to address a major knowledge gap in our understanding of how microbiota transplantation methodology influences investigations of host-microbiome interactions. Our focus, in particular, was the effect of multiple versus single rounds of gavage, and differences in the dynamics of bacterial colonization when native or substantially disrupted donor microbiota are used.

In keeping with a previous investigation of microbiota establishment in gnotobiotic mice (Gillilland et al., 2012), we observed total levels of fecal bacteria in recipient mice to rapidly come to resemble those of donors. The rate at which instilled microbiota expanded in recipient mice was unaffected by the number of rounds of gavage performed. Increases in bacterial diversity during the early stages of gut colonization are thought to be constrained by ecological succession, rather than by rate of biomass increase, as described in vaginally born human infants (Wampach et al., 2017). This process of succession involves early gut colonizers facilitating the growth of other taxa through modification of growth substrates, production of bioactive metabolites, and alteration of the physicochemical characteristics of the gut environment.

The changes in microbiota characteristics that we observed following instillation into germ-free mice were consistent with the phenomenon of ecological succession. In mice transplanted with native microbiota, dissimilarity to donor microbiota and within-group variance were highest during the initial colonization period, with observed changes in keystone bacterial clades consistent with well-described mechanisms of gut bacterial succession. For example, levels of Bifidobacteriaceae were high initially but became substantially depleted by D14 consistent with the role played by members of this family as primary gut colonizers (Le Roy et al., 2018; Turroni et al., 2012). Bifidobacteria are able to hydrolyze host glycans to release products including glucose, galactose, lactate, and acetate, metabolites that are then utilized by members of the Bacteroidetes and Firmicutes phyla (De Vuyst and Leroy, 2011; Falony et al., 2009). In keeping with these findings, the relative abundance of Bacillales, Lactobacillales, and Peptococcaceae (phylum Firmicutes) were observed to increase from D14 or D28. Additionally, the relative abundance of Desulfovibrionales increased substantially over the first two weeks post-instillation, following an increase in the relative abundance of taxa including Family XIII, Clostridaceae, Peptococcaceae, and Lachnospiraceae. Several genera within these families are potential butyrate-producers (Chai et al., 2019). Many members of Desulfovibrionales are sulfate-reducers, which utilizes hydrogen gas, which are produced during the biosynthesis of SCFAs. Levels of *Desulfovibrio*, for example, have been reported to increase with levels of butyrate (El Aidy et al., 2013). Notably, changes in microbiota structure during early colonization with native microbiota were reflected in changes in fecal pH. The rapid decrease in pH from D4 post-colonization, followed by an increase thereafter, might be explained by the high levels of *Bifidobacterium* observed, a taxon capable of the central hexose fermentation pathway (bifid shunt) to produce lactate, which reduces fecal pH (Fushinobu, 2010; Baxter et al., 2019). We did not encounter overgrowth of individual opportunistic taxa in recipient animals, such as the genera *Escherichia* (phylum Proteobacteria) as reported previously (Gillilland et al., 2012; El Aidy et al., 2012, 2013). Gillilland et al. (2012) also reported an overgrowth of *Escherichia*, markedly at day 1, which levels decreased by day 7, while Proteobacteria were not reported in 14-day post-colonization samples of the ileum and distal colon mucosa (Johansson et al., 2015), or 21-day post-colonization fecal samples (Le Roy et al., 2018). As our earliest microbiota assessment is at day 4, we cannot therefore exclude the possibility of this phenomenon prior to this time point. However, the lack of Proteobacteria bloom in recipient mice may be attributed to the microbial community of animals within the facility, which we reported previously to have significantly lower levels of taxa in the Proteobacteria phylum compared to other local facilities (Choo et al., 2017).

Approaches used in the colonization of germ-free mice as part of published studies vary considerably. Some investigators have employed single rounds of gavage (Ridaura et al., 2013; Blanton et al., 2016), while others have used multiple (typically three) rounds (Ericsson et al., 2017; Gopalakrishnan et al., 2018). Disappointingly, many other studies do not describe donor material preparation or the number of instillations that were performed. In our study, multiple rounds of gavage were associated with modest but consistent increases in the representation of donor taxa in recipients, both where native or antibiotic-disrupted microbiota were instilled. These differences are attributable to several bacterial taxa that were detected in recipients of multiple rounds of gavage but not in recipients of single gavage of the native microbiota, including *Jeotgalicoccus* (Bacillales order)*, Lachnospiraceae UC5-1-2E3,* and Mollicutes RF39. Taxa within the Bacillales, Peptococcaceae, and Mollicutes RF39 order also peaked in relative abundance by D14 to D35 onward in recipients of multiple gavage with the native microbiota but not in single gavage recipients. Multiple gavages was also associated with reduced inter-animal variance, an important factor when using such animals to investigate host-microbiota interactions.

Persistent differences between donor and recipient microbiota composition were evident, notably with a reduction in the relative abundance of the predominant phylum, Firmicutes, and an increase in the relative abundance of Bacteroidetes. In addition, several bacterial genera were detected in donor microbiota but not in transplant recipients, including *Anaerofustis, Bacteroides,* and *Erysipelothrix*. Why such differences should occur between mice of similar genetic backgrounds is not clear; however, there are a number of potential contributory factors. For example, the donor transplant material were derived from the cecum, while recipient microbiota composition was assessed based on fecal pellets. Differences in gut physiology in gnotobiotic mice compared with conventional mice have also been described, including in immune mechanisms that are involved in the regulation of intestinal microbiology (Blanton et al., 2016; Pollard and Sharon, 1970; Wichmann et al., 2013; Manca et al., 2020; Husebye et al., 2001; Thompson and Trexler, 1971), which could influence colonization.

In addition to investigating microbiota assembly in mice transplanted with native microbiota, we also assessed colonization dynamics following the instillation of microbiota from antibiotic-disrupted animals. In doing so, our aim was to determine whether the dynamics of conventionalization with substantially disrupted microbiota, as associated with a number of pathophysiological contexts (Ridaura et al., 2013; Blanton et al., 2016), differs to those with native gut microbiota. The donor microbiota that resulted from antibiotic disruption exhibited substantially reduced taxa richness compared to the native microbiota, in which several taxa were either absent (*Anaerostipes,* Clostridiales vadin BB60 group, *Intestinimonas*), or present at low abundance (<1%) (*Alistipes, Eubacterium xylanophilum, Acetatifactor, Blautia, Ruminiclostridium, Anaeroplasma, Akkermansia*). Compared to native microbiota, instillation of antibiotic-disrupted microbiota was associated with greatly reduced temporal variation. Relative abundance of the predominant taxa, including Lachnospiraceae, Bacteroidales, and Verrucomicrobiales, achieved levels comparable to those in donor material upon instillation and showed little variation over time. Divergence from donor composition was driven by a failure to recover less prevalent taxa, including *Staphylococcus*, *Clostridiales vadin BB60*, and *Alistipes* (Bacteroidales order), as well as multiple members of the Lachnospiraceae (*Acetatifactor, Lachnoclostridium,* Lachnospiraceae UCG-006, *Eubacterium xylanophilum*) and Ruminococcaceae families (*Rumimiclostridium*) also failed to colonize recipient animals by D4 (either single or multiple gavage). The genera *Intestinimonas* and *Anaeroplasma*, which are of the Ruminococcaceae and Anaeroplasmatales families, respectively, were detected only in multiple gavage recipients. As these taxa were still detected in the fecal microbiota of recipients that received the native microbiota, it is unlikely that the failure to detect these taxa in the antibiotic-disrupted recipients were due to selective characteristics of the cecum and luminal colon in the donor and recipient mice, respectively. The detection of these taxa in the donor could represent bacterial taxa that were largely nonviable due to antibiotic exposure. Failure of selected taxa to re-establish in the recipient can also be attributed to competitive interactions and compensatory relationships between bacterial species. The re-establishment of selected donor taxa can be inhibited by novel interactions that form within an altered microbial community to generate an alternate stable equilibrium (Shashkova et al., 2016), a phenomenon that is also observed in long-term post-antibiotic studies in humans (Dethlefsen and Relman, 2011; Jernberg et al., 2007). Additionally, the members within the Lachnospiraceae and Ruminococcaceae families may provide compensatory function, including their role in producing important metabolites such as short chain fatty acids by degrading complex polysaccharides (Biddle et al., 2013).

In our study, cecal microbiota were harvested and processed under strict anaerobic conditions. Alternative approaches that utilize stool may influence the bacterial taxa that are represented, while preparations that involve exposure to aerobic conditions are likely to be associated with lower levels of donor-recipient similarity. Furthermore, our transplantation was between members of the same species (C57BL/6 mice), subject to similar environmental and dietary exposures. There is a growing number of studies involving the instillation of human stool, or stool-derived microbiota, into gnotobiotic mice. Substantial differences in genetics (Ericsson et al., 2017), age (Le Roy et al., 2018; Langille et al., 2014), type of housing (Rausch et al., 2016), and diet (autoclave/irradiation) (Rausch et al., 2016; Carmody et al., 2015), which are among the factors that shape the gut microbiota, are expected to further influence associated levels of microbiota recapitulation. Previous studies have shown that transfer of microbial community with a higher diversity from BHsd mice, into B6J mice that had a simpler microbiota, was more effective compared to the reciprocal procedure (Ericsson et al., 2017). Additionally, resemblance of the microbiota to the inoculum was higher when instilled into microbiota-depleted conventional mice at a younger age at 3-wk-old, compared to those at 8-wk-old (Le Roy et al., 2018).

In summary, a substantial period (more than 4 weeks) may be required following fecal instillation into germ-free mice to achieve closest compositional similarity to the donor for the native microbiota. Establishment of donor microbiota occurs more rapidly in less diverse bacterial communities that result from antibiotic exposure. The process of microbiota assembly differs considerably based on the complexity and composition of donor bacterial communities. Multiple rounds of fecal instillation result in greater similarity to donor microbiota for the native microbiota. Using the same regimen for microbiota instillation involving an altered microbial community did not improve donor-recipient microbiota similarity. A failure to understand the extent to which donor microbiota has been established in recipient mice and the degree of community stability achieved could contribute to spurious findings.

### Limitations of the study

Our study had a number of limitations. The dynamics of microbiota changes at the species level could not be assessed, as the highest resolution of taxa classification by 16S rRNA sequencing is to the genera level. We performed donor taxa representation by longitudinal fecal microbiota assessments of the recipients against the donor cecal material, which may influence taxa detection due to site differences. We also did not assess all possible gavage schedules, donor/recipient microbiota variants, cage arrangements, age and gender specificities but rather examined overall features of microbiota establishment in recipients. Microbiota assembly dynamics are likely to change with donor microbiome characteristics and can therefore vary with mouse genetic background (Korach-Rechtman et al., 2019) and between populations housed at different research facilities (Choo et al., 2017; Rausch et al., 2016). What host measures are being assessed, and what the hypothesized mechanisms of host-microbiome interaction are, will also be important considerations in the design of experiments involving transplanted microbiota.

### Resource availability

#### Lead contact

Further information and requests for resources should be directed to and will be fulfilled by the lead contact, Jocelyn Choo (jocelyn.choo@sahmri.com)

#### Materials availability

The study did not generate new unique reagents.

#### Data and code availability

The accession number for the sequence data reported in this paper is SRA: PRJNA592263.

## Methods

All methods can be found in the accompanying Transparent methods supplemental file.

## Supplemental information

Document S1. Transparent methods, Figures S1–S10, and Tables S1 and S2
