# Supplementary material for: Establishment of murine gut microbiota in gnotobiotic mice
Source: iScience. 2021 Jan 12;24(2):102049. doi: 10.1016/j.isci.2021.102049 (PMC7840467; doi:10.1016/j.isci.2021.102049)
Supplement: Document S1. Transparent methods, Figures S1–S10, and Tables S1 and S2 [file mmc1.pdf]

iScience, Volume *24*

Supplemental Information

Establishment of murine
gut microbiota in gnotobiotic mice

Jocelyn M. Choo and Geraint B. Rogers

SUPPLEMENTAL INFORMATION

SUPPLEMENTAL FIGURE AND TABLES

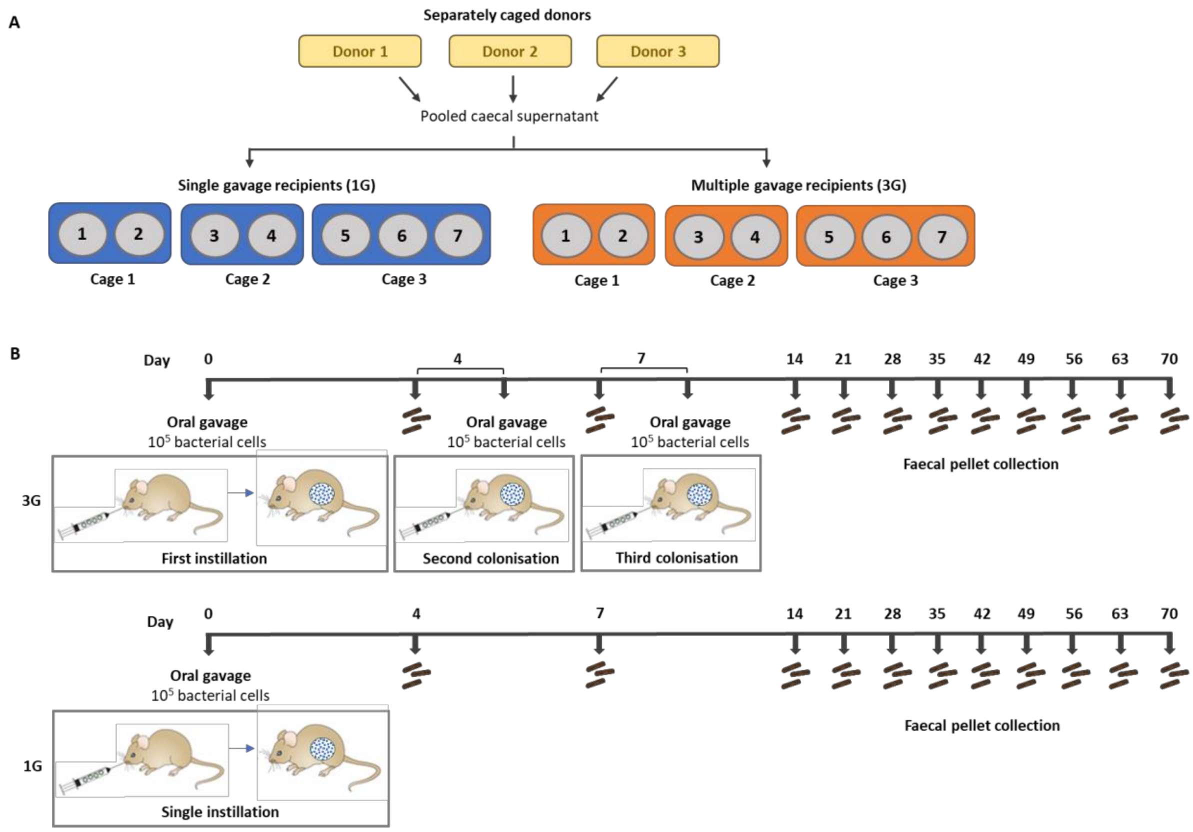

Figure S1. Study design for the establishment of gut microbiota in germ-free mice. Related to Figures 1 – 7. (A) Cecal material harvested from three separately housed donor C57BL/6 mice anaerobically, and the cecal suspension were pooled as inoculum material for instillation into recipient C57BL/6 germ-free mice of the single (1G) or multiple gavage (3G) group. Each group (n=7 mice) comprised of three cages, with each cage containing two to three mice. (B) Recipient C57BL/6 germ-free mice received either one (1G) or three (3G) rounds of anaerobically-prepared pooled cecal suspension containing approximately 10^5 bacterial cells. Fecal pellets were collected from individual mice throughout the experiment.

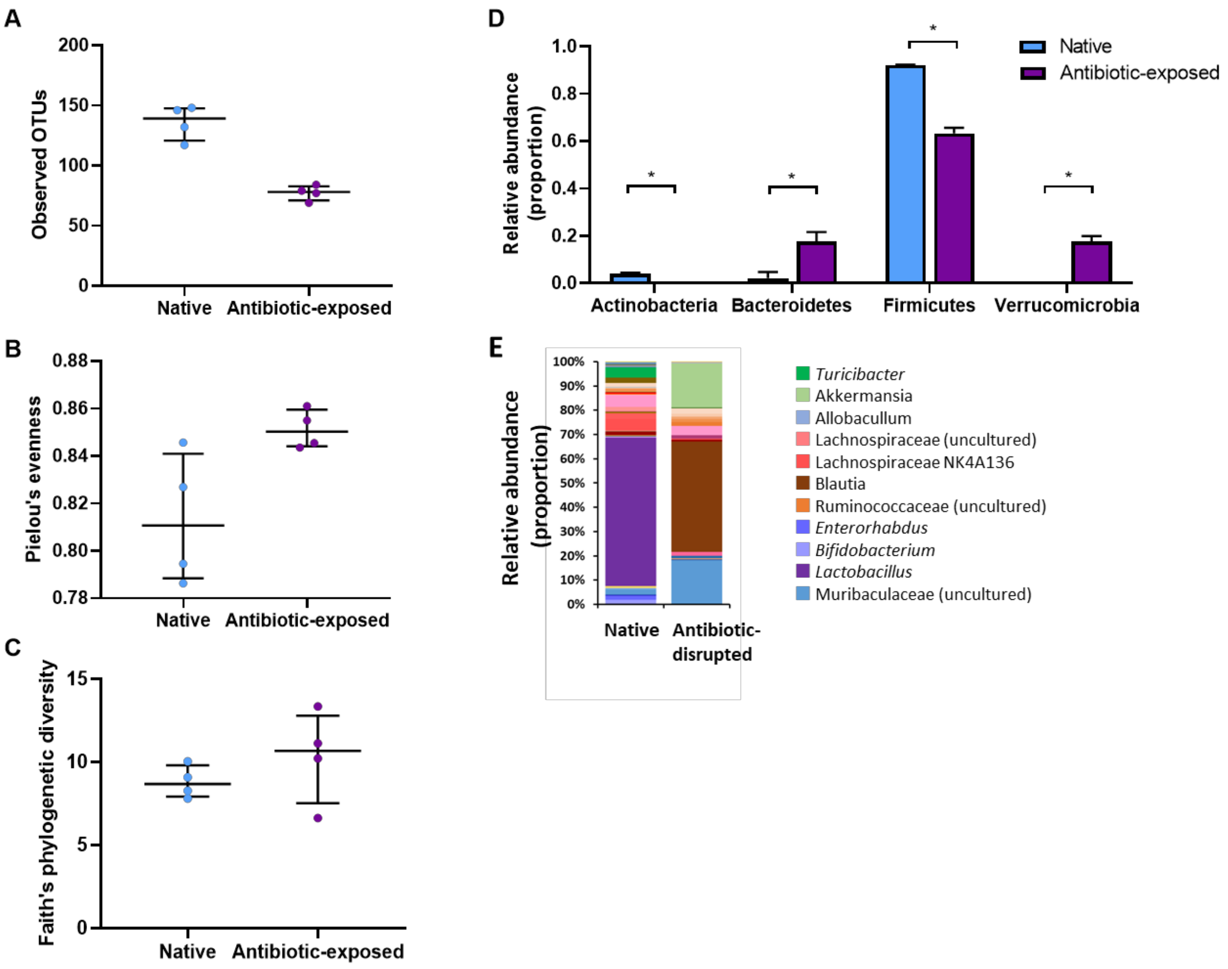

Figure S2. Alpha diversities and phylum-level relative abundances of donor microbiota. Related to Figure 1. Alpha diversity measures of (A) observed operational taxonomic units (OTUs), (B) Pielou's evenness and (C) Faith's phylogenetic diversity of the native and antibiotic-disrupted microbiota of the donor were determined using QIIME2. (D) Phylum-level relative abundances (relative abundance >1%) and (E) genus-level relative abundances of the native and antibiotic-disrupted donor microbiota were determined. The median values are plotted and the error bars represent the interquartile ranges. Statistical comparisons were performed using the Mann-Whitney test at a level of $P < 0.05$ (denoted as asterisk).

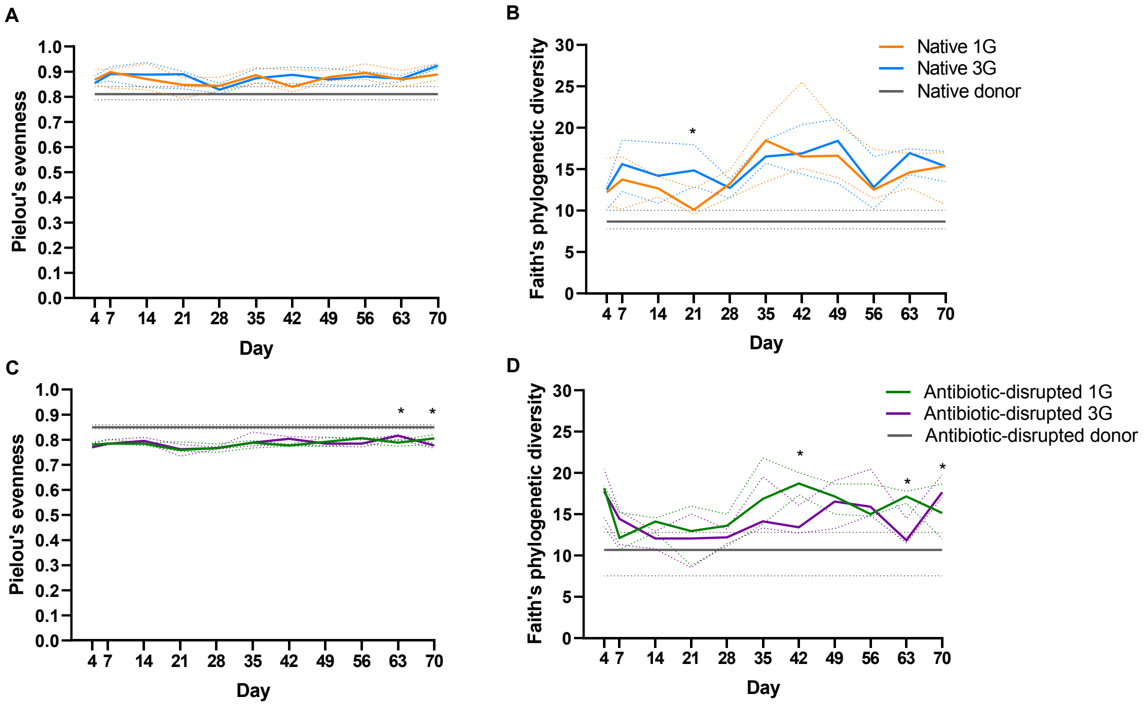

Figure S3. Alpha diversity of recipient microbiota. Related to Figures 2 and 3. Alpha diversity measures of Pielou's evenness and Faith's phylogenetic diversity of the native (A and B, respectively) and antibiotic-disrupted microbiota of recipients and donor (C and D, respectively) were determined using QIIME2. Solid lines denote median values, while the dotted lines denote interquartile ranges. Statistical differences between single (1G) or multiple gavage (3G) groups at each timepoint were determined using a linear mixed-effects model (*lmerTest*) with Bonferroni correction at P< 0.05 (denoted as asterisk).

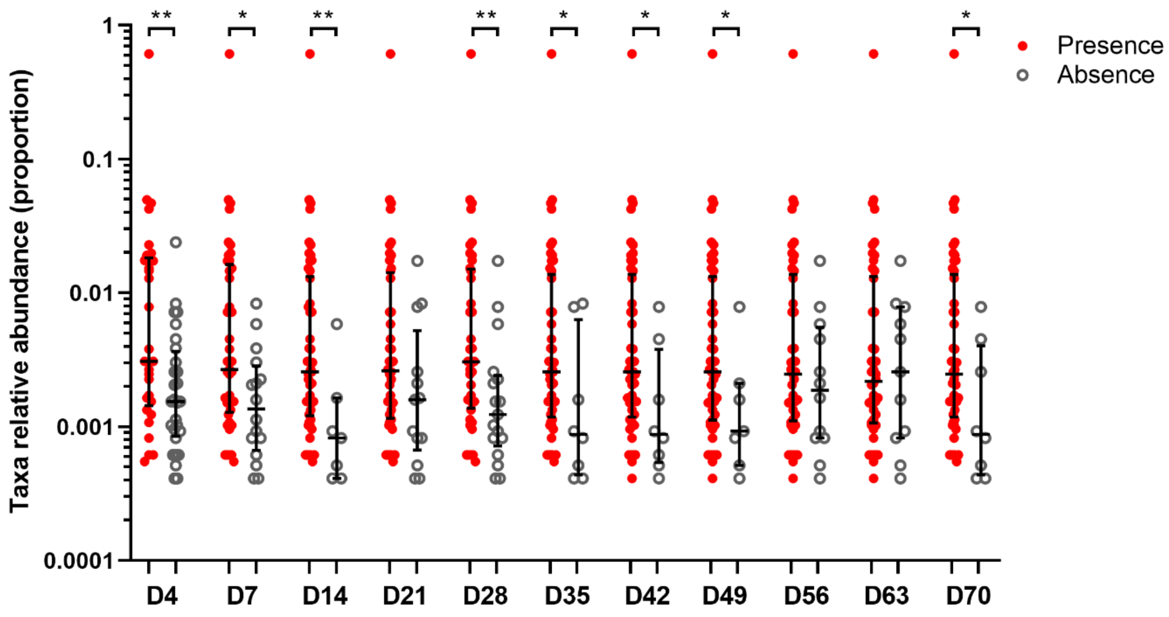

Figure S4. Relative abundance of donor bacterial taxa in recipient mice following fecal gavage. Related to Figure 2. Relative abundance of donor taxa that were either present (solid circles) or absent (open circles) in recipient mice that received three rounds of fecal gavage with intact native microbiota. The middle and error bars indicate the median and interquartile ranges, respectively. Statistical comparison between the relative abundance levels of consistent and intermittent taxa were performed using the Mann-Whitney test at a level of P< 0.05 for significance.

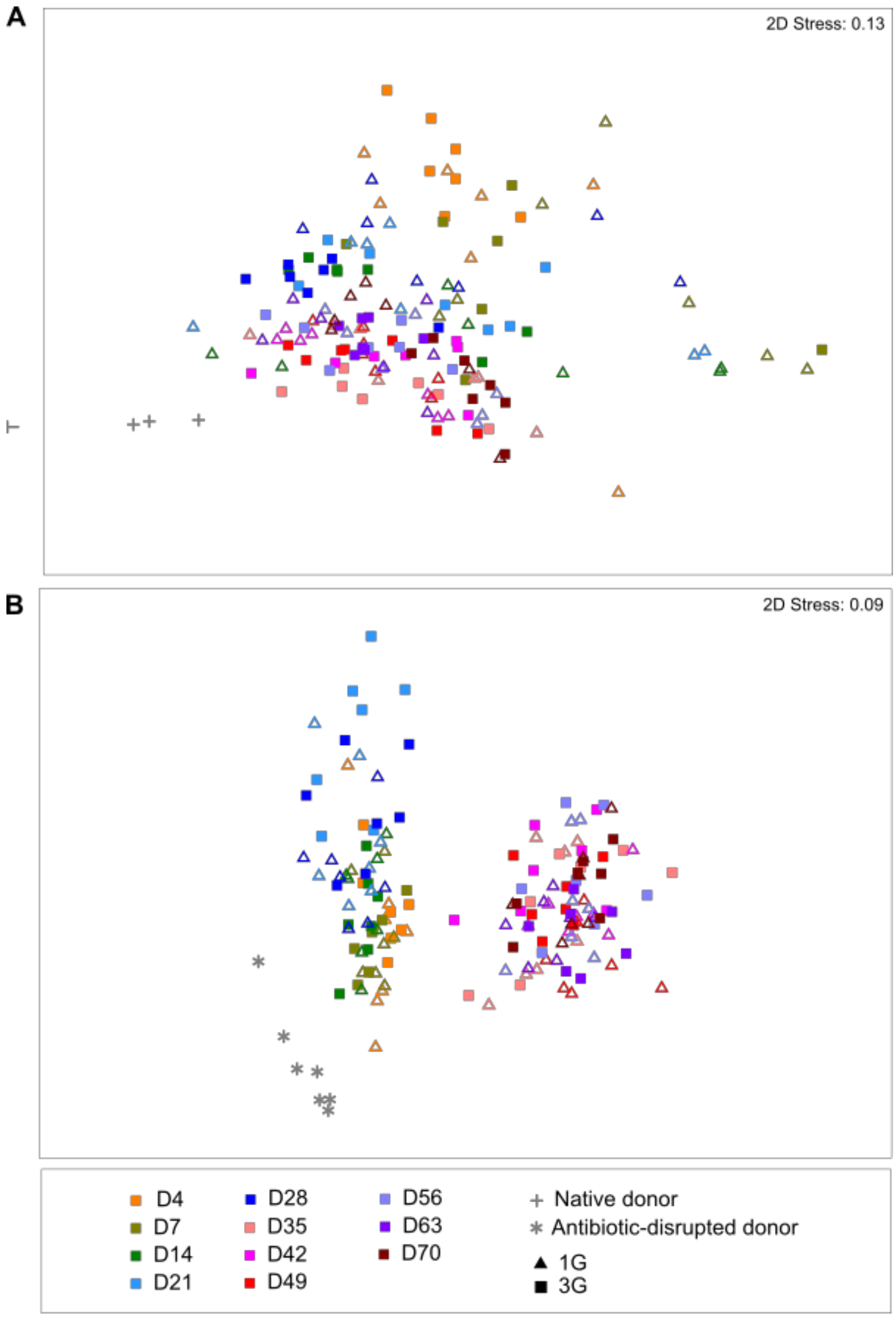

Figure S5. Non-metric multidimensional scaling (NMDS) plot of donor and recipient microbiota. Related to Figures 4 and 5. Weighted UniFrac distances of donor and recipient samples of the (A) native and (B) antibiotic-disrupted microbiota computed using QIIME2 were used for sample ordination. Recipient mice received either single (1G) or multiple gavages (3G) of the donor inoculum. Samples for each timepoint are shown as the colours indicated in the legend.

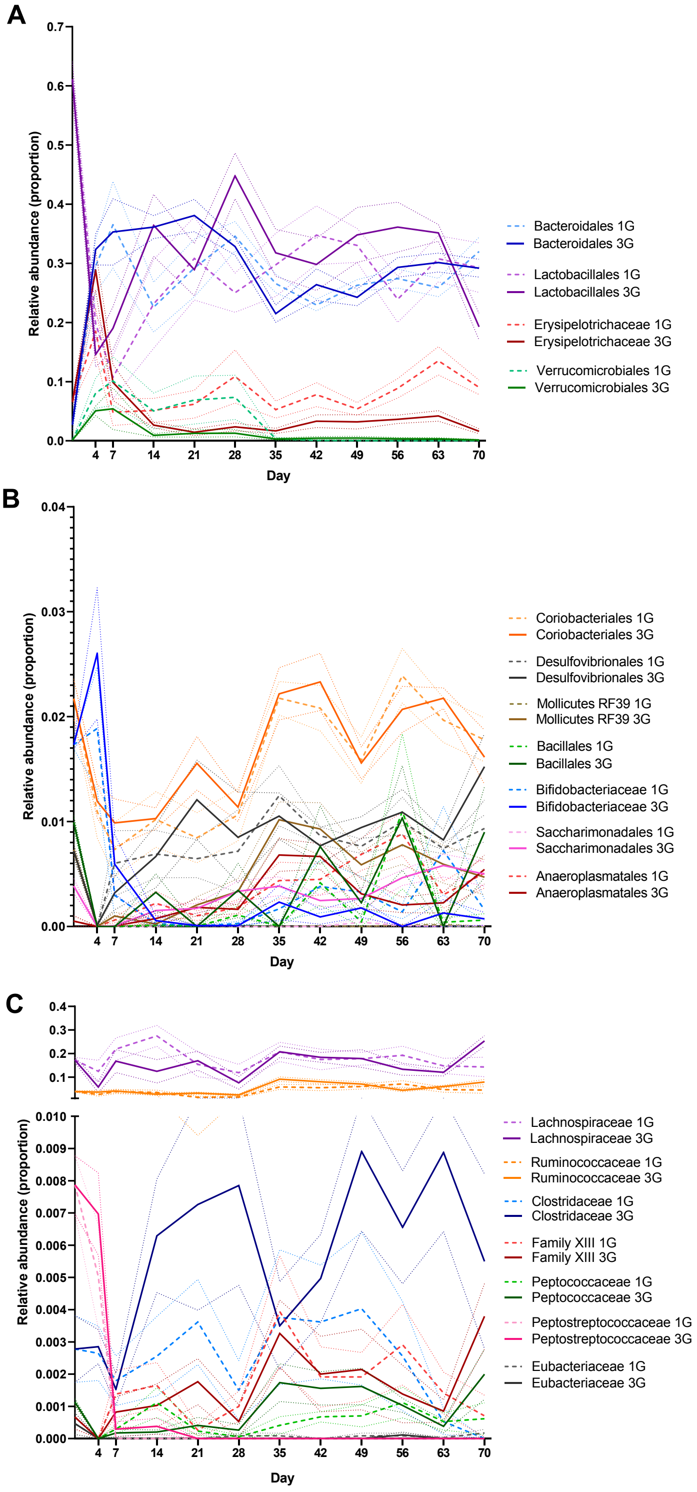

Figure S6. Relative abundance of donor taxa in recipient mice receiving the native microbiota. Related to Figure 6. Donor taxa observed in recipient that received one (1G) or three gavages (3G) of intact native microbiota were plotted at the order level based on (A) high relative abundance taxa (>0.03 relative abundance), (B) low relative abundance taxa (<0.03 relative abundance). (C) Bacterial taxa within the Clostridiales order were plotted at the family level. Solid and dotted lines denote the mean ± standard error of mean (SEM) values.

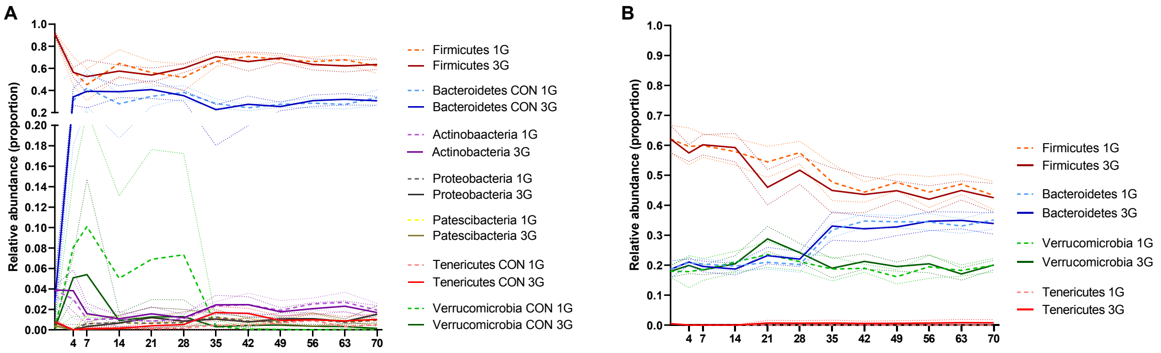

Figure S7. Relative abundance of donor taxa at the phylum level in recipient mice. Related to Figure 6. Relative abundances of bacterial phyla (present in donor at a relative abundance of >0.01) in recipient mice that received one (1G) or three gavages (3G) of the (A) native or (B) antibiotic-disrupted microbiota. Solid and dotted lines denote the mean ± standard error of mean (SEM) values.

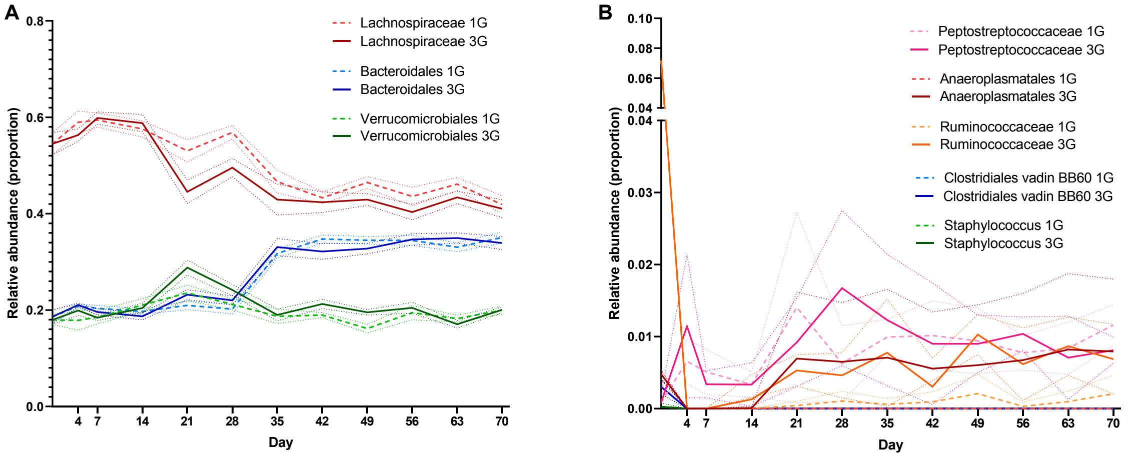

Figure S8. Relative abundance of donor taxa in recipient mice receiving the antibiotic-exposed microbiota. Related to Figure 7. Donor taxa observed in recipient that received one or three gavages of the antibiotic-exposed microbiota were plotted at the order level, except for bacterial taxa in the Clostridiales order, which were plotted at the family level. Bacterial taxa were plotted according to (A) high relative abundance (>0.03 relative abundance) and (B) low relative abundance taxa at the order level (<0.03 relative abundance). Recipient mice received either one round (1G) or three rounds (3G) of donor microbiota. Solid and dotted lines denote the mean ± standard error of mean (SEM) values.

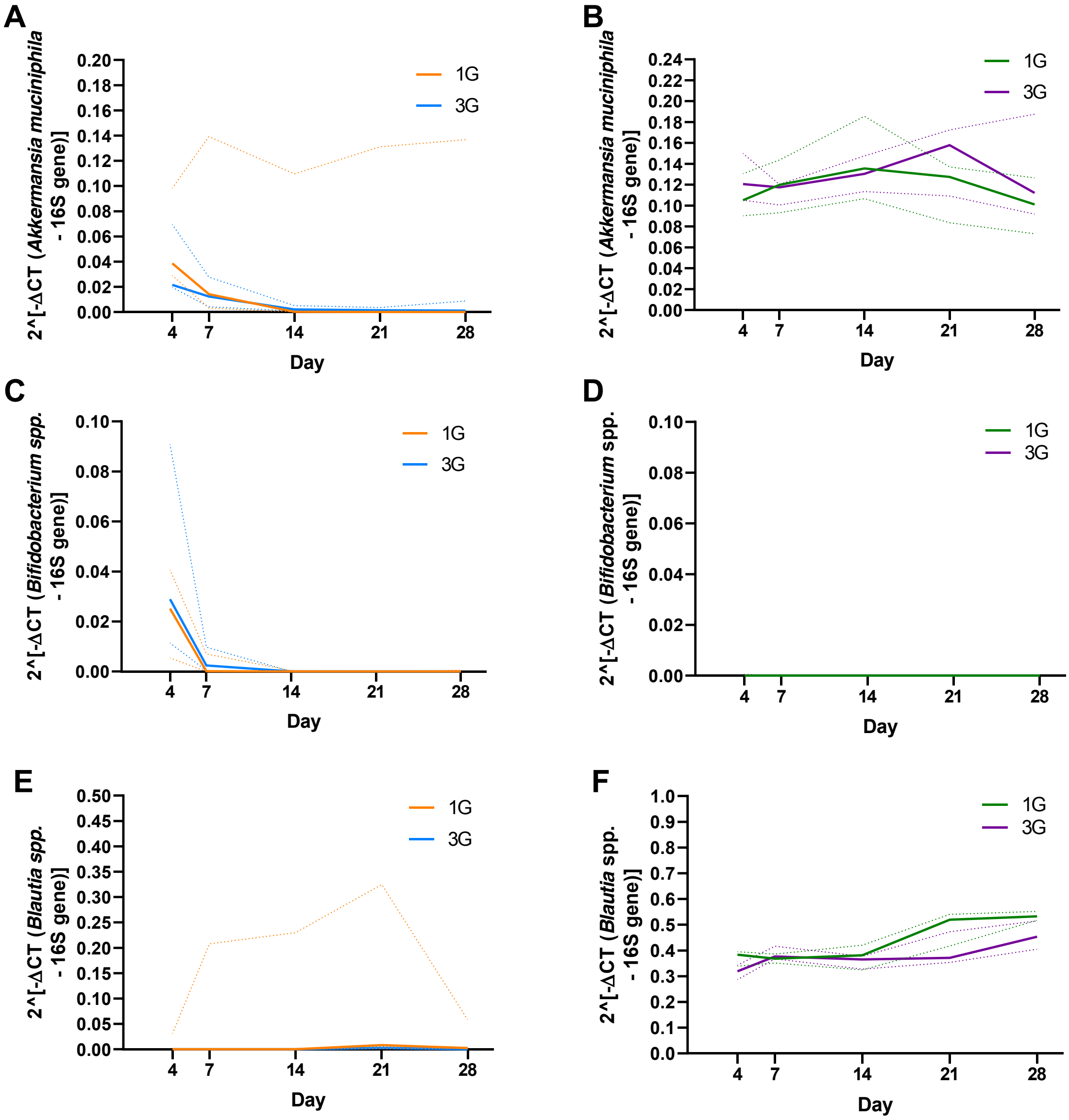

Figure S9. Absolute abundance levels of selected taxa in recipient mice. Related to Figures 6 and 7. The absolute levels of (A, B) *Akkermansia muciniphila* (C, D) *Bifidobacterium* spp. and *Blautia* spp. (E, F) were determined by quantitative polymerase chain reaction (qPCR) in recipient mice that received one (1G) or three gavages (3G) of the native or antibiotic-disrupted microbiota, respectively. Absolute abundances of each bacterial taxa were normalised against the total bacterial load in the sample based on the delta cycle threshold (CT) values of the target gene and 16S rRNA gene. Solid and dotted lines denote the median value and interquartile ranges, respectively.

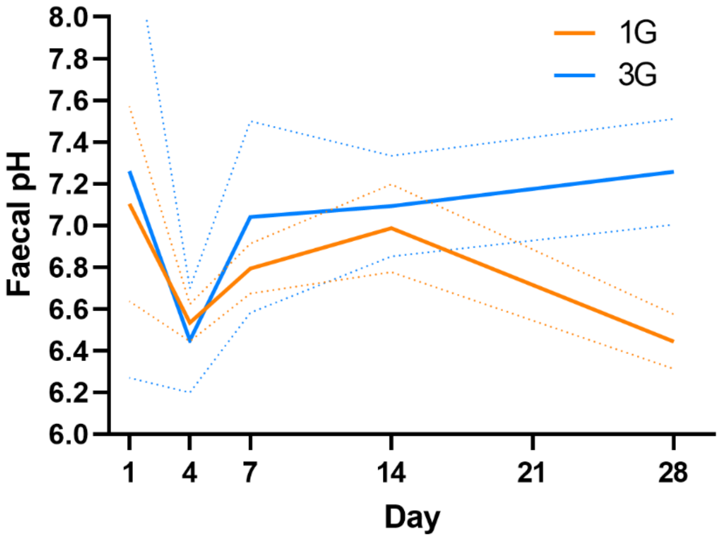

Figure S10. Fecal pH levels in recipient mice of the native microbiota. Related to Figures 6 and 7. Fecal samples of recipient mice of the native microbiota at each timepoint were pooled according to their cages and the pH levels analysed. Recipient mice received either one round (1G) or three rounds (3G) of donor microbiota. Solid and dotted lines denote the mean value and standard deviation of pH levels, respectively.

Table S1. Genus-level relative abundance of bacterial taxa in recipient mice receiving the native microbiota. Related to Figure 6. Phylogenetic classification and relative abundance (genus level) of operational taxonomic units (OTUs) in donor and recipients of the single gavage (1G) and multiple gavage (3G) groups for the native microbiota.

**Phylogenetic classification**			
**Phylum**	**Class**	**Family**	**Order**
Actinobacteria	Actinobacteria	Bifidobacteriales	Bifidobacteriaceae
	Coriobacteriia	Coriobacteriales	Eggerthellaceae
	Coriobacteriia	Coriobacteriales	Eggerthellaceae
	Coriobacteriia	Coriobacteriales	Eggerthellaceae
Bacteroidetes	Bacteroidia	Bacteroidales	Bacteroidaceae
	Bacteroidia	Bacteroidales	Muribaculaceae
	Bacteroidia	Bacteroidales	Muribaculaceae
	Bacteroidia	Bacteroidales	Muribaculaceae
	Bacteroidia	Bacteroidales	Prevotellaceae
	Bacteroidia	Bacteroidales	Rikenellaceae
Firmicutes	Bacilli	Bacillales	Staphylococcaceae
	Bacilli	Bacillales	Staphylococcaceae
	Bacilli	Lactobacillales	Lactobacillaceae
	Clostridia	Clostridiales	Clostridiaceae 1
	Clostridia	Clostridiales	Clostridiaceae 1
	Clostridia	Clostridiales	Eubacteriaceae
	Clostridia	Clostridiales	Family XIII
	Clostridia	Clostridiales	Family XIII
	Clostridia	Clostridiales	Lachnospiraceae
	Clostridia	Clostridiales	Lachnospiraceae
	Clostridia	Clostridiales	Lachnospiraceae
	Clostridia	Clostridiales	Lachnospiraceae
	Clostridia	Clostridiales	Lachnospiraceae
	Clostridia	Clostridiales	Lachnospiraceae
	Clostridia	Clostridiales	Lachnospiraceae
	Clostridia	Clostridiales	Lachnospiraceae
	Clostridia	Clostridiales	Lachnospiraceae
	Clostridia	Clostridiales	Lachnospiraceae
	Clostridia	Clostridiales	Lachnospiraceae
	Clostridia	Clostridiales	Lachnospiraceae
	Clostridia	Clostridiales	Lachnospiraceae
	Clostridia	Clostridiales	Peptococcaceae
	Clostridia	Clostridiales	Peptostreptococcaceae
	Clostridia	Clostridiales	Ruminococcaceae
	Clostridia	Clostridiales	Ruminococcaceae
	Clostridia	Clostridiales	Ruminococcaceae
	Clostridia	Clostridiales	Ruminococcaceae
	Clostridia	Clostridiales	Ruminococcaceae
	Clostridia	Clostridiales	Ruminococcaceae
	Clostridia	Clostridiales	Ruminococcaceae
	Clostridia	Clostridiales	Ruminococcaceae
	Clostridia	Clostridiales	Ruminococcaceae
	Clostridia	Clostridiales	Ruminococcaceae
	Clostridia	Clostridiales	Ruminococcaceae
	Clostridia	Clostridiales	Ruminococcaceae
	Clostridia	Clostridiales	Ruminococcaceae
	Erysipelotrichia	Erysipelotrichales	Erysipelotrichaceae
	Erysipelotrichia	Erysipelotrichales	Erysipelotrichaceae
	Erysipelotrichia	Erysipelotrichales	Erysipelotrichaceae
	Erysipelotrichia	Erysipelotrichales	Erysipelotrichaceae
Patescibacteria	Saccharimonadia	Saccharimonadales	Saccharimonadaceae
Proteobacteria	Deltaproteobacteria	Desulfovibrionales	Desulfovibrionaceae
Tenericutes	Mollicutes	Anaeroplasmatales	Anaeroplasmataceae
	Mollicutes	Mollicutes RF39	unknown taxa
	Mollicutes	Mollicutes RF39	Ambiguous_taxa
Verrucomicrobia	Verrucomicrobiae	Verrucomicrobiales	Akkermansiaceae

* Sequence variants present in donor taxa at a relative abundance of ≥ 0.0004

	Donor				Native microbiota, single gav	
					1G D4	
Genus	1	2	3	pooled	M1	M2
Bifidobacterium	0.0167	0.0183	0.0167	0.0177	0.0177	0.0183
unknown taxa	0.0021	0.0103	0.0029	0.0029	0.0000	0.0000
DNF00809	0.0025	0.0025	0.0031	0.0023	0.0000	0.0000
Enterorhabdus	0.0132	0.0136	0.0169	0.0148	0.0123	0.0198
Bacteroides	0.0000	0.0004	0.0000	0.0006	0.0000	0.0000
unknown taxa	0.0037	0.0012	0.0033	0.0070	0.0340	0.0105
Muribaculum	0.0031	0.0000	0.0029	0.0033	0.0035	0.0031
uncultured bacterium	0.0154	0.0072	0.0150	0.0418	0.2688	0.1519
Prevotellaceae UCG-001	0.0000	0.0006	0.0000	0.0021	0.0000	0.0004
Alistipes	0.0004	0.0000	0.0000	0.0008	0.0014	0.0000
Jeotgalicoccus	0.0016	0.0023	0.0014	0.0010	0.0000	0.0000
Staphylococcus	0.0074	0.0088	0.0072	0.0099	0.0000	0.0000
Lactobacillus	0.6094	0.6598	0.6470	0.5289	0.2663	0.2017
Candidatus Arthromitus	0.0014	0.0000	0.0000	0.0016	0.0000	0.0000
Clostridium sensu stricto 1	0.0033	0.0000	0.0025	0.0023	0.0043	0.0039
Anaerofustis	0.0014	0.0000	0.0004	0.0000	0.0000	0.0000
Eubacterium brachy group	0.0000	0.0008	0.0000	0.0012	0.0000	0.0000
Family XIII UCG-001	0.0000	0.0000	0.0002	0.0004	0.0000	0.0000
unknown taxa	0.0587	0.0389	0.0502	0.0508	0.0049	0.0387
Eubacterium xylanophilum group	0.0021	0.0000	0.0016	0.0008	0.0000	0.0000
Acetatifactor	0.0014	0.0000	0.0004	0.0012	0.0000	0.0000
Blautia	0.0021	0.0027	0.0019	0.0058	0.0012	0.0012
Lachnospiraceae GCA-900066575	0.0000	0.0012	0.0019	0.0000	0.0002	0.0029
Lachnoclostridium	0.0210	0.0165	0.0165	0.0161	0.0156	0.0373
Lachnospiraceae FCS020 group	0.0027	0.0037	0.0014	0.0021	0.0049	0.0088
Lachnospiraceae NK4A136 group	0.0482	0.0403	0.0407	0.0587	0.0045	0.0086
Lachnospiraceae UCG-001	0.0000	0.0025	0.0000	0.0027	0.0000	0.0000
Lachnospiraceae UCG-006	0.0268	0.0208	0.0224	0.0257	0.0000	0.0014
Roseburia	0.0000	0.0000	0.0000	0.0000	0.0000	0.0000
Lachnospiraceae UC5-1-2E3	0.0054	0.0056	0.0045	0.0080	0.0000	0.0000
uncultured	0.0212	0.0156	0.0158	0.0239	0.0031	0.0457
uncultured	0.0010	0.0010	0.0014	0.0010	0.0000	0.0000
Romboutsia	0.0064	0.0072	0.0074	0.0105	0.0076	0.0070
unknown taxa	0.0150	0.0171	0.0142	0.0148	0.0037	0.0023
Eubacterium coprostanoligenes group	0.0000	0.0002	0.0004	0.0006	0.0134	0.0364
Butyricicoccus	0.0008	0.0000	0.0016	0.0000	0.0000	0.0027
Ruminococcaceae GCA-900066225	0.0008	0.0019	0.0014	0.0025	0.0004	0.0012
Oscillibacter	0.0000	0.0008	0.0006	0.0014	0.0000	0.0000
Ruminiclostridium 5	0.0109	0.0117	0.0105	0.0183	0.0000	0.0000
Ruminiclostridium 6	0.0000	0.0000	0.0012	0.0004	0.0000	0.0000
Ruminiclostridium 9	0.0027	0.0023	0.0021	0.0014	0.0000	0.0000
Ruminococcaceae UCG-005	0.0000	0.0000	0.0002	0.0021	0.0000	0.0000
Ruminococcaceae UCG-010	0.0000	0.0000	0.0006	0.0000	0.0000	0.0000
Ruminococcaceae UCG-013	0.0000	0.0000	0.0023	0.0008	0.0035	0.0006
Ruminococcaceae UCG-014	0.0039	0.0010	0.0033	0.0039	0.0000	0.0000
Ruminococcaceae UBA1819	0.0006	0.0008	0.0004	0.0000	0.0000	0.0000
Erysipelothrix	0.0000	0.0004	0.0000	0.0000	0.0000	0.0000
Faecalibaculum	0.0259	0.0214	0.0196	0.0247	0.2466	0.2935
Turicibacter	0.0391	0.0389	0.0377	0.0539	0.0202	0.0307
Erysipelotrichaceae_uncultured bacterium	0.0006	0.0000	0.0006	0.0004	0.0000	0.0000
Candidatus Saccharimonas	0.0041	0.0043	0.0019	0.0051	0.0000	0.0000
Desulfovibrio	0.0068	0.0068	0.0062	0.0091	0.0000	0.0000
Anaeroplasma	0.0000	0.0000	0.0000	0.0021	0.0000	0.0000
unknown taxa	0.0082	0.0074	0.0054	0.0078	0.0000	0.0000
Mollicutes_ambiguous taxa	0.0000	0.0000	0.0004	0.0000	0.0000	0.0000
Akkermansia	0.0000	0.0010	0.0021	0.0021	0.0282	0.0356

age (1G)								
					1G D7			
M3	M4	M5	M6	M7	M1	M2	M3	M4
0.0123	0.0465	0.0016	0.0307	0.0049	0.0132	0.0076	0.0000	0.0000
0.0000	0.0000	0.0000	0.0000	0.0000	0.0010	0.0000	0.0000	0.0000
0.0000	0.0000	0.0000	0.0000	0.0000	0.0025	0.0006	0.0000	0.0000
0.0165	0.0101	0.0000	0.0126	0.0054	0.0152	0.0128	0.0062	0.0049
0.0000	0.0000	0.0000	0.0000	0.0000	0.0000	0.0000	0.0000	0.0000
0.0268	0.0253	0.0000	0.0224	0.0405	0.0356	0.0556	0.0866	0.0644
0.0091	0.0111	0.0000	0.0029	0.0105	0.0047	0.0064	0.0183	0.0191
0.2665	0.2933	0.1245	0.3390	0.4217	0.2054	0.2171	0.5822	0.4789
0.0000	0.0000	0.0000	0.0000	0.0000	0.0041	0.0031	0.0000	0.0000
0.0008	0.0000	0.0000	0.0000	0.0000	0.0037	0.0012	0.0049	0.0008
0.0000	0.0000	0.0000	0.0000	0.0000	0.0000	0.0000	0.0000	0.0000
0.0000	0.0000	0.0000	0.0000	0.0000	0.0000	0.0000	0.0000	0.0000
0.1745	0.1679	0.2414	0.1301	0.2072	0.2169	0.1965	0.1120	0.1957
0.0000	0.0000	0.0000	0.0000	0.0000	0.0000	0.0047	0.0041	0.0025
0.0062	0.0006	0.0000	0.0021	0.0014	0.0010	0.0000	0.0000	0.0002
0.0000	0.0000	0.0000	0.0000	0.0000	0.0000	0.0000	0.0000	0.0000
0.0000	0.0000	0.0000	0.0000	0.0000	0.0000	0.0000	0.0000	0.0000
0.0000	0.0000	0.0000	0.0000	0.0000	0.0029	0.0004	0.0000	0.0000
0.0397	0.0163	0.0000	0.0049	0.0187	0.0494	0.0416	0.0031	0.0261
0.0000	0.0000	0.0000	0.0000	0.0000	0.0021	0.0010	0.0000	0.0000
0.0000	0.0000	0.0000	0.0000	0.0000	0.0014	0.0023	0.0000	0.0012
0.0037	0.0002	0.3752	0.0706	0.0000	0.0058	0.0091	0.0019	0.0016
0.0019	0.0000	0.0000	0.0000	0.0000	0.0099	0.0060	0.0000	0.0008
0.0564	0.0134	0.0000	0.0000	0.0037	0.0576	0.0171	0.0101	0.0179
0.0216	0.0035	0.0000	0.0051	0.0023	0.0095	0.0105	0.0023	0.0041
0.0142	0.0010	0.0000	0.0000	0.0000	0.0342	0.0881	0.0117	0.0265
0.0000	0.0000	0.0000	0.0000	0.0000	0.0014	0.0080	0.0000	0.0008
0.0014	0.0000	0.0000	0.0000	0.0000	0.0043	0.0016	0.0008	0.0033
0.0000	0.0000	0.0000	0.0000	0.0000	0.0000	0.0000	0.0000	0.0000
0.0000	0.0000	0.0000	0.0000	0.0000	0.0000	0.0000	0.0000	0.0000
0.0288	0.0064	0.0000	0.0000	0.0054	0.0274	0.0323	0.0035	0.0084
0.0000	0.0000	0.0000	0.0000	0.0000	0.0004	0.0004	0.0002	0.0000
0.0103	0.0082	0.0000	0.0021	0.0000	0.0000	0.0000	0.0000	0.0000
0.0054	0.0029	0.0000	0.0010	0.0000	0.0185	0.0099	0.0078	0.0088
0.0249	0.0226	0.0000	0.0000	0.0140	0.0212	0.0218	0.0163	0.0259
0.0014	0.0004	0.0000	0.0000	0.0000	0.0041	0.0021	0.0000	0.0016
0.0033	0.0002	0.0002	0.0000	0.0008	0.0006	0.0000	0.0000	0.0006
0.0004	0.0000	0.0000	0.0000	0.0000	0.0016	0.0021	0.0000	0.0004
0.0023	0.0000	0.0000	0.0000	0.0000	0.0064	0.0060	0.0051	0.0035
0.0000	0.0000	0.0000	0.0000	0.0000	0.0000	0.0000	0.0000	0.0000
0.0000	0.0000	0.0000	0.0000	0.0000	0.0000	0.0029	0.0000	0.0000
0.0000	0.0000	0.0000	0.0000	0.0000	0.0000	0.0000	0.0000	0.0000
0.0000	0.0000	0.0000	0.0000	0.0000	0.0014	0.0008	0.0008	0.0006
0.0000	0.0056	0.0000	0.0002	0.0333	0.0000	0.0000	0.0000	0.0014
0.0000	0.0000	0.0000	0.0000	0.0000	0.0000	0.0000	0.0000	0.0000
0.0000	0.0000	0.0000	0.0000	0.0000	0.0056	0.0008	0.0000	0.0006
0.0000	0.0000	0.0000	0.0000	0.0000	0.0000	0.0000	0.0000	0.0000
0.1227	0.2289	0.0134	0.1276	0.1010	0.1303	0.1295	0.0447	0.0167
0.0486	0.0179	0.0023	0.0146	0.0233	0.0031	0.0031	0.0000	0.0016
0.0000	0.0000	0.0000	0.0004	0.0000	0.0000	0.0002	0.0014	0.0016
0.0000	0.0000	0.0000	0.0000	0.0000	0.0000	0.0000	0.0000	0.0000
0.0000	0.0000	0.0000	0.0000	0.0000	0.0222	0.0074	0.0012	0.0008
0.0000	0.0000	0.0000	0.0000	0.0000	0.0000	0.0000	0.0016	0.0008
0.0000	0.0000	0.0000	0.0000	0.0000	0.0000	0.0000	0.0000	0.0000
0.0000	0.0000	0.0000	0.0000	0.0000	0.0000	0.0000	0.0000	0.0000
0.0527	0.0578	0.1675	0.1877	0.0346	0.0138	0.0257	0.0140	0.0107

			1G D14					
M5	M6	M7	M1	M2	M3	M4	M5	M6
0.0000	0.0000	0.0000	0.0014	0.0006	0.0000	0.0000	0.0000	0.0000
0.0000	0.0000	0.0000	0.0035	0.0000	0.0049	0.0056	0.0000	0.0000
0.0000	0.0000	0.0006	0.0008	0.0000	0.0012	0.0004	0.0000	0.0000
0.0000	0.0000	0.0076	0.0095	0.0142	0.0076	0.0084	0.0000	0.0000
0.0000	0.0000	0.0000	0.0000	0.0000	0.0000	0.0000	0.0000	0.0000
0.0210	0.0342	0.0368	0.0373	0.0313	0.0346	0.0609	0.0385	0.0335
0.0000	0.0000	0.0078	0.0008	0.0014	0.0049	0.0045	0.0035	0.0101
0.1741	0.1533	0.3192	0.1017	0.1056	0.2813	0.3213	0.0988	0.0862
0.0000	0.0000	0.0128	0.0033	0.0027	0.0000	0.0000	0.0000	0.0000
0.0000	0.0000	0.0043	0.0000	0.0000	0.0062	0.0023	0.0000	0.0000
0.0000	0.0000	0.0000	0.0000	0.0000	0.0000	0.0000	0.0000	0.0000
0.0000	0.0000	0.0000	0.0000	0.0000	0.0000	0.0000	0.0000	0.0000
0.0099	0.0086	0.0119	0.5701	0.4089	0.0801	0.2412	0.0430	0.0554
0.0000	0.0000	0.0000	0.0051	0.0084	0.0010	0.0033	0.0000	0.0000
0.0000	0.0000	0.0000	0.0000	0.0000	0.0000	0.0000	0.0000	0.0000
0.0000	0.0000	0.0000	0.0000	0.0000	0.0000	0.0000	0.0000	0.0000
0.0010	0.0051	0.0002	0.0000	0.0006	0.0000	0.0000	0.0035	0.0016
0.0000	0.0000	0.0000	0.0000	0.0000	0.0008	0.0000	0.0000	0.0000
0.0000	0.0014	0.0823	0.0206	0.0346	0.0757	0.0307	0.0029	0.0047
0.0000	0.0000	0.0000	0.0010	0.0054	0.0142	0.0000	0.0000	0.0000
0.0000	0.0000	0.0021	0.0025	0.0031	0.0027	0.0021	0.0000	0.0000
0.3492	0.3645	0.0352	0.0093	0.0091	0.0095	0.0154	0.3937	0.4056
0.0000	0.0000	0.0000	0.0010	0.0031	0.0025	0.0021	0.0000	0.0000
0.0000	0.0000	0.0354	0.0144	0.0307	0.0373	0.0113	0.0000	0.0000
0.0000	0.0000	0.0000	0.0033	0.0037	0.0091	0.0047	0.0000	0.0000
0.0000	0.0000	0.0519	0.0451	0.0517	0.1434	0.0920	0.0000	0.0000
0.0000	0.0000	0.0019	0.0058	0.0134	0.0282	0.0158	0.0000	0.0000
0.0000	0.0000	0.0241	0.0021	0.0049	0.0128	0.0031	0.0000	0.0000
0.0000	0.0002	0.0000	0.0105	0.0070	0.0210	0.0072	0.0142	0.0093
0.0000	0.0000	0.0000	0.0000	0.0000	0.0000	0.0000	0.0000	0.0000
0.0088	0.0000	0.0329	0.0095	0.0181	0.0272	0.0119	0.0000	0.0000
0.0000	0.0000	0.0010	0.0008	0.0008	0.0023	0.0006	0.0000	0.0000
0.0000	0.0000	0.0000	0.0000	0.0000	0.0000	0.0000	0.0000	0.0000
0.0031	0.0062	0.0395	0.0099	0.0189	0.0216	0.0158	0.0014	0.0000
0.0000	0.0000	0.0031	0.0000	0.0049	0.0000	0.0027	0.0000	0.0000
0.0000	0.0000	0.0031	0.0033	0.0039	0.0084	0.0016	0.0000	0.0000
0.0010	0.0000	0.0000	0.0000	0.0000	0.0014	0.0000	0.0035	0.0031
0.0002	0.0000	0.0058	0.0014	0.0037	0.0064	0.0035	0.0000	0.0000
0.0088	0.0043	0.0117	0.0027	0.0066	0.0204	0.0019	0.0019	0.0039
0.0000	0.0000	0.0000	0.0000	0.0000	0.0014	0.0000	0.0000	0.0000
0.0000	0.0000	0.0035	0.0000	0.0093	0.0095	0.0054	0.0000	0.0000
0.0000	0.0000	0.0000	0.0000	0.0014	0.0062	0.0000	0.0000	0.0000
0.0000	0.0000	0.0006	0.0004	0.0010	0.0002	0.0010	0.0000	0.0000
0.0000	0.0000	0.0002	0.0000	0.0000	0.0010	0.0000	0.0000	0.0000
0.0000	0.0000	0.0000	0.0012	0.0010	0.0008	0.0000	0.0000	0.0000
0.0000	0.0000	0.0029	0.0000	0.0000	0.0008	0.0000	0.0000	0.0000
0.0000	0.0000	0.0000	0.0000	0.0000	0.0000	0.0000	0.0000	0.0000
0.0014	0.0008	0.0045	0.0897	0.1412	0.0101	0.0673	0.0014	0.0000
0.0000	0.0000	0.0000	0.0000	0.0016	0.0000	0.0051	0.0000	0.0000
0.0000	0.0000	0.0002	0.0012	0.0000	0.0019	0.0012	0.0000	0.0000
0.0000	0.0000	0.0000	0.0000	0.0000	0.0000	0.0000	0.0000	0.0000
0.0000	0.0000	0.0105	0.0082	0.0043	0.0099	0.0093	0.0000	0.0000
0.0000	0.0008	0.0010	0.0000	0.0012	0.0047	0.0080	0.0000	0.0000
0.0000	0.0000	0.0000	0.0000	0.0004	0.0000	0.0000	0.0000	0.0000
0.0000	0.0000	0.0000	0.0000	0.0000	0.0000	0.0000	0.0000	0.0000
0.2496	0.2212	0.1725	0.0000	0.0000	0.0000	0.0014	0.1640	0.1718

	1G D21							1G D28
M7	M1	M2	M3	M4	M5	M6	M7	M1
0.0000	0.0000	0.0000	0.0000	0.0000	0.0000	0.0002	0.0000	0.0008
0.0023	0.0058	0.0010	0.0021	0.0016	0.0000	0.0002	0.0016	0.0029
0.0010	0.0000	0.0000	0.0000	0.0010	0.0000	0.0000	0.0000	0.0006
0.0119	0.0099	0.0064	0.0134	0.0105	0.0025	0.0000	0.0033	0.0097
0.0000	0.0000	0.0000	0.0000	0.0000	0.0000	0.0000	0.0000	0.0000
0.0494	0.0414	0.0681	0.0599	0.0650	0.0354	0.0292	0.0570	0.0597
0.0033	0.0016	0.0039	0.0072	0.0095	0.0245	0.0148	0.0047	0.0058
0.2501	0.1422	0.2472	0.3390	0.3546	0.0801	0.1048	0.3159	0.2367
0.0084	0.0027	0.0105	0.0000	0.0000	0.0000	0.0000	0.0202	0.0109
0.0002	0.0002	0.0004	0.0025	0.0021	0.0000	0.0000	0.0016	0.0012
0.0000	0.0000	0.0000	0.0000	0.0000	0.0000	0.0000	0.0000	0.0000
0.0008	0.0000	0.0000	0.0000	0.0000	0.0000	0.0002	0.0000	0.0000
0.2214	0.6228	0.3231	0.3427	0.2824	0.1025	0.0751	0.4093	0.2622
0.0000	0.0056	0.0066	0.0080	0.0051	0.0000	0.0000	0.0000	0.0000
0.0000	0.0000	0.0000	0.0000	0.0000	0.0000	0.0000	0.0000	0.0000
0.0000	0.0000	0.0000	0.0000	0.0000	0.0000	0.0000	0.0000	0.0000
0.0023	0.0000	0.0008	0.0000	0.0000	0.0000	0.0010	0.0000	0.0000
0.0027	0.0000	0.0000	0.0000	0.0000	0.0000	0.0000	0.0000	0.0000
0.0811	0.0088	0.0471	0.0142	0.0058	0.0019	0.0023	0.0119	0.0121
0.0000	0.0000	0.0054	0.0058	0.0000	0.0000	0.0000	0.0000	0.0000
0.0000	0.0000	0.0047	0.0000	0.0000	0.0000	0.0000	0.0008	0.0016
0.0097	0.0029	0.0093	0.0000	0.0023	0.3248	0.3562	0.0082	0.0023
0.0000	0.0000	0.0029	0.0000	0.0000	0.0000	0.0000	0.0010	0.0000
0.0480	0.0113	0.0140	0.0119	0.0076	0.0000	0.0000	0.0091	0.0062
0.0000	0.0002	0.0049	0.0014	0.0000	0.0000	0.0000	0.0000	0.0000
0.0486	0.0109	0.0780	0.0200	0.0265	0.0000	0.0000	0.0161	0.0181
0.0060	0.0012	0.0076	0.0016	0.0000	0.0000	0.0000	0.0008	0.0021
0.0272	0.0062	0.0039	0.0037	0.0031	0.0000	0.0000	0.0054	0.0051
0.0128	0.0008	0.0054	0.0000	0.0023	0.0012	0.0025	0.0029	0.0041
0.0000	0.0000	0.0000	0.0000	0.0000	0.0000	0.0000	0.0000	0.0000
0.0525	0.0004	0.0103	0.0010	0.0019	0.0000	0.0000	0.0041	0.0056
0.0033	0.0000	0.0008	0.0002	0.0000	0.0000	0.0000	0.0006	0.0000
0.0000	0.0000	0.0000	0.0000	0.0000	0.0000	0.0000	0.0000	0.0000
0.0210	0.0037	0.0161	0.0021	0.0033	0.0002	0.0000	0.0066	0.0064
0.0000	0.0000	0.0045	0.0000	0.0047	0.0000	0.0000	0.0000	0.0084
0.0041	0.0000	0.0014	0.0000	0.0000	0.0000	0.0000	0.0000	0.0000
0.0010	0.0000	0.0000	0.0004	0.0000	0.0000	0.0000	0.0021	0.0002
0.0010	0.0000	0.0029	0.0006	0.0010	0.0000	0.0000	0.0000	0.0000
0.0113	0.0000	0.0031	0.0016	0.0000	0.0023	0.0021	0.0014	0.0006
0.0025	0.0000	0.0000	0.0000	0.0000	0.0000	0.0000	0.0000	0.0000
0.0041	0.0000	0.0039	0.0019	0.0000	0.0000	0.0000	0.0019	0.0016
0.0000	0.0000	0.0010	0.0002	0.0000	0.0000	0.0000	0.0008	0.0000
0.0019	0.0004	0.0019	0.0006	0.0016	0.0000	0.0000	0.0000	0.0021
0.0049	0.0000	0.0000	0.0004	0.0000	0.0000	0.0000	0.0045	0.0000
0.0000	0.0041	0.0027	0.0047	0.0060	0.0000	0.0000	0.0000	0.0056
0.0008	0.0000	0.0004	0.0000	0.0000	0.0000	0.0000	0.0012	0.0000
0.0000	0.0000	0.0000	0.0000	0.0000	0.0000	0.0000	0.0000	0.0000
0.0323	0.0895	0.0272	0.1066	0.1663	0.0080	0.0039	0.0228	0.2700
0.0051	0.0000	0.0000	0.0004	0.0019	0.0000	0.0000	0.0000	0.0000
0.0016	0.0008	0.0012	0.0008	0.0010	0.0000	0.0000	0.0021	0.0000
0.0000	0.0000	0.0016	0.0000	0.0000	0.0000	0.0000	0.0000	0.0000
0.0167	0.0058	0.0142	0.0101	0.0082	0.0000	0.0031	0.0039	0.0066
0.0014	0.0000	0.0021	0.0004	0.0029	0.0000	0.0000	0.0019	0.0006
0.0000	0.0000	0.0004	0.0000	0.0000	0.0000	0.0000	0.0000	0.0000
0.0000	0.0000	0.0000	0.0000	0.0000	0.0000	0.0000	0.0000	0.0000
0.0152	0.0000	0.0000	0.0002	0.0000	0.2342	0.2159	0.0325	0.0008

						1G D35		
**M2**	**M3**	**M4**	**M5**	**M6**	**M7**	**M1**	**M2**	**M3**
0.0012	0.0000	0.0000	0.0000	0.0000	0.0000	0.0078	0.0039	0.0000
0.0043	0.0072	0.0058	0.0000	0.0000	0.0025	0.0000	0.0010	0.0000
0.0000	0.0006	0.0010	0.0000	0.0000	0.0000	0.0000	0.0000	0.0012
0.0039	0.0084	0.0132	0.0097	0.0000	0.0047	0.0179	0.0208	0.0198
0.0000	0.0000	0.0000	0.0000	0.0000	0.0000	0.0000	0.0000	0.0000
0.0574	0.0774	0.0741	0.0414	0.0303	0.0547	0.0377	0.0445	0.0484
0.0064	0.0086	0.0039	0.0218	0.0265	0.0051	0.0043	0.0027	0.0115
0.1854	0.3163	0.3239	0.3217	0.2048	0.2982	0.1681	0.1476	0.2445
0.0062	0.0000	0.0000	0.0140	0.0037	0.0138	0.0082	0.0056	0.0000
0.0006	0.0025	0.0031	0.0029	0.0008	0.0012	0.0000	0.0019	0.0105
0.0000	0.0000	0.0000	0.0000	0.0000	0.0000	0.0000	0.0000	0.0000
0.0000	0.0000	0.0000	0.0008	0.0064	0.0004	0.0000	0.0000	0.0000
0.3289	0.2513	0.2929	0.1772	0.0967	0.3435	0.5092	0.3031	0.3704
0.0021	0.0016	0.0066	0.0000	0.0000	0.0000	0.0000	0.0041	0.0142
0.0002	0.0000	0.0000	0.0000	0.0000	0.0000	0.0000	0.0000	0.0000
0.0000	0.0006	0.0000	0.0000	0.0000	0.0000	0.0000	0.0000	0.0000
0.0000	0.0021	0.0014	0.0016	0.0004	0.0000	0.0000	0.0000	0.0000
0.0000	0.0012	0.0002	0.0000	0.0000	0.0000	0.0000	0.0012	0.0000
0.0097	0.0535	0.0296	0.0070	0.0021	0.0012	0.0156	0.0753	0.0638
0.0033	0.0152	0.0121	0.0000	0.0000	0.0000	0.0016	0.0082	0.0099
0.0000	0.0000	0.0000	0.0000	0.0000	0.0000	0.0004	0.0072	0.0000
0.0027	0.0012	0.0000	0.0578	0.2764	0.0043	0.0086	0.0136	0.0039
0.0006	0.0023	0.0012	0.0000	0.0000	0.0000	0.0010	0.0109	0.0000
0.0074	0.0570	0.0216	0.0049	0.0000	0.0076	0.0082	0.0403	0.0202
0.0021	0.0045	0.0031	0.0000	0.0000	0.0000	0.0012	0.0078	0.0021
0.0245	0.0305	0.0794	0.0000	0.0000	0.0054	0.0210	0.0381	0.0231
0.0014	0.0000	0.0000	0.0000	0.0000	0.0012	0.0021	0.0051	0.0000
0.0033	0.0185	0.0066	0.0000	0.0000	0.0008	0.0000	0.0146	0.0000
0.0019	0.0101	0.0074	0.0103	0.0105	0.0008	0.0054	0.0066	0.0128
0.0000	0.0000	0.0000	0.0000	0.0000	0.0000	0.0000	0.0000	0.0000
0.0019	0.0072	0.0047	0.0047	0.0000	0.0021	0.0000	0.0263	0.0021
0.0004	0.0000	0.0000	0.0000	0.0000	0.0000	0.0000	0.0012	0.0000
0.0000	0.0000	0.0000	0.0000	0.0000	0.0000	0.0000	0.0000	0.0000
0.0103	0.0078	0.0099	0.0025	0.0002	0.0019	0.0126	0.0148	0.0177
0.0000	0.0000	0.0000	0.0000	0.0000	0.0000	0.0010	0.0041	0.0000
0.0000	0.0016	0.0010	0.0000	0.0000	0.0006	0.0016	0.0051	0.0014
0.0000	0.0012	0.0000	0.0016	0.0006	0.0000	0.0004	0.0025	0.0000
0.0010	0.0027	0.0004	0.0008	0.0000	0.0002	0.0000	0.0037	0.0000
0.0000	0.0103	0.0010	0.0047	0.0023	0.0004	0.0014	0.0060	0.0012
0.0000	0.0000	0.0000	0.0000	0.0000	0.0000	0.0000	0.0000	0.0000
0.0012	0.0014	0.0023	0.0000	0.0000	0.0000	0.0000	0.0047	0.0064
0.0000	0.0012	0.0010	0.0000	0.0000	0.0000	0.0000	0.0021	0.0035
0.0012	0.0019	0.0006	0.0000	0.0000	0.0002	0.0031	0.0031	0.0014
0.0004	0.0000	0.0000	0.0000	0.0000	0.0025	0.0010	0.0000	0.0000
0.0041	0.0008	0.0056	0.0000	0.0000	0.0000	0.0051	0.0060	0.0088
0.0000	0.0000	0.0000	0.0000	0.0000	0.0006	0.0000	0.0019	0.0000
0.0000	0.0000	0.0000	0.0000	0.0000	0.0000	0.0000	0.0000	0.0000
0.2830	0.0119	0.0288	0.0459	0.0146	0.1039	0.1150	0.0811	0.0451
0.0000	0.0000	0.0000	0.0000	0.0000	0.0000	0.0000	0.0000	0.0000
0.0000	0.0010	0.0000	0.0010	0.0000	0.0006	0.0008	0.0014	0.0012
0.0000	0.0000	0.0000	0.0000	0.0000	0.0000	0.0000	0.0000	0.0000
0.0004	0.0292	0.0105	0.0021	0.0002	0.0012	0.0064	0.0097	0.0062
0.0033	0.0039	0.0043	0.0000	0.0000	0.0006	0.0000	0.0064	0.0091
0.0000	0.0000	0.0000	0.0000	0.0000	0.0000	0.0000	0.0000	0.0000
0.0000	0.0000	0.0000	0.0000	0.0000	0.0000	0.0000	0.0000	0.0000
0.0000	0.0000	0.0000	0.1955	0.2210	0.0965	0.0010	0.0000	0.0000

				1G D42				
M4	M5	M6	M7	M1	M2	M3	M4	M5
0.0000	0.0000	0.0000	0.0000	0.0165	0.0099	0.0000	0.0004	0.0000
0.0000	0.0000	0.0000	0.0027	0.0000	0.0000	0.0000	0.0010	0.0000
0.0021	0.0023	0.0008	0.0037	0.0010	0.0021	0.0002	0.0016	0.0014
0.0175	0.0206	0.0179	0.0241	0.0311	0.0146	0.0169	0.0179	0.0181
0.0000	0.0000	0.0000	0.0000	0.0000	0.0000	0.0000	0.0000	0.0000
0.0449	0.0315	0.0412	0.0447	0.0389	0.0233	0.0387	0.0358	0.0368
0.0107	0.0093	0.0049	0.0066	0.0056	0.0074	0.0062	0.0068	0.0084
0.2354	0.2136	0.2229	0.2161	0.1788	0.1451	0.1700	0.2241	0.1743
0.0000	0.0109	0.0039	0.0058	0.0058	0.0041	0.0000	0.0000	0.0099
0.0097	0.0054	0.0033	0.0039	0.0008	0.0027	0.0082	0.0033	0.0019
0.0000	0.0000	0.0000	0.0000	0.0000	0.0000	0.0000	0.0000	0.0000
0.0000	0.0004	0.0000	0.0000	0.0000	0.0000	0.0000	0.0000	0.0062
0.4065	0.1126	0.1959	0.1850	0.3458	0.1844	0.2239	0.4524	0.2478
0.0080	0.0000	0.0000	0.0000	0.0000	0.0010	0.0088	0.0000	0.0000
0.0000	0.0000	0.0000	0.0000	0.0103	0.0051	0.0000	0.0000	0.0000
0.0000	0.0006	0.0000	0.0000	0.0000	0.0000	0.0000	0.0000	0.0000
0.0000	0.0054	0.0058	0.0043	0.0000	0.0031	0.0002	0.0006	0.0025
0.0000	0.0047	0.0025	0.0037	0.0000	0.0027	0.0019	0.0000	0.0025
0.0261	0.1519	0.1039	0.1029	0.0101	0.1192	0.0953	0.0220	0.1395
0.0107	0.0000	0.0000	0.0000	0.0045	0.0115	0.0123	0.0117	0.0000
0.0021	0.0047	0.0189	0.0111	0.0019	0.0066	0.0041	0.0000	0.0016
0.0023	0.0025	0.0084	0.0128	0.0062	0.0146	0.0093	0.0021	0.0062
0.0000	0.0033	0.0158	0.0043	0.0008	0.0142	0.0029	0.0019	0.0006
0.0140	0.0263	0.0311	0.0370	0.0043	0.0389	0.0181	0.0113	0.0167
0.0045	0.0027	0.0056	0.0066	0.0010	0.0169	0.0091	0.0023	0.0097
0.0305	0.0667	0.0578	0.0465	0.0282	0.0677	0.0770	0.0368	0.0494
0.0000	0.0000	0.0025	0.0095	0.0074	0.0043	0.0000	0.0000	0.0134
0.0068	0.0196	0.0148	0.0177	0.0051	0.0136	0.0158	0.0084	0.0126
0.0068	0.0239	0.0080	0.0231	0.0101	0.0255	0.0181	0.0115	0.0091
0.0000	0.0000	0.0000	0.0000	0.0000	0.0000	0.0000	0.0000	0.0000
0.0035	0.0438	0.0366	0.0490	0.0068	0.0459	0.0239	0.0043	0.0115
0.0000	0.0016	0.0000	0.0000	0.0004	0.0004	0.0010	0.0002	0.0014
0.0000	0.0000	0.0000	0.0000	0.0000	0.0000	0.0000	0.0000	0.0000
0.0134	0.0241	0.0198	0.0163	0.0093	0.0156	0.0239	0.0113	0.0189
0.0000	0.0000	0.0095	0.0000	0.0066	0.0091	0.0000	0.0000	0.0000
0.0019	0.0019	0.0086	0.0045	0.0004	0.0080	0.0056	0.0031	0.0117
0.0000	0.0045	0.0043	0.0027	0.0000	0.0006	0.0016	0.0000	0.0039
0.0008	0.0049	0.0047	0.0027	0.0000	0.0029	0.0121	0.0000	0.0078
0.0056	0.0344	0.0128	0.0037	0.0014	0.0039	0.0274	0.0016	0.0235
0.0000	0.0000	0.0000	0.0027	0.0000	0.0010	0.0045	0.0006	0.0000
0.0043	0.0054	0.0000	0.0029	0.0010	0.0019	0.0093	0.0016	0.0095
0.0021	0.0064	0.0021	0.0029	0.0000	0.0008	0.0126	0.0000	0.0076
0.0000	0.0014	0.0023	0.0074	0.0023	0.0033	0.0078	0.0021	0.0010
0.0000	0.0080	0.0072	0.0091	0.0035	0.0000	0.0000	0.0000	0.0064
0.0068	0.0000	0.0000	0.0000	0.0041	0.0086	0.0043	0.0033	0.0000
0.0000	0.0115	0.0171	0.0084	0.0000	0.0023	0.0000	0.0000	0.0000
0.0000	0.0000	0.0000	0.0000	0.0000	0.0000	0.0000	0.0000	0.0000
0.0652	0.0072	0.0224	0.0167	0.1803	0.0860	0.0350	0.0669	0.0270
0.0000	0.0000	0.0000	0.0000	0.0091	0.0047	0.0000	0.0000	0.0012
0.0016	0.0035	0.0039	0.0012	0.0016	0.0000	0.0002	0.0014	0.0023
0.0000	0.0000	0.0000	0.0000	0.0000	0.0000	0.0000	0.0000	0.0000
0.0113	0.0097	0.0161	0.0280	0.0082	0.0117	0.0093	0.0109	0.0074
0.0111	0.0000	0.0000	0.0041	0.0000	0.0000	0.0103	0.0078	0.0068
0.0000	0.0000	0.0000	0.0000	0.0002	0.0000	0.0000	0.0000	0.0000
0.0000	0.0000	0.0000	0.0000	0.0000	0.0000	0.0000	0.0002	0.0000
0.0000	0.0179	0.0051	0.0012	0.0000	0.0000	0.0000	0.0000	0.0033

		1G D49						
M6	M7	M1	M2	M3	M4	M5	M6	M7
0.0000	0.0000	0.0095	0.0076	0.0023	0.0037	0.0000	0.0000	0.0000
0.0000	0.0000	0.0000	0.0000	0.0000	0.0000	0.0000	0.0014	0.0000
0.0008	0.0012	0.0019	0.0008	0.0000	0.0000	0.0006	0.0000	0.0000
0.0148	0.0228	0.0222	0.0218	0.0171	0.0144	0.0128	0.0068	0.0111
0.0000	0.0000	0.0000	0.0000	0.0000	0.0000	0.0000	0.0000	0.0000
0.0274	0.0338	0.0445	0.0428	0.0447	0.0424	0.0247	0.0284	0.0385
0.0072	0.0082	0.0064	0.0088	0.0086	0.0105	0.0091	0.0113	0.0049
0.1930	0.1778	0.2282	0.1902	0.2029	0.2272	0.1875	0.1780	0.2270
0.0082	0.0097	0.0082	0.0080	0.0000	0.0000	0.0062	0.0082	0.0097
0.0041	0.0010	0.0008	0.0033	0.0078	0.0095	0.0033	0.0060	0.0027
0.0000	0.0000	0.0000	0.0000	0.0000	0.0000	0.0000	0.0000	0.0000
0.0144	0.0086	0.0000	0.0000	0.0000	0.0000	0.0000	0.0031	0.0000
0.4824	0.4970	0.3346	0.2177	0.3651	0.3698	0.2484	0.3336	0.4435
0.0000	0.0000	0.0000	0.0012	0.0163	0.0000	0.0000	0.0000	0.0000
0.0000	0.0000	0.0033	0.0074	0.0000	0.0000	0.0000	0.0000	0.0000
0.0000	0.0000	0.0000	0.0000	0.0006	0.0000	0.0000	0.0000	0.0000
0.0000	0.0000	0.0000	0.0023	0.0021	0.0000	0.0014	0.0000	0.0000
0.0000	0.0000	0.0000	0.0025	0.0012	0.0000	0.0023	0.0016	0.0000
0.0300	0.0177	0.0171	0.0782	0.0595	0.0463	0.1362	0.0716	0.0136
0.0000	0.0000	0.0056	0.0117	0.0091	0.0043	0.0000	0.0000	0.0000
0.0000	0.0000	0.0058	0.0093	0.0010	0.0037	0.0041	0.0060	0.0006
0.0035	0.0037	0.0078	0.0082	0.0074	0.0113	0.0070	0.0076	0.0056
0.0000	0.0000	0.0025	0.0103	0.0000	0.0000	0.0010	0.0045	0.0000
0.0138	0.0064	0.0270	0.0278	0.0121	0.0027	0.0146	0.0311	0.0062
0.0000	0.0010	0.0062	0.0091	0.0035	0.0047	0.0136	0.0025	0.0000
0.0231	0.0274	0.0375	0.0539	0.0576	0.0593	0.0504	0.0463	0.0300
0.0078	0.0019	0.0093	0.0088	0.0000	0.0000	0.0191	0.0070	0.0039
0.0039	0.0047	0.0072	0.0132	0.0000	0.0043	0.0062	0.0113	0.0016
0.0008	0.0068	0.0167	0.0093	0.0179	0.0132	0.0128	0.0101	0.0021
0.0000	0.0000	0.0000	0.0000	0.0000	0.0000	0.0000	0.0000	0.0000
0.0088	0.0111	0.0144	0.0210	0.0076	0.0045	0.0204	0.0263	0.0093
0.0006	0.0006	0.0000	0.0002	0.0008	0.0000	0.0019	0.0021	0.0000
0.0000	0.0000	0.0000	0.0000	0.0000	0.0000	0.0000	0.0000	0.0000
0.0154	0.0066	0.0099	0.0173	0.0167	0.0146	0.0191	0.0208	0.0084
0.0016	0.0037	0.0000	0.0000	0.0000	0.0000	0.0093	0.0043	0.0064
0.0025	0.0014	0.0012	0.0058	0.0029	0.0027	0.0132	0.0078	0.0033
0.0000	0.0000	0.0000	0.0010	0.0000	0.0000	0.0041	0.0012	0.0008
0.0035	0.0000	0.0033	0.0056	0.0054	0.0047	0.0177	0.0128	0.0019
0.0029	0.0012	0.0016	0.0056	0.0027	0.0021	0.0233	0.0097	0.0029
0.0000	0.0000	0.0000	0.0029	0.0027	0.0000	0.0002	0.0000	0.0000
0.0074	0.0033	0.0027	0.0019	0.0047	0.0054	0.0086	0.0051	0.0039
0.0010	0.0000	0.0000	0.0035	0.0012	0.0066	0.0107	0.0012	0.0039
0.0006	0.0019	0.0043	0.0045	0.0004	0.0031	0.0043	0.0019	0.0023
0.0113	0.0084	0.0029	0.0016	0.0000	0.0008	0.0021	0.0049	0.0078
0.0000	0.0000	0.0043	0.0121	0.0103	0.0062	0.0000	0.0000	0.0000
0.0025	0.0019	0.0000	0.0029	0.0000	0.0000	0.0023	0.0072	0.0008
0.0000	0.0000	0.0000	0.0000	0.0000	0.0000	0.0000	0.0000	0.0000
0.0459	0.0726	0.0846	0.0412	0.0130	0.0272	0.0111	0.0393	0.0683
0.0000	0.0000	0.0086	0.0045	0.0107	0.0107	0.0054	0.0123	0.0206
0.0016	0.0025	0.0012	0.0010	0.0037	0.0014	0.0019	0.0019	0.0023
0.0000	0.0000	0.0025	0.0004	0.0000	0.0000	0.0000	0.0000	0.0000
0.0049	0.0080	0.0060	0.0068	0.0103	0.0088	0.0084	0.0080	0.0051
0.0023	0.0043	0.0008	0.0037	0.0128	0.0101	0.0070	0.0051	0.0086
0.0000	0.0000	0.0000	0.0000	0.0000	0.0000	0.0000	0.0000	0.0000
0.0000	0.0000	0.0000	0.0000	0.0000	0.0000	0.0000	0.0000	0.0000
0.0000	0.0000	0.0000	0.0000	0.0000	0.0000	0.0008	0.0008	0.0010

1G D56							1G D63	
M1	M2	M3	M4	M5	M6	M7	M1	M2
0.0012	0.0049	0.0016	0.0021	0.0000	0.0000	0.0000	0.0305	0.0121
0.0000	0.0000	0.0000	0.0000	0.0016	0.0016	0.0000	0.0000	0.0000
0.0029	0.0000	0.0037	0.0027	0.0000	0.0000	0.0000	0.0012	0.0029
0.0274	0.0138	0.0235	0.0146	0.0191	0.0239	0.0325	0.0111	0.0196
0.0000	0.0000	0.0000	0.0000	0.0000	0.0000	0.0000	0.0000	0.0000
0.0395	0.0321	0.0506	0.0661	0.0373	0.0488	0.0373	0.0410	0.0368
0.0070	0.0099	0.0066	0.0117	0.0093	0.0080	0.0074	0.0107	0.0072
0.1597	0.2025	0.1776	0.2361	0.2050	0.2511	0.2340	0.2179	0.2190
0.0060	0.0074	0.0000	0.0000	0.0082	0.0107	0.0088	0.0093	0.0074
0.0016	0.0031	0.0084	0.0078	0.0037	0.0115	0.0054	0.0019	0.0010
0.0000	0.0000	0.0000	0.0000	0.0000	0.0000	0.0000	0.0000	0.0000
0.0000	0.0000	0.0000	0.0000	0.0099	0.0535	0.0134	0.0000	0.0000
0.1432	0.3431	0.2914	0.3906	0.1638	0.2173	0.1255	0.3832	0.2011
0.0000	0.0072	0.0107	0.0000	0.0000	0.0000	0.0000	0.0000	0.0006
0.0000	0.0000	0.0000	0.0000	0.0000	0.0000	0.0000	0.0002	0.0000
0.0000	0.0000	0.0000	0.0000	0.0000	0.0000	0.0006	0.0002	0.0000
0.0037	0.0000	0.0014	0.0000	0.0047	0.0000	0.0031	0.0000	0.0000
0.0027	0.0000	0.0000	0.0000	0.0029	0.0000	0.0019	0.0000	0.0000
0.0963	0.0218	0.0685	0.0107	0.1072	0.0329	0.0936	0.0043	0.0537
0.0119	0.0156	0.0105	0.0099	0.0000	0.0000	0.0000	0.0000	0.0105
0.0191	0.0014	0.0027	0.0000	0.0039	0.0000	0.0045	0.0029	0.0121
0.0193	0.0086	0.0109	0.0049	0.0144	0.0051	0.0167	0.0025	0.0138
0.0136	0.0019	0.0000	0.0000	0.0000	0.0008	0.0029	0.0000	0.0025
0.0405	0.0189	0.0161	0.0000	0.0321	0.0062	0.0255	0.0056	0.0298
0.0111	0.0037	0.0054	0.0000	0.0062	0.0025	0.0049	0.0000	0.0025
0.0502	0.0265	0.0613	0.0187	0.0595	0.0338	0.0724	0.0193	0.0582
0.0175	0.0146	0.0000	0.0000	0.0056	0.0041	0.0051	0.0023	0.0084
0.0105	0.0101	0.0051	0.0000	0.0113	0.0060	0.0097	0.0060	0.0086
0.0243	0.0138	0.0189	0.0066	0.0163	0.0078	0.0216	0.0060	0.0226
0.0060	0.0000	0.0000	0.0000	0.0000	0.0000	0.0000	0.0000	0.0000
0.0375	0.0138	0.0091	0.0000	0.0158	0.0183	0.0430	0.0035	0.0216
0.0000	0.0010	0.0012	0.0000	0.0025	0.0000	0.0031	0.0000	0.0000
0.0000	0.0000	0.0000	0.0000	0.0000	0.0000	0.0000	0.0000	0.0000
0.0253	0.0103	0.0206	0.0068	0.0356	0.0228	0.0263	0.0043	0.0212
0.0016	0.0012	0.0000	0.0000	0.0060	0.0066	0.0076	0.0000	0.0008
0.0066	0.0027	0.0070	0.0021	0.0121	0.0051	0.0103	0.0000	0.0029
0.0014	0.0000	0.0000	0.0000	0.0033	0.0000	0.0012	0.0000	0.0021
0.0023	0.0016	0.0070	0.0016	0.0064	0.0021	0.0099	0.0000	0.0072
0.0193	0.0002	0.0054	0.0027	0.0373	0.0039	0.0078	0.0000	0.0082
0.0047	0.0000	0.0037	0.0000	0.0074	0.0000	0.0000	0.0000	0.0000
0.0039	0.0025	0.0047	0.0025	0.0121	0.0035	0.0099	0.0000	0.0072
0.0029	0.0008	0.0033	0.0000	0.0097	0.0039	0.0033	0.0000	0.0029
0.0019	0.0033	0.0008	0.0021	0.0016	0.0029	0.0035	0.0021	0.0023
0.0027	0.0000	0.0008	0.0051	0.0033	0.0027	0.0012	0.0000	0.0008
0.0049	0.0152	0.0014	0.0185	0.0000	0.0000	0.0000	0.0029	0.0060
0.0025	0.0000	0.0000	0.0000	0.0076	0.0031	0.0047	0.0000	0.0021
0.0000	0.0000	0.0000	0.0000	0.0000	0.0000	0.0000	0.0000	0.0000
0.0424	0.1076	0.0276	0.0451	0.0158	0.0681	0.0342	0.1527	0.0677
0.0080	0.0200	0.0743	0.0912	0.0103	0.0455	0.0154	0.0307	0.0416
0.0006	0.0016	0.0000	0.0014	0.0035	0.0023	0.0021	0.0006	0.0016
0.0006	0.0000	0.0000	0.0000	0.0000	0.0000	0.0000	0.0000	0.0000
0.0206	0.0070	0.0078	0.0025	0.0150	0.0056	0.0115	0.0054	0.0091
0.0068	0.0016	0.0119	0.0031	0.0123	0.0171	0.0088	0.0000	0.0023
0.0000	0.0000	0.0000	0.0000	0.0000	0.0000	0.0000	0.0000	0.0000
0.0000	0.0000	0.0000	0.0008	0.0000	0.0000	0.0000	0.0000	0.0000
0.0000	0.0000	0.0000	0.0000	0.0000	0.0000	0.0000	0.0012	0.0000

					1G D70			
M3	M4	M5	M6	M7	M1	M2	M3	M4
0.0021	0.0060	0.0000	0.0000	0.0000	0.0010	0.0019	0.0008	0.0074
0.0000	0.0000	0.0000	0.0000	0.0000	0.0000	0.0000	0.0016	0.0000
0.0035	0.0031	0.0010	0.0000	0.0012	0.0000	0.0014	0.0033	0.0045
0.0270	0.0154	0.0158	0.0187	0.0171	0.0231	0.0148	0.0208	0.0128
0.0000	0.0000	0.0000	0.0000	0.0000	0.0000	0.0000	0.0000	0.0000
0.0442	0.0554	0.0276	0.0329	0.0276	0.0515	0.0473	0.0393	0.0480
0.0109	0.0088	0.0084	0.0088	0.0080	0.0121	0.0088	0.0084	0.0115
0.1807	0.2498	0.1611	0.2190	0.1706	0.2801	0.2194	0.1739	0.2416
0.0000	0.0000	0.0054	0.0095	0.0049	0.0115	0.0078	0.0000	0.0000
0.0167	0.0080	0.0004	0.0023	0.0010	0.0054	0.0054	0.0072	0.0062
0.0000	0.0000	0.0000	0.0000	0.0000	0.0000	0.0000	0.0000	0.0000
0.0000	0.0000	0.0000	0.0029	0.0000	0.0000	0.0000	0.0000	0.0000
0.3013	0.4355	0.2132	0.2068	0.4130	0.2951	0.1702	0.1091	0.3900
0.0029	0.0000	0.0000	0.0000	0.0000	0.0000	0.0000	0.0000	0.0000
0.0000	0.0000	0.0000	0.0000	0.0000	0.0000	0.0000	0.0000	0.0000
0.0000	0.0000	0.0000	0.0000	0.0000	0.0000	0.0000	0.0006	0.0000
0.0029	0.0000	0.0010	0.0004	0.0008	0.0000	0.0004	0.0014	0.0000
0.0014	0.0000	0.0019	0.0002	0.0012	0.0000	0.0000	0.0031	0.0000
0.0809	0.0183	0.0850	0.0303	0.0113	0.0261	0.0636	0.1325	0.0307
0.0103	0.0097	0.0000	0.0000	0.0000	0.0082	0.0165	0.0119	0.0101
0.0000	0.0000	0.0039	0.0064	0.0039	0.0037	0.0132	0.0043	0.0000
0.0010	0.0051	0.0235	0.0142	0.0019	0.0109	0.0202	0.0126	0.0058
0.0029	0.0000	0.0029	0.0027	0.0008	0.0006	0.0086	0.0027	0.0000
0.0239	0.0023	0.0354	0.0300	0.0136	0.0136	0.0276	0.0245	0.0051
0.0016	0.0014	0.0062	0.0000	0.0016	0.0000	0.0025	0.0113	0.0000
0.0687	0.0370	0.0642	0.0290	0.0175	0.0261	0.0692	0.0815	0.0311
0.0000	0.0000	0.0066	0.0078	0.0000	0.0064	0.0235	0.0000	0.0000
0.0058	0.0000	0.0107	0.0121	0.0060	0.0080	0.0136	0.0082	0.0000
0.0220	0.0119	0.0169	0.0070	0.0078	0.0146	0.0226	0.0331	0.0097
0.0000	0.0000	0.0000	0.0000	0.0000	0.0000	0.0025	0.0000	0.0000
0.0082	0.0027	0.0237	0.0282	0.0058	0.0041	0.0241	0.0220	0.0033
0.0019	0.0002	0.0010	0.0006	0.0000	0.0000	0.0000	0.0035	0.0000
0.0000	0.0000	0.0000	0.0000	0.0000	0.0000	0.0000	0.0000	0.0000
0.0177	0.0066	0.0278	0.0224	0.0074	0.0062	0.0362	0.0362	0.0045
0.0000	0.0000	0.0068	0.0051	0.0000	0.0000	0.0000	0.0000	0.0000
0.0051	0.0016	0.0093	0.0062	0.0045	0.0000	0.0029	0.0163	0.0023
0.0023	0.0000	0.0021	0.0000	0.0014	0.0000	0.0000	0.0025	0.0000
0.0000	0.0025	0.0045	0.0033	0.0000	0.0012	0.0049	0.0086	0.0019
0.0068	0.0014	0.0222	0.0047	0.0054	0.0016	0.0062	0.0181	0.0023
0.0047	0.0000	0.0051	0.0000	0.0000	0.0000	0.0000	0.0068	0.0000
0.0027	0.0056	0.0019	0.0033	0.0000	0.0000	0.0080	0.0084	0.0035
0.0019	0.0000	0.0043	0.0023	0.0000	0.0000	0.0019	0.0037	0.0019
0.0012	0.0029	0.0019	0.0035	0.0023	0.0033	0.0019	0.0019	0.0027
0.0035	0.0012	0.0014	0.0066	0.0037	0.0033	0.0000	0.0078	0.0000
0.0037	0.0039	0.0000	0.0000	0.0000	0.0031	0.0051	0.0082	0.0084
0.0000	0.0000	0.0080	0.0021	0.0021	0.0000	0.0000	0.0006	0.0000
0.0000	0.0000	0.0000	0.0000	0.0000	0.0000	0.0000	0.0000	0.0000
0.0288	0.0375	0.0745	0.1692	0.1455	0.0741	0.0335	0.0253	0.0568
0.0422	0.0185	0.0292	0.0346	0.0599	0.0329	0.0196	0.0134	0.0399
0.0019	0.0014	0.0006	0.0043	0.0016	0.0016	0.0010	0.0021	0.0006
0.0000	0.0000	0.0000	0.0000	0.0000	0.0000	0.0004	0.0000	0.0000
0.0076	0.0076	0.0091	0.0064	0.0070	0.0095	0.0097	0.0086	0.0086
0.0035	0.0000	0.0101	0.0029	0.0025	0.0000	0.0086	0.0111	0.0056
0.0000	0.0002	0.0000	0.0000	0.0000	0.0000	0.0000	0.0000	0.0000
0.0000	0.0008	0.0000	0.0000	0.0000	0.0000	0.0000	0.0000	0.0000
0.0012	0.0000	0.0000	0.0006	0.0000	0.0000	0.0016	0.0004	0.0000

			Native microbiota, multiple gavage (3G)					
			3G D4					
M5	M6	M7	M1	M2	M3	M4	M5	M6
0.0000	0.0000	0.0000	0.0403	0.0556	0.0261	0.0218	0.0158	0.0095
0.0000	0.0004	0.0000	0.0000	0.0000	0.0000	0.0000	0.0000	0.0000
0.0000	0.0000	0.0000	0.0000	0.0000	0.0000	0.0000	0.0000	0.0000
0.0084	0.0158	0.0175	0.0109	0.0204	0.0088	0.0136	0.0101	0.0119
0.0000	0.0000	0.0000	0.0000	0.0000	0.0000	0.0000	0.0000	0.0000
0.0545	0.0335	0.0609	0.0196	0.0282	0.0288	0.0298	0.0432	0.0303
0.0148	0.0101	0.0099	0.0037	0.0068	0.0080	0.0103	0.0084	0.0074
0.2927	0.2085	0.3116	0.1780	0.2286	0.3097	0.2768	0.3470	0.2900
0.0115	0.0115	0.0171	0.0000	0.0016	0.0119	0.0014	0.0000	0.0074
0.0074	0.0051	0.0060	0.0000	0.0000	0.0023	0.0023	0.0010	0.0021
0.0000	0.0000	0.0000	0.0000	0.0000	0.0000	0.0000	0.0000	0.0000
0.0000	0.0000	0.0043	0.0000	0.0000	0.0000	0.0000	0.0000	0.0000
0.3721	0.3830	0.3295	0.1618	0.1202	0.0988	0.1657	0.1607	0.0720
0.0000	0.0000	0.0000	0.0000	0.0000	0.0000	0.0000	0.0000	0.0000
0.0000	0.0000	0.0000	0.0047	0.0019	0.0041	0.0039	0.0023	0.0010
0.0006	0.0000	0.0000	0.0000	0.0000	0.0000	0.0000	0.0000	0.0000
0.0000	0.0000	0.0000	0.0000	0.0000	0.0000	0.0000	0.0000	0.0000
0.0000	0.0000	0.0000	0.0000	0.0000	0.0000	0.0000	0.0000	0.0000
0.0179	0.0309	0.0043	0.0016	0.0111	0.0156	0.0105	0.0049	0.0545
0.0000	0.0000	0.0000	0.0000	0.0000	0.0000	0.0000	0.0000	0.0000
0.0000	0.0000	0.0000	0.0000	0.0000	0.0000	0.0000	0.0000	0.0000
0.0072	0.0064	0.0000	0.0000	0.0000	0.0000	0.0008	0.0000	0.0014
0.0000	0.0000	0.0000	0.0000	0.0000	0.0000	0.0000	0.0000	0.0023
0.0060	0.0235	0.0072	0.0179	0.0146	0.0208	0.0171	0.0109	0.0794
0.0000	0.0000	0.0000	0.0006	0.0021	0.0029	0.0021	0.0027	0.0140
0.0183	0.0249	0.0115	0.0000	0.0000	0.0054	0.0029	0.0006	0.0171
0.0000	0.0047	0.0000	0.0000	0.0000	0.0000	0.0000	0.0000	0.0010
0.0062	0.0076	0.0066	0.0000	0.0000	0.0000	0.0000	0.0000	0.0000
0.0035	0.0058	0.0054	0.0000	0.0000	0.0000	0.0000	0.0000	0.0000
0.0000	0.0000	0.0000	0.0000	0.0000	0.0000	0.0000	0.0000	0.0000
0.0043	0.0212	0.0019	0.0043	0.0051	0.0062	0.0138	0.0016	0.0272
0.0000	0.0008	0.0000	0.0000	0.0000	0.0000	0.0000	0.0000	0.0000
0.0000	0.0000	0.0000	0.0099	0.0051	0.0121	0.0093	0.0039	0.0045
0.0051	0.0097	0.0142	0.0000	0.0008	0.0051	0.0051	0.0008	0.0070
0.0066	0.0000	0.0000	0.0212	0.0183	0.0128	0.0193	0.0161	0.0307
0.0000	0.0031	0.0000	0.0000	0.0000	0.0000	0.0004	0.0000	0.0054
0.0000	0.0019	0.0012	0.0000	0.0004	0.0006	0.0012	0.0004	0.0031
0.0000	0.0014	0.0000	0.0000	0.0000	0.0000	0.0000	0.0000	0.0000
0.0021	0.0043	0.0025	0.0000	0.0000	0.0000	0.0000	0.0008	0.0016
0.0000	0.0000	0.0000	0.0000	0.0000	0.0000	0.0000	0.0000	0.0000
0.0000	0.0014	0.0000	0.0000	0.0000	0.0000	0.0000	0.0000	0.0000
0.0000	0.0021	0.0016	0.0008	0.0000	0.0000	0.0000	0.0012	0.0000
0.0010	0.0016	0.0031	0.0000	0.0000	0.0000	0.0000	0.0000	0.0000
0.0058	0.0056	0.0082	0.0008	0.0000	0.0023	0.0012	0.0280	0.0000
0.0000	0.0000	0.0000	0.0000	0.0000	0.0000	0.0000	0.0000	0.0000
0.0000	0.0023	0.0000	0.0000	0.0000	0.0000	0.0000	0.0000	0.0006
0.0000	0.0000	0.0000	0.0000	0.0000	0.0000	0.0000	0.0000	0.0000
0.0393	0.0881	0.0638	0.3779	0.3390	0.3038	0.2857	0.2225	0.2140
0.0475	0.0403	0.0459	0.0235	0.0189	0.0241	0.0206	0.0296	0.0128
0.0023	0.0012	0.0025	0.0004	0.0000	0.0006	0.0000	0.0000	0.0000
0.0000	0.0000	0.0000	0.0000	0.0000	0.0000	0.0000	0.0000	0.0000
0.0142	0.0031	0.0117	0.0000	0.0000	0.0000	0.0000	0.0000	0.0008
0.0037	0.0023	0.0043	0.0000	0.0000	0.0000	0.0000	0.0000	0.0000
0.0000	0.0000	0.0000	0.0000	0.0000	0.0000	0.0000	0.0000	0.0000
0.0000	0.0000	0.0000	0.0000	0.0000	0.0000	0.0000	0.0000	0.0000
0.0000	0.0000	0.0000	0.0885	0.0696	0.0342	0.0558	0.0393	0.0321

	3G D7							3G D14
M7	M1	M2	M3	M4	M5	M6	M7	M1
0.0130	0.0033	0.0027	0.0008	0.0132	0.0012	0.0006	0.0198	0.0000
0.0000	0.0008	0.0006	0.0000	0.0002	0.0000	0.0000	0.0000	0.0012
0.0000	0.0016	0.0006	0.0008	0.0000	0.0000	0.0000	0.0008	0.0010
0.0078	0.0167	0.0099	0.0123	0.0109	0.0054	0.0000	0.0084	0.0039
0.0000	0.0000	0.0000	0.0000	0.0000	0.0000	0.0000	0.0000	0.0000
0.0418	0.0405	0.0562	0.0624	0.0370	0.0558	0.0255	0.0282	0.0566
0.0062	0.0072	0.0097	0.0130	0.0062	0.0183	0.0000	0.0029	0.0088
0.3305	0.2179	0.3289	0.4312	0.2153	0.4766	0.1955	0.1756	0.2700
0.0000	0.0029	0.0084	0.0150	0.0076	0.0220	0.0000	0.0035	0.0136
0.0014	0.0004	0.0010	0.0023	0.0014	0.0016	0.0000	0.0012	0.0021
0.0000	0.0000	0.0000	0.0000	0.0000	0.0000	0.0000	0.0000	0.0000
0.0000	0.0000	0.0000	0.0000	0.0000	0.0000	0.0000	0.0000	0.0000
0.2428	0.1511	0.2042	0.2101	0.3237	0.2359	0.0060	0.2005	0.5106
0.0000	0.0000	0.0000	0.0008	0.0006	0.0000	0.0000	0.0008	0.0070
0.0021	0.0025	0.0021	0.0000	0.0012	0.0000	0.0000	0.0027	0.0000
0.0000	0.0000	0.0000	0.0000	0.0000	0.0000	0.0000	0.0000	0.0000
0.0000	0.0016	0.0000	0.0000	0.0000	0.0000	0.0021	0.0006	0.0000
0.0000	0.0014	0.0000	0.0000	0.0000	0.0000	0.0000	0.0000	0.0000
0.0123	0.0558	0.0091	0.0165	0.0117	0.0025	0.0000	0.0764	0.0029
0.0000	0.0041	0.0010	0.0000	0.0025	0.0000	0.0000	0.0027	0.0004
0.0000	0.0010	0.0000	0.0000	0.0000	0.0000	0.0000	0.0029	0.0000
0.0000	0.0049	0.0027	0.0056	0.0035	0.0000	0.2968	0.0130	0.0033
0.0000	0.0025	0.0012	0.0004	0.0000	0.0000	0.0000	0.0101	0.0000
0.0113	0.0788	0.0130	0.0119	0.0191	0.0158	0.0000	0.0482	0.0076
0.0021	0.0130	0.0000	0.0037	0.0033	0.0000	0.0000	0.0097	0.0010
0.0027	0.0772	0.0282	0.0309	0.0249	0.0179	0.0000	0.1157	0.0156
0.0000	0.0000	0.0027	0.0016	0.0000	0.0000	0.0000	0.0000	0.0029
0.0000	0.0130	0.0035	0.0019	0.0056	0.0023	0.0000	0.0185	0.0041
0.0000	0.0000	0.0000	0.0000	0.0000	0.0000	0.0000	0.0000	0.0008
0.0000	0.0000	0.0000	0.0000	0.0000	0.0000	0.0000	0.0000	0.0000
0.0037	0.0235	0.0072	0.0130	0.0064	0.0000	0.0023	0.0358	0.0008
0.0000	0.0000	0.0002	0.0002	0.0000	0.0000	0.0000	0.0008	0.0002
0.0039	0.0000	0.0004	0.0000	0.0000	0.0000	0.0000	0.0016	0.0000
0.0019	0.0156	0.0058	0.0134	0.0056	0.0088	0.0010	0.0161	0.0051
0.0284	0.0196	0.0235	0.0263	0.0216	0.0208	0.0000	0.0338	0.0000
0.0000	0.0062	0.0000	0.0010	0.0006	0.0000	0.0000	0.0054	0.0000
0.0002	0.0004	0.0000	0.0000	0.0000	0.0000	0.0000	0.0004	0.0000
0.0006	0.0008	0.0006	0.0006	0.0008	0.0000	0.0004	0.0047	0.0000
0.0004	0.0056	0.0008	0.0051	0.0014	0.0014	0.0109	0.0054	0.0004
0.0000	0.0000	0.0000	0.0000	0.0000	0.0000	0.0000	0.0000	0.0000
0.0000	0.0000	0.0010	0.0000	0.0000	0.0000	0.0000	0.0000	0.0000
0.0000	0.0000	0.0000	0.0000	0.0000	0.0000	0.0000	0.0000	0.0008
0.0000	0.0000	0.0008	0.0004	0.0029	0.0008	0.0000	0.0004	0.0008
0.0383	0.0010	0.0000	0.0000	0.0000	0.0000	0.0000	0.0006	0.0000
0.0023	0.0000	0.0000	0.0000	0.0008	0.0000	0.0000	0.0000	0.0078
0.0000	0.0047	0.0010	0.0025	0.0012	0.0016	0.0000	0.0058	0.0000
0.0000	0.0000	0.0000	0.0000	0.0000	0.0000	0.0000	0.0000	0.0000
0.1247	0.1519	0.1749	0.0356	0.1994	0.0257	0.0000	0.0370	0.0231
0.0152	0.0033	0.0082	0.0228	0.0058	0.0047	0.0000	0.0152	0.0051
0.0000	0.0008	0.0006	0.0031	0.0000	0.0010	0.0000	0.0014	0.0008
0.0000	0.0000	0.0000	0.0000	0.0000	0.0000	0.0000	0.0004	0.0006
0.0000	0.0126	0.0021	0.0019	0.0016	0.0006	0.0000	0.0037	0.0010
0.0000	0.0000	0.0000	0.0000	0.0000	0.0000	0.0000	0.0004	0.0000
0.0000	0.0000	0.0016	0.0010	0.0004	0.0010	0.0000	0.0029	0.0000
0.0000	0.0000	0.0000	0.0000	0.0000	0.0000	0.0000	0.0000	0.0000
0.0362	0.0222	0.0265	0.0088	0.0333	0.0097	0.2643	0.0136	0.0021

						3G D21		
M2	M3	M4	M5	M6	M7	M1	M2	M3
0.0000	0.0006	0.0033	0.0000	0.0000	0.0000	0.0002	0.0000	0.0000
0.0000	0.0060	0.0021	0.0000	0.0033	0.0000	0.0140	0.0070	0.0021
0.0000	0.0004	0.0000	0.0000	0.0008	0.0004	0.0000	0.0000	0.0000
0.0144	0.0062	0.0066	0.0093	0.0064	0.0101	0.0117	0.0074	0.0033
0.0000	0.0000	0.0000	0.0000	0.0000	0.0000	0.0000	0.0000	0.0000
0.0689	0.0564	0.0521	0.0587	0.0496	0.0403	0.0580	0.0700	0.0473
0.0082	0.0080	0.0099	0.0062	0.0095	0.0076	0.0062	0.0093	0.0066
0.3001	0.2842	0.3108	0.2883	0.3217	0.2035	0.3005	0.3515	0.3569
0.0196	0.0136	0.0150	0.0091	0.0187	0.0012	0.0039	0.0070	0.0123
0.0008	0.0021	0.0027	0.0070	0.0035	0.0004	0.0016	0.0014	0.0025
0.0000	0.0000	0.0000	0.0016	0.0021	0.0012	0.0000	0.0000	0.0000
0.0000	0.0000	0.0000	0.0084	0.0078	0.0016	0.0000	0.0000	0.0000
0.4694	0.1367	0.4149	0.4332	0.3750	0.2105	0.2614	0.3626	0.4149
0.0033	0.0021	0.0101	0.0027	0.0070	0.0019	0.0004	0.0066	0.0043
0.0000	0.0045	0.0056	0.0000	0.0000	0.0000	0.0000	0.0000	0.0165
0.0000	0.0000	0.0000	0.0000	0.0000	0.0000	0.0000	0.0000	0.0000
0.0000	0.0029	0.0000	0.0000	0.0000	0.0023	0.0029	0.0002	0.0000
0.0000	0.0008	0.0000	0.0000	0.0000	0.0012	0.0006	0.0000	0.0000
0.0047	0.0737	0.0035	0.0027	0.0115	0.0895	0.0506	0.0103	0.0070
0.0025	0.0072	0.0000	0.0000	0.0021	0.0012	0.0126	0.0019	0.0002
0.0000	0.0047	0.0000	0.0010	0.0000	0.0049	0.0000	0.0012	0.0000
0.0006	0.0099	0.0027	0.0037	0.0097	0.0161	0.0027	0.0045	0.0016
0.0000	0.0060	0.0000	0.0000	0.0000	0.0086	0.0006	0.0000	0.0000
0.0099	0.0391	0.0093	0.0138	0.0175	0.0263	0.0463	0.0156	0.0101
0.0000	0.0156	0.0000	0.0000	0.0049	0.0202	0.0086	0.0000	0.0014
0.0068	0.0726	0.0245	0.0220	0.0119	0.0731	0.0506	0.0245	0.0084
0.0019	0.0148	0.0035	0.0027	0.0008	0.0165	0.0000	0.0086	0.0012
0.0000	0.0171	0.0062	0.0037	0.0070	0.0278	0.0243	0.0080	0.0058
0.0033	0.0206	0.0008	0.0000	0.0000	0.0109	0.0021	0.0043	0.0025
0.0000	0.0000	0.0000	0.0000	0.0000	0.0000	0.0000	0.0000	0.0000
0.0012	0.0321	0.0027	0.0010	0.0070	0.0531	0.0115	0.0074	0.0004
0.0004	0.0002	0.0000	0.0000	0.0002	0.0004	0.0016	0.0000	0.0000
0.0000	0.0012	0.0014	0.0000	0.0000	0.0000	0.0000	0.0000	0.0000
0.0049	0.0140	0.0047	0.0072	0.0049	0.0231	0.0088	0.0062	0.0056
0.0000	0.0012	0.0066	0.0056	0.0000	0.0006	0.0045	0.0000	0.0014
0.0000	0.0037	0.0000	0.0000	0.0000	0.0025	0.0000	0.0000	0.0006
0.0000	0.0000	0.0000	0.0000	0.0000	0.0000	0.0000	0.0000	0.0000
0.0000	0.0039	0.0000	0.0000	0.0010	0.0033	0.0000	0.0019	0.0000
0.0008	0.0082	0.0010	0.0019	0.0000	0.0158	0.0062	0.0010	0.0000
0.0000	0.0004	0.0000	0.0000	0.0000	0.0000	0.0000	0.0000	0.0000
0.0006	0.0049	0.0012	0.0004	0.0019	0.0035	0.0000	0.0008	0.0000
0.0000	0.0023	0.0000	0.0010	0.0004	0.0014	0.0010	0.0000	0.0000
0.0006	0.0010	0.0010	0.0010	0.0016	0.0000	0.0006	0.0000	0.0004
0.0008	0.0000	0.0000	0.0000	0.0016	0.0000	0.0010	0.0000	0.0004
0.0039	0.0029	0.0072	0.0064	0.0109	0.0029	0.0167	0.0123	0.0037
0.0000	0.0012	0.0000	0.0000	0.0008	0.0000	0.0008	0.0000	0.0000
0.0000	0.0000	0.0000	0.0000	0.0000	0.0000	0.0000	0.0000	0.0000
0.0119	0.0021	0.0136	0.0235	0.0222	0.0163	0.0033	0.0177	0.0072
0.0062	0.0152	0.0391	0.0000	0.0000	0.0000	0.0000	0.0000	0.0315
0.0006	0.0014	0.0006	0.0012	0.0019	0.0002	0.0004	0.0008	0.0021
0.0021	0.0010	0.0000	0.0037	0.0008	0.0025	0.0008	0.0000	0.0025
0.0031	0.0187	0.0000	0.0031	0.0035	0.0173	0.0179	0.0049	0.0000
0.0004	0.0008	0.0035	0.0000	0.0006	0.0000	0.0008	0.0016	0.0064
0.0000	0.0000	0.0000	0.0006	0.0000	0.0010	0.0031	0.0010	0.0002
0.0000	0.0000	0.0000	0.0000	0.0000	0.0000	0.0000	0.0000	0.0000
0.0062	0.0249	0.0080	0.0101	0.0091	0.0037	0.0035	0.0008	0.0161

				3G D28				
M4	M5	M6	M7	M1	M2	M3	M4	M5
0.0000	0.0000	0.0000	0.0004	0.0006	0.0000	0.0000	0.0000	0.0000
0.0068	0.0076	0.0045	0.0076	0.0037	0.0021	0.0111	0.0000	0.0023
0.0006	0.0000	0.0000	0.0000	0.0000	0.0000	0.0000	0.0000	0.0000
0.0128	0.0086	0.0047	0.0101	0.0060	0.0039	0.0049	0.0138	0.0062
0.0000	0.0000	0.0000	0.0000	0.0000	0.0000	0.0000	0.0000	0.0000
0.0681	0.0661	0.0957	0.0632	0.0698	0.0562	0.0410	0.0589	0.0549
0.0025	0.0066	0.0091	0.0101	0.0072	0.0068	0.0078	0.0062	0.0062
0.2056	0.2338	0.3628	0.2797	0.3159	0.2764	0.2404	0.2612	0.2289
0.0025	0.0027	0.0148	0.0041	0.0084	0.0103	0.0037	0.0068	0.0097
0.0012	0.0008	0.0006	0.0014	0.0000	0.0012	0.0049	0.0002	0.0006
0.0000	0.0000	0.0000	0.0000	0.0000	0.0000	0.0000	0.0000	0.0076
0.0000	0.0000	0.0000	0.0000	0.0000	0.0000	0.0000	0.0000	0.0111
0.4435	0.2040	0.1544	0.1848	0.4225	0.4954	0.2552	0.4100	0.4659
0.0047	0.0002	0.0041	0.0006	0.0037	0.0021	0.0000	0.0049	0.0064
0.0134	0.0000	0.0000	0.0000	0.0000	0.0000	0.0113	0.0198	0.0000
0.0000	0.0000	0.0000	0.0000	0.0000	0.0000	0.0000	0.0000	0.0000
0.0002	0.0019	0.0000	0.0021	0.0000	0.0000	0.0029	0.0000	0.0000
0.0000	0.0019	0.0006	0.0021	0.0000	0.0000	0.0008	0.0000	0.0000
0.0121	0.0772	0.0335	0.0543	0.0054	0.0027	0.0525	0.0027	0.0134
0.0037	0.0054	0.0012	0.0158	0.0025	0.0025	0.0066	0.0014	0.0041
0.0000	0.0010	0.0000	0.0000	0.0000	0.0000	0.0008	0.0000	0.0004
0.0023	0.0056	0.0060	0.0000	0.0000	0.0000	0.0010	0.0000	0.0101
0.0000	0.0029	0.0025	0.0027	0.0000	0.0000	0.0023	0.0000	0.0000
0.0179	0.0790	0.0407	0.0549	0.0111	0.0054	0.0348	0.0068	0.0068
0.0004	0.0014	0.0041	0.0088	0.0014	0.0014	0.0056	0.0004	0.0023
0.0294	0.0451	0.0630	0.0819	0.0177	0.0161	0.0912	0.0243	0.0401
0.0019	0.0082	0.0076	0.0078	0.0008	0.0049	0.0000	0.0000	0.0140
0.0086	0.0239	0.0093	0.0385	0.0072	0.0072	0.0140	0.0039	0.0033
0.0029	0.0204	0.0084	0.0158	0.0021	0.0016	0.0200	0.0023	0.0097
0.0000	0.0025	0.0000	0.0086	0.0000	0.0000	0.0091	0.0000	0.0000
0.0068	0.0502	0.0134	0.0228	0.0035	0.0035	0.0072	0.0021	0.0047
0.0002	0.0000	0.0010	0.0000	0.0000	0.0002	0.0004	0.0000	0.0012
0.0000	0.0000	0.0000	0.0000	0.0000	0.0000	0.0000	0.0000	0.0000
0.0045	0.0099	0.0158	0.0084	0.0054	0.0037	0.0064	0.0027	0.0093
0.0084	0.0012	0.0000	0.0080	0.0043	0.0000	0.0027	0.0095	0.0107
0.0000	0.0008	0.0019	0.0000	0.0000	0.0000	0.0012	0.0000	0.0000
0.0002	0.0006	0.0000	0.0000	0.0006	0.0000	0.0000	0.0004	0.0000
0.0004	0.0000	0.0016	0.0000	0.0000	0.0004	0.0016	0.0000	0.0014
0.0008	0.0060	0.0062	0.0033	0.0014	0.0000	0.0031	0.0000	0.0000
0.0000	0.0010	0.0000	0.0000	0.0000	0.0000	0.0000	0.0000	0.0000
0.0000	0.0000	0.0000	0.0000	0.0000	0.0000	0.0000	0.0000	0.0000
0.0000	0.0027	0.0016	0.0016	0.0000	0.0000	0.0000	0.0000	0.0000
0.0012	0.0010	0.0012	0.0006	0.0014	0.0010	0.0016	0.0006	0.0004
0.0002	0.0000	0.0000	0.0000	0.0000	0.0000	0.0002	0.0000	0.0000
0.0181	0.0111	0.0196	0.0080	0.0105	0.0119	0.0144	0.0208	0.0132
0.0000	0.0010	0.0002	0.0037	0.0012	0.0000	0.0019	0.0008	0.0016
0.0000	0.0000	0.0000	0.0000	0.0000	0.0000	0.0000	0.0000	0.0000
0.0045	0.0023	0.0019	0.0006	0.0198	0.0278	0.0023	0.0095	0.0058
0.0218	0.0000	0.0000	0.0000	0.0000	0.0000	0.0265	0.0467	0.0000
0.0016	0.0008	0.0033	0.0004	0.0012	0.0008	0.0000	0.0023	0.0006
0.0021	0.0016	0.0012	0.0039	0.0012	0.0006	0.0058	0.0039	0.0006
0.0068	0.0292	0.0080	0.0177	0.0016	0.0025	0.0338	0.0029	0.0037
0.0025	0.0000	0.0006	0.0008	0.0008	0.0016	0.0027	0.0033	0.0008
0.0060	0.0000	0.0014	0.0027	0.0016	0.0099	0.0045	0.0029	0.0012
0.0000	0.0000	0.0000	0.0000	0.0000	0.0000	0.0000	0.0000	0.0000
0.0490	0.0068	0.0111	0.0012	0.0004	0.0000	0.0317	0.0410	0.0058

M6	M7	3G D35 M1	M2	M3	M4	M5	M6	M7
0.0000	0.0000	0.0000	0.0000	0.0047	0.0115	0.0000	0.0000	0.0000
0.0051	0.0049	0.0000	0.0000	0.0025	0.0004	0.0000	0.0000	0.0000
0.0004	0.0000	0.0000	0.0004	0.0021	0.0019	0.0006	0.0016	0.0012
0.0091	0.0062	0.0146	0.0144	0.0263	0.0226	0.0163	0.0247	0.0255
0.0000	0.0000	0.0000	0.0000	0.0000	0.0000	0.0000	0.0000	0.0000
0.0745	0.0519	0.0354	0.0290	0.0307	0.0208	0.0342	0.0407	0.0403
0.0051	0.0016	0.0049	0.0082	0.0058	0.0068	0.0027	0.0070	0.0082
0.2334	0.2342	0.1858	0.1509	0.1515	0.1155	0.1803	0.2027	0.2019
0.0086	0.0070	0.0000	0.0058	0.0000	0.0045	0.0051	0.0056	0.0047
0.0000	0.0000	0.0016	0.0033	0.0021	0.0029	0.0016	0.0031	0.0012
0.0010	0.0012	0.0000	0.0000	0.0000	0.0000	0.0000	0.0000	0.0000
0.0012	0.0019	0.0000	0.0000	0.0000	0.0000	0.0000	0.0000	0.0000
0.5020	0.5839	0.1525	0.3807	0.3359	0.4489	0.4077	0.2562	0.2422
0.0060	0.0008	0.0000	0.0033	0.0002	0.0037	0.0000	0.0056	0.0000
0.0000	0.0000	0.0000	0.0000	0.0072	0.0045	0.0000	0.0000	0.0000
0.0000	0.0000	0.0000	0.0000	0.0000	0.0000	0.0000	0.0000	0.0000
0.0000	0.0000	0.0033	0.0019	0.0045	0.0000	0.0010	0.0016	0.0029
0.0000	0.0000	0.0031	0.0000	0.0016	0.0000	0.0000	0.0000	0.0029
0.0099	0.0029	0.0836	0.0564	0.0722	0.0642	0.0418	0.0661	0.0486
0.0008	0.0019	0.0150	0.0177	0.0154	0.0058	0.0060	0.0064	0.0228
0.0000	0.0000	0.0014	0.0016	0.0000	0.0035	0.0000	0.0086	0.0012
0.0000	0.0000	0.0142	0.0000	0.0000	0.0000	0.0000	0.0049	0.0021
0.0000	0.0000	0.0047	0.0021	0.0056	0.0021	0.0016	0.0027	0.0064
0.0080	0.0035	0.0274	0.0165	0.0327	0.0084	0.0272	0.0202	0.0278
0.0000	0.0014	0.0146	0.0082	0.0121	0.0091	0.0115	0.0066	0.0175
0.0274	0.0031	0.0741	0.0358	0.0630	0.0521	0.0309	0.0440	0.0430
0.0000	0.0002	0.0220	0.0128	0.0014	0.0000	0.0136	0.0000	0.0027
0.0049	0.0064	0.0148	0.0076	0.0095	0.0051	0.0068	0.0056	0.0152
0.0045	0.0000	0.0144	0.0099	0.0076	0.0047	0.0107	0.0103	0.0171
0.0000	0.0000	0.0117	0.0014	0.0084	0.0000	0.0033	0.0000	0.0084
0.0002	0.0006	0.0535	0.0134	0.0144	0.0023	0.0222	0.0078	0.0206
0.0000	0.0000	0.0045	0.0031	0.0008	0.0000	0.0014	0.0008	0.0014
0.0000	0.0000	0.0000	0.0000	0.0000	0.0000	0.0000	0.0000	0.0000
0.0054	0.0006	0.0375	0.0329	0.0146	0.0136	0.0202	0.0175	0.0231
0.0000	0.0021	0.0064	0.0027	0.0051	0.0000	0.0068	0.0105	0.0119
0.0000	0.0000	0.0082	0.0019	0.0033	0.0064	0.0023	0.0051	0.0006
0.0000	0.0000	0.0000	0.0010	0.0037	0.0000	0.0014	0.0037	0.0031
0.0000	0.0002	0.0074	0.0049	0.0021	0.0039	0.0039	0.0140	0.0064
0.0006	0.0039	0.0150	0.0076	0.0076	0.0054	0.0109	0.0173	0.0189
0.0000	0.0000	0.0000	0.0000	0.0000	0.0000	0.0000	0.0000	0.0000
0.0000	0.0000	0.0019	0.0000	0.0000	0.0037	0.0000	0.0006	0.0000
0.0000	0.0000	0.0051	0.0041	0.0000	0.0012	0.0012	0.0025	0.0000
0.0002	0.0006	0.0019	0.0014	0.0016	0.0008	0.0012	0.0021	0.0033
0.0000	0.0000	0.0000	0.0010	0.0010	0.0000	0.0010	0.0045	0.0041
0.0099	0.0021	0.0247	0.0231	0.0202	0.0167	0.0212	0.0381	0.0282
0.0000	0.0019	0.0119	0.0068	0.0054	0.0051	0.0060	0.0054	0.0130
0.0000	0.0000	0.0000	0.0000	0.0000	0.0000	0.0000	0.0000	0.0000
0.0084	0.0105	0.0080	0.0103	0.0117	0.0181	0.0078	0.0054	0.0031
0.0000	0.0000	0.0000	0.0000	0.0171	0.0158	0.0000	0.0000	0.0000
0.0010	0.0008	0.0004	0.0016	0.0025	0.0023	0.0027	0.0041	0.0019
0.0072	0.0041	0.0021	0.0076	0.0014	0.0058	0.0012	0.0041	0.0047
0.0058	0.0093	0.0103	0.0074	0.0173	0.0033	0.0109	0.0056	0.0189
0.0021	0.0002	0.0000	0.0088	0.0066	0.0128	0.0035	0.0093	0.0068
0.0031	0.0002	0.0132	0.0086	0.0058	0.0086	0.0091	0.0187	0.0068
0.0000	0.0000	0.0000	0.0002	0.0000	0.0000	0.0000	0.0002	0.0000
0.0047	0.0068	0.0000	0.0002	0.0113	0.0078	0.0000	0.0000	0.0014

3G D42							3G D49	
M1	M2	M3	M4	M5	M6	M7	M1	M2
0.0000	0.0000	0.0014	0.0049	0.0000	0.0000	0.0000	0.0000	0.0025
0.0000	0.0000	0.0000	0.0000	0.0000	0.0000	0.0000	0.0000	0.0012
0.0000	0.0023	0.0019	0.0019	0.0000	0.0000	0.0000	0.0000	0.0000
0.0093	0.0235	0.0214	0.0204	0.0335	0.0241	0.0251	0.0105	0.0175
0.0000	0.0000	0.0000	0.0000	0.0000	0.0000	0.0000	0.0000	0.0000
0.0317	0.0183	0.0459	0.0556	0.0560	0.0531	0.0445	0.0327	0.0331
0.0045	0.0051	0.0107	0.0121	0.0152	0.0123	0.0099	0.0117	0.0058
0.1832	0.1103	0.1644	0.2593	0.2542	0.1990	0.2334	0.1725	0.2122
0.0051	0.0078	0.0043	0.0099	0.0101	0.0064	0.0093	0.0074	0.0064
0.0023	0.0000	0.0060	0.0031	0.0023	0.0014	0.0014	0.0019	0.0010
0.0000	0.0000	0.0000	0.0000	0.0043	0.0000	0.0000	0.0000	0.0000
0.0000	0.0000	0.0000	0.0000	0.0214	0.0144	0.0132	0.0000	0.0000
0.4028	0.4801	0.1743	0.1967	0.2002	0.3470	0.2863	0.1644	0.4046
0.0000	0.0107	0.0004	0.0012	0.0008	0.0056	0.0045	0.0000	0.0051
0.0016	0.0012	0.0047	0.0033	0.0000	0.0006	0.0000	0.0029	0.0091
0.0000	0.0000	0.0000	0.0000	0.0000	0.0000	0.0000	0.0000	0.0000
0.0012	0.0006	0.0051	0.0019	0.0000	0.0014	0.0000	0.0023	0.0000
0.0000	0.0000	0.0037	0.0000	0.0000	0.0000	0.0000	0.0014	0.0002
0.0352	0.0451	0.1122	0.0370	0.0338	0.0381	0.0231	0.0836	0.0066
0.0117	0.0163	0.0138	0.0187	0.0060	0.0101	0.0134	0.0146	0.0082
0.0000	0.0012	0.0000	0.0025	0.0000	0.0008	0.0027	0.0027	0.0025
0.0029	0.0000	0.0000	0.0000	0.0119	0.0103	0.0016	0.0051	0.0041
0.0023	0.0000	0.0027	0.0033	0.0029	0.0000	0.0010	0.0068	0.0016
0.0198	0.0202	0.0340	0.0218	0.0163	0.0255	0.0226	0.0177	0.0148
0.0010	0.0045	0.0165	0.0029	0.0060	0.0045	0.0049	0.0158	0.0088
0.0453	0.0329	0.0850	0.0609	0.0412	0.0463	0.0477	0.0860	0.0373
0.0134	0.0066	0.0000	0.0121	0.0243	0.0000	0.0021	0.0249	0.0066
0.0064	0.0054	0.0179	0.0117	0.0119	0.0099	0.0105	0.0156	0.0058
0.0025	0.0054	0.0119	0.0115	0.0117	0.0109	0.0058	0.0136	0.0150
0.0000	0.0000	0.0000	0.0000	0.0056	0.0014	0.0041	0.0000	0.0023
0.0204	0.0056	0.0204	0.0101	0.0344	0.0257	0.0097	0.0391	0.0051
0.0002	0.0021	0.0035	0.0031	0.0008	0.0000	0.0012	0.0045	0.0014
0.0000	0.0000	0.0000	0.0000	0.0000	0.0000	0.0000	0.0000	0.0000
0.0156	0.0158	0.0397	0.0130	0.0080	0.0121	0.0202	0.0385	0.0261
0.0045	0.0000	0.0000	0.0105	0.0070	0.0000	0.0082	0.0000	0.0072
0.0021	0.0019	0.0000	0.0000	0.0029	0.0019	0.0010	0.0128	0.0010
0.0000	0.0004	0.0000	0.0023	0.0000	0.0014	0.0000	0.0000	0.0000
0.0045	0.0039	0.0000	0.0049	0.0105	0.0029	0.0060	0.0177	0.0029
0.0099	0.0000	0.0274	0.0074	0.0064	0.0049	0.0158	0.0224	0.0097
0.0000	0.0000	0.0035	0.0000	0.0000	0.0000	0.0000	0.0000	0.0000
0.0025	0.0000	0.0000	0.0000	0.0039	0.0031	0.0000	0.0000	0.0000
0.0033	0.0000	0.0111	0.0039	0.0070	0.0000	0.0012	0.0025	0.0016
0.0008	0.0010	0.0010	0.0025	0.0043	0.0016	0.0031	0.0027	0.0021
0.0010	0.0023	0.0000	0.0000	0.0000	0.0012	0.0019	0.0010	0.0016
0.0206	0.0259	0.0148	0.0331	0.0204	0.0239	0.0552	0.0132	0.0216
0.0074	0.0035	0.0043	0.0054	0.0066	0.0051	0.0047	0.0105	0.0023
0.0000	0.0000	0.0000	0.0000	0.0000	0.0000	0.0000	0.0000	0.0000
0.0418	0.0840	0.0082	0.0177	0.0086	0.0070	0.0000	0.0056	0.0119
0.0000	0.0000	0.0200	0.0243	0.0000	0.0000	0.0000	0.0091	0.0068
0.0025	0.0014	0.0004	0.0045	0.0023	0.0010	0.0058	0.0006	0.0006
0.0010	0.0029	0.0012	0.0025	0.0019	0.0027	0.0051	0.0054	0.0016
0.0025	0.0043	0.0150	0.0082	0.0123	0.0080	0.0035	0.0128	0.0076
0.0047	0.0043	0.0119	0.0031	0.0086	0.0091	0.0049	0.0023	0.0109
0.0105	0.0091	0.0012	0.0109	0.0058	0.0031	0.0212	0.0070	0.0051
0.0010	0.0000	0.0000	0.0010	0.0012	0.0000	0.0000	0.0002	0.0000
0.0000	0.0000	0.0111	0.0183	0.0014	0.0000	0.0000	0.0000	0.0008

					3G D56			
M3	M4	M5	M6	M7	M1	M2	M3	M4
0.0051	0.0047	0.0000	0.0000	0.0000	0.0000	0.0000	0.0000	0.0000
0.0000	0.0000	0.0000	0.0010	0.0000	0.0000	0.0000	0.0000	0.0000
0.0000	0.0004	0.0006	0.0021	0.0023	0.0000	0.0014	0.0002	0.0010
0.0107	0.0130	0.0163	0.0173	0.0163	0.0216	0.0138	0.0136	0.0171
0.0000	0.0000	0.0000	0.0000	0.0000	0.0000	0.0000	0.0000	0.0000
0.0309	0.0459	0.0391	0.0352	0.0344	0.0420	0.0420	0.0313	0.0440
0.0080	0.0103	0.0084	0.0051	0.0072	0.0105	0.0082	0.0066	0.0095
0.1496	0.2299	0.2087	0.1478	0.2031	0.2361	0.2307	0.1700	0.2089
0.0049	0.0091	0.0080	0.0060	0.0088	0.0086	0.0051	0.0097	0.0062
0.0039	0.0039	0.0012	0.0023	0.0008	0.0039	0.0023	0.0039	0.0029
0.0000	0.0000	0.0000	0.0000	0.0000	0.0000	0.0000	0.0000	0.0000
0.0000	0.0000	0.0068	0.0037	0.0054	0.0000	0.0000	0.0000	0.0000
0.4260	0.2813	0.4042	0.2391	0.5172	0.3021	0.4651	0.3997	0.3418
0.0066	0.0008	0.0080	0.0019	0.0021	0.0000	0.0000	0.0000	0.0010
0.0121	0.0095	0.0035	0.0008	0.0000	0.0000	0.0074	0.0093	0.0109
0.0000	0.0000	0.0000	0.0000	0.0000	0.0000	0.0000	0.0002	0.0006
0.0000	0.0000	0.0014	0.0049	0.0006	0.0000	0.0000	0.0002	0.0021
0.0000	0.0000	0.0000	0.0041	0.0000	0.0000	0.0000	0.0000	0.0016
0.0554	0.0397	0.0140	0.1023	0.0156	0.0263	0.0000	0.0519	0.0418
0.0109	0.0111	0.0084	0.0109	0.0064	0.0093	0.0068	0.0025	0.0146
0.0000	0.0010	0.0000	0.0027	0.0000	0.0010	0.0000	0.0025	0.0000
0.0000	0.0000	0.0023	0.0161	0.0000	0.0019	0.0000	0.0000	0.0000
0.0000	0.0010	0.0031	0.0068	0.0021	0.0043	0.0000	0.0014	0.0014
0.0196	0.0105	0.0156	0.0303	0.0117	0.0099	0.0161	0.0103	0.0107
0.0021	0.0060	0.0056	0.0078	0.0012	0.0058	0.0000	0.0062	0.0041
0.0698	0.0617	0.0500	0.0702	0.0233	0.0996	0.0214	0.0761	0.0644
0.0000	0.0000	0.0000	0.0000	0.0000	0.0000	0.0031	0.0000	0.0000
0.0068	0.0066	0.0084	0.0167	0.0064	0.0165	0.0109	0.0031	0.0078
0.0119	0.0136	0.0189	0.0218	0.0097	0.0111	0.0049	0.0068	0.0140
0.0000	0.0000	0.0037	0.0080	0.0010	0.0000	0.0000	0.0000	0.0000
0.0012	0.0134	0.0169	0.0496	0.0010	0.0130	0.0021	0.0008	0.0113
0.0014	0.0006	0.0000	0.0025	0.0008	0.0014	0.0000	0.0014	0.0006
0.0000	0.0000	0.0000	0.0000	0.0000	0.0000	0.0000	0.0000	0.0000
0.0132	0.0173	0.0058	0.0144	0.0056	0.0189	0.0039	0.0200	0.0144
0.0000	0.0010	0.0134	0.0000	0.0060	0.0000	0.0056	0.0000	0.0000
0.0019	0.0021	0.0023	0.0076	0.0000	0.0025	0.0000	0.0031	0.0029
0.0000	0.0000	0.0000	0.0016	0.0008	0.0000	0.0000	0.0000	0.0008
0.0019	0.0027	0.0023	0.0029	0.0010	0.0054	0.0010	0.0095	0.0023
0.0000	0.0025	0.0056	0.0179	0.0035	0.0027	0.0014	0.0004	0.0021
0.0000	0.0000	0.0000	0.0035	0.0000	0.0000	0.0000	0.0002	0.0000
0.0000	0.0000	0.0000	0.0000	0.0000	0.0033	0.0000	0.0000	0.0000
0.0000	0.0010	0.0000	0.0010	0.0019	0.0000	0.0010	0.0070	0.0000
0.0016	0.0019	0.0025	0.0012	0.0010	0.0014	0.0016	0.0012	0.0021
0.0000	0.0021	0.0004	0.0010	0.0027	0.0000	0.0027	0.0016	0.0006
0.0202	0.0342	0.0305	0.0144	0.0373	0.0088	0.0181	0.0165	0.0231
0.0000	0.0010	0.0037	0.0029	0.0000	0.0000	0.0031	0.0000	0.0027
0.0000	0.0000	0.0000	0.0000	0.0000	0.0000	0.0000	0.0000	0.0000
0.0315	0.0200	0.0119	0.0051	0.0076	0.0101	0.0097	0.0144	0.0113
0.0387	0.0626	0.0000	0.0000	0.0000	0.0253	0.0436	0.0288	0.0486
0.0016	0.0012	0.0002	0.0006	0.0072	0.0012	0.0035	0.0023	0.0008
0.0000	0.0033	0.0068	0.0000	0.0016	0.0086	0.0029	0.0031	0.0047
0.0091	0.0095	0.0093	0.0128	0.0051	0.0103	0.0066	0.0074	0.0113
0.0043	0.0025	0.0000	0.0019	0.0000	0.0033	0.0000	0.0054	0.0021
0.0027	0.0121	0.0060	0.0035	0.0035	0.0109	0.0091	0.0158	0.0086
0.0004	0.0000	0.0000	0.0006	0.0000	0.0000	0.0000	0.0000	0.0004
0.0142	0.0181	0.0000	0.0000	0.0000	0.0000	0.0000	0.0097	0.0138

			3G D63					
**M5**	**M6**	**M7**	**M1**	**M2**	**M3**	**M4**	**M5**	**M6**
0.0000	0.0000	0.0000	0.0091	0.0000	0.0000	0.0000	0.0000	0.0000
0.0031	0.0000	0.0000	0.0000	0.0000	0.0000	0.0000	0.0021	0.0000
0.0012	0.0000	0.0012	0.0033	0.0023	0.0014	0.0014	0.0000	0.0021
0.0156	0.0300	0.0249	0.0212	0.0226	0.0183	0.0179	0.0163	0.0204
0.0000	0.0000	0.0000	0.0000	0.0000	0.0000	0.0000	0.0000	0.0000
0.0397	0.0632	0.0492	0.0537	0.0370	0.0387	0.0504	0.0541	0.0593
0.0103	0.0146	0.0103	0.0128	0.0099	0.0066	0.0088	0.0091	0.0107
0.2344	0.2809	0.2381	0.2101	0.2075	0.2128	0.1961	0.2830	0.2628
0.0064	0.0101	0.0062	0.0064	0.0000	0.0097	0.0078	0.0097	0.0066
0.0037	0.0010	0.0008	0.0014	0.0023	0.0012	0.0014	0.0043	0.0012
0.0093	0.0119	0.0074	0.0000	0.0000	0.0000	0.0000	0.0000	0.0000
0.0156	0.0165	0.0117	0.0000	0.0000	0.0000	0.0000	0.0000	0.0000
0.2046	0.2857	0.5297	0.3904	0.3548	0.3546	0.3583	0.3626	0.2706
0.0010	0.0091	0.0002	0.0000	0.0000	0.0000	0.0080	0.0041	0.0037
0.0070	0.0000	0.0000	0.0000	0.0000	0.0088	0.0082	0.0070	0.0103
0.0000	0.0000	0.0000	0.0000	0.0000	0.0000	0.0000	0.0000	0.0000
0.0041	0.0000	0.0000	0.0012	0.0000	0.0002	0.0016	0.0000	0.0000
0.0016	0.0000	0.0000	0.0000	0.0010	0.0010	0.0008	0.0000	0.0000
0.0634	0.0282	0.0033	0.0113	0.0284	0.0508	0.0393	0.0224	0.0307
0.0189	0.0084	0.0113	0.0093	0.0245	0.0076	0.0103	0.0060	0.0064
0.0045	0.0000	0.0000	0.0000	0.0000	0.0006	0.0000	0.0000	0.0062
0.0088	0.0064	0.0000	0.0016	0.0000	0.0000	0.0000	0.0021	0.0037
0.0043	0.0000	0.0000	0.0021	0.0043	0.0025	0.0031	0.0019	0.0000
0.0249	0.0115	0.0000	0.0054	0.0233	0.0107	0.0037	0.0054	0.0093
0.0099	0.0000	0.0000	0.0008	0.0000	0.0056	0.0037	0.0035	0.0051
0.0638	0.0373	0.0181	0.0393	0.0681	0.0609	0.0622	0.0370	0.0362
0.0000	0.0000	0.0000	0.0000	0.0049	0.0000	0.0000	0.0000	0.0000
0.0047	0.0097	0.0029	0.0109	0.0130	0.0111	0.0126	0.0000	0.0103
0.0233	0.0156	0.0027	0.0086	0.0107	0.0237	0.0185	0.0247	0.0177
0.0000	0.0000	0.0000	0.0000	0.0043	0.0000	0.0000	0.0000	0.0000
0.0216	0.0119	0.0000	0.0058	0.0107	0.0074	0.0111	0.0031	0.0119
0.0016	0.0012	0.0008	0.0000	0.0000	0.0000	0.0004	0.0012	0.0010
0.0000	0.0000	0.0000	0.0000	0.0000	0.0000	0.0000	0.0000	0.0000
0.0261	0.0142	0.0027	0.0128	0.0080	0.0097	0.0119	0.0179	0.0144
0.0060	0.0000	0.0000	0.0000	0.0045	0.0002	0.0000	0.0076	0.0000
0.0000	0.0025	0.0000	0.0019	0.0019	0.0016	0.0041	0.0000	0.0037
0.0000	0.0000	0.0000	0.0000	0.0000	0.0008	0.0014	0.0000	0.0010
0.0000	0.0049	0.0000	0.0000	0.0000	0.0058	0.0043	0.0000	0.0054
0.0076	0.0019	0.0002	0.0078	0.0014	0.0080	0.0074	0.0023	0.0056
0.0000	0.0000	0.0000	0.0000	0.0000	0.0000	0.0000	0.0000	0.0014
0.0000	0.0000	0.0000	0.0000	0.0000	0.0000	0.0019	0.0000	0.0033
0.0025	0.0000	0.0000	0.0000	0.0000	0.0016	0.0000	0.0031	0.0041
0.0023	0.0016	0.0000	0.0006	0.0010	0.0029	0.0014	0.0016	0.0029
0.0000	0.0000	0.0000	0.0016	0.0000	0.0000	0.0006	0.0000	0.0031
0.0198	0.0126	0.0097	0.0389	0.0191	0.0187	0.0335	0.0181	0.0311
0.0027	0.0012	0.0000	0.0000	0.0035	0.0012	0.0000	0.0000	0.0029
0.0000	0.0000	0.0000	0.0000	0.0000	0.0000	0.0000	0.0000	0.0000
0.0099	0.0235	0.0146	0.0313	0.0286	0.0107	0.0111	0.0128	0.0193
0.0000	0.0000	0.0000	0.0327	0.0418	0.0407	0.0327	0.0000	0.0000
0.0035	0.0019	0.0021	0.0043	0.0000	0.0014	0.0029	0.0025	0.0039
0.0039	0.0054	0.0039	0.0035	0.0035	0.0045	0.0041	0.0058	0.0113
0.0231	0.0111	0.0066	0.0066	0.0117	0.0068	0.0080	0.0084	0.0113
0.0008	0.0029	0.0000	0.0006	0.0000	0.0031	0.0076	0.0010	0.0023
0.0047	0.0027	0.0014	0.0000	0.0078	0.0033	0.0000	0.0082	0.0099
0.0000	0.0008	0.0000	0.0000	0.0000	0.0000	0.0000	0.0004	0.0014
0.0016	0.0000	0.0000	0.0000	0.0000	0.0123	0.0097	0.0002	0.0006

	3G D70						
M7	M1	M2	M3	M4	M5	M6	M7
0.0000	0.0043	0.0008	0.0000	0.0000	0.0000	0.0000	0.0000
0.0000	0.0000	0.0000	0.0000	0.0000	0.0000	0.0000	0.0000
0.0021	0.0000	0.0000	0.0000	0.0000	0.0031	0.0016	0.0016
0.0210	0.0138	0.0158	0.0099	0.0072	0.0204	0.0150	0.0245
0.0000	0.0000	0.0000	0.0000	0.0000	0.0000	0.0000	0.0000
0.0455	0.0447	0.0350	0.0391	0.0597	0.0529	0.0580	0.0510
0.0128	0.0105	0.0080	0.0117	0.0146	0.0095	0.0103	0.0084
0.2655	0.2161	0.1772	0.1992	0.2708	0.2336	0.2223	0.2428
0.0080	0.0095	0.0070	0.0078	0.0130	0.0000	0.0072	0.0000
0.0027	0.0041	0.0031	0.0045	0.0016	0.0025	0.0043	0.0016
0.0000	0.0000	0.0000	0.0000	0.0000	0.0144	0.0103	0.0043
0.0000	0.0000	0.0000	0.0000	0.0000	0.0086	0.0134	0.0119
0.3694	0.2797	0.1297	0.1887	0.2601	0.1690	0.1237	0.1978
0.0010	0.0006	0.0000	0.0000	0.0064	0.0010	0.0010	0.0000
0.0109	0.0000	0.0000	0.0000	0.0000	0.0000	0.0169	0.0126
0.0000	0.0000	0.0000	0.0000	0.0000	0.0000	0.0000	0.0000
0.0000	0.0027	0.0006	0.0019	0.0000	0.0049	0.0010	0.0043
0.0000	0.0008	0.0023	0.0025	0.0004	0.0023	0.0000	0.0029
0.0173	0.0383	0.0704	0.1253	0.0568	0.0609	0.0718	0.0957
0.0054	0.0146	0.0226	0.0146	0.0101	0.0144	0.0152	0.0214
0.0000	0.0019	0.0027	0.0019	0.0000	0.0027	0.0045	0.0000
0.0000	0.0033	0.0082	0.0033	0.0000	0.0000	0.0035	0.0000
0.0021	0.0072	0.0109	0.0029	0.0000	0.0023	0.0047	0.0049
0.0117	0.0169	0.0298	0.0128	0.0183	0.0233	0.0140	0.0198
0.0014	0.0078	0.0031	0.0066	0.0000	0.0000	0.0000	0.0021
0.0368	0.0887	0.0856	0.1286	0.0681	0.1087	0.0858	0.0759
0.0000	0.0000	0.0449	0.0000	0.0000	0.0000	0.0000	0.0000
0.0000	0.0173	0.0208	0.0146	0.0101	0.0095	0.0146	0.0189
0.0047	0.0138	0.0185	0.0422	0.0093	0.0321	0.0214	0.0261
0.0000	0.0060	0.0093	0.0000	0.0000	0.0054	0.0000	0.0000
0.0000	0.0150	0.0342	0.0126	0.0107	0.0181	0.0068	0.0123
0.0000	0.0016	0.0051	0.0000	0.0000	0.0045	0.0010	0.0016
0.0000	0.0000	0.0000	0.0000	0.0000	0.0000	0.0000	0.0000
0.0076	0.0196	0.0407	0.0278	0.0224	0.0249	0.0255	0.0132
0.0062	0.0000	0.0043	0.0000	0.0000	0.0023	0.0049	0.0000
0.0019	0.0027	0.0062	0.0037	0.0014	0.0000	0.0025	0.0000
0.0000	0.0000	0.0000	0.0000	0.0000	0.0008	0.0019	0.0000
0.0029	0.0000	0.0235	0.0074	0.0049	0.0037	0.0150	0.0025
0.0039	0.0095	0.0136	0.0078	0.0078	0.0097	0.0138	0.0086
0.0000	0.0000	0.0000	0.0058	0.0000	0.0082	0.0031	0.0000
0.0008	0.0010	0.0000	0.0000	0.0031	0.0000	0.0029	0.0000
0.0023	0.0016	0.0033	0.0045	0.0014	0.0037	0.0080	0.0000
0.0066	0.0006	0.0016	0.0006	0.0014	0.0012	0.0021	0.0008
0.0012	0.0016	0.0000	0.0000	0.0000	0.0000	0.0025	0.0000
0.0401	0.0226	0.0051	0.0000	0.0208	0.0121	0.0303	0.0210
0.0000	0.0023	0.0181	0.0033	0.0016	0.0107	0.0111	0.0035
0.0000	0.0000	0.0000	0.0000	0.0000	0.0000	0.0000	0.0000
0.0068	0.0107	0.0000	0.0037	0.0121	0.0000	0.0070	0.0041
0.0000	0.0142	0.0103	0.0185	0.0152	0.0000	0.0000	0.0000
0.0074	0.0014	0.0000	0.0021	0.0035	0.0010	0.0033	0.0049
0.0080	0.0049	0.0027	0.0051	0.0047	0.0113	0.0019	0.0043
0.0049	0.0158	0.0130	0.0070	0.0130	0.0315	0.0072	0.0191
0.0012	0.0021	0.0084	0.0031	0.0084	0.0043	0.0105	0.0012
0.0103	0.0113	0.0031	0.0006	0.0016	0.0002	0.0099	0.0058
0.0002	0.0000	0.0000	0.0000	0.0004	0.0000	0.0000	0.0000
0.0000	0.0000	0.0000	0.0033	0.0047	0.0000	0.0000	0.0016

Table S2. Genus-level relative abundance of bacterial taxa in recipient mice receiving the antibiotic-disrupted microbiota. Related to Figure 7. Phylogenetic classification and relative abundance (genus level) of operational taxonomic units (OTUs) in donor and recipients of the single gavage (1G) and multiple gavage (3G) groups for the antibiotic-disrupted microbiota.

**Phylogenetic classification**			
**Phylum**	**Class**	**Family**	**Order**
Bacteroidetes	Bacteroidia	Bacteroidales	Muribaculaceae
	Bacteroidia	Bacteroidales	Rikenellaceae
Firmicutes	Bacilli	Bacillales	Staphylococcaceae
	Clostridia	Clostridiales	Clostridiales vadinBB60 group
	Clostridia	Clostridiales	Clostridiales vadinBB60 group
	Clostridia	Clostridiales	Lachnospiraceae
	Clostridia	Clostridiales	Lachnospiraceae
	Clostridia	Clostridiales	Lachnospiraceae
	Clostridia	Clostridiales	Lachnospiraceae
	Clostridia	Clostridiales	Lachnospiraceae
	Clostridia	Clostridiales	Lachnospiraceae
	Clostridia	Clostridiales	Lachnospiraceae
	Clostridia	Clostridiales	Peptostreptococcaceae
	Clostridia	Clostridiales	Ruminococcaceae
	Clostridia	Clostridiales	Ruminococcaceae
	Clostridia	Clostridiales	Ruminococcaceae
	Clostridia	Clostridiales	Ruminococcaceae
	Clostridia	Clostridiales	Ruminococcaceae
	Clostridia	Clostridiales	Ruminococcaceae
	Clostridia	Clostridiales	Ruminococcaceae
Tenericutes	Mollicutes	Anaeroplasmatales	Anaeroplasmataceae
Verrucomicrobia	Verrucomicrobiae	Verrucomicrobiales	Akkermansiaceae

* Sequence variants present in donor taxa at a relative abundance of ≥ 0.0004

	Donor			
**Genus**	**1**	**2**	**3**	**pooled**
uncultured	0.1653	0.1614	0.1807	0.2225
Alistipes	0.0039	0.0037	0.0027	0.0049
Staphylococcus	0.0000	0.0010	0.0000	0.0000
ambiguous taxa	0.0000	0.0021	0.0000	0.0000
unknown taxa	0.0023	0.0000	0.0039	0.0039
Acetatifactor	0.0144	0.0113	0.0076	0.0134
Anaerostipes	0.0134	0.0165	0.0091	0.0208
Blautia	0.4865	0.4845	0.4742	0.3756
Lachnoclostridium	0.0060	0.0080	0.0058	0.0070
Lachnospiraceae UCG-006	0.0029	0.0054	0.0035	0.0058
Eubacterium xylanophilum group	0.0154	0.0152	0.0097	0.0179
unknown taxa	0.0379	0.0399	0.0325	0.0403
Clostridioides	0.0000	0.0019	0.0000	0.0016
Intestinimonas	0.0000	0.0000	0.0000	0.0019
Oscillibacter	0.0000	0.0006	0.0000	0.0000
Ruminiclostridium	0.0121	0.0148	0.0181	0.0140
Ruminiclostridium 5	0.0086	0.0119	0.0169	0.0148
Ruminiclostridium 6	0.0074	0.0105	0.0086	0.0086
Ruminiclostridium 9	0.0148	0.0117	0.0128	0.0123
unknown taxa	0.0284	0.0241	0.0140	0.0202
Anaeroplasma	0.0049	0.0054	0.0049	0.0039
Akkermansia	0.1673	0.1618	0.1846	0.2046

Antibiotic-disrupted microbiota, single gavage (1G)

1G D4

M1	M2	M3	M4	M5	M6	M7
0.2190	0.1967	0.2163	0.2054	0.2221	0.1967	0.2027
0.0000	0.0000	0.0000	0.0000	0.0000	0.0000	0.0000
0.0000	0.0000	0.0000	0.0000	0.0000	0.0000	0.0000
0.0000	0.0000	0.0000	0.0000	0.0000	0.0000	0.0000
0.0000	0.0000	0.0000	0.0000	0.0000	0.0000	0.0000
0.0000	0.0000	0.0000	0.0000	0.0000	0.0000	0.0000
0.0455	0.0410	0.0638	0.0463	0.0383	0.0498	0.0430
0.5131	0.5987	0.4143	0.5789	0.5390	0.5355	0.6211
0.0000	0.0000	0.0000	0.0000	0.0000	0.0000	0.0000
0.0000	0.0000	0.0000	0.0000	0.0000	0.0000	0.0000
0.0000	0.0000	0.0000	0.0000	0.0000	0.0000	0.0000
0.0000	0.0000	0.0000	0.0000	0.0000	0.0000	0.0000
0.0027	0.0049	0.0060	0.0084	0.0123	0.0070	0.0047
0.0000	0.0000	0.0000	0.0000	0.0000	0.0000	0.0000
0.0000	0.0000	0.0000	0.0000	0.0000	0.0000	0.0000
0.0000	0.0000	0.0000	0.0000	0.0000	0.0000	0.0000
0.0000	0.0000	0.0000	0.0000	0.0000	0.0000	0.0000
0.0000	0.0000	0.0000	0.0000	0.0000	0.0000	0.0000
0.0000	0.0000	0.0000	0.0000	0.0000	0.0000	0.0000
0.0000	0.0000	0.0000	0.0000	0.0000	0.0000	0.0000
0.0000	0.0000	0.0000	0.0000	0.0000	0.0000	0.0000
0.2005	0.1420	0.2782	0.1459	0.1756	0.1961	0.1126

1G D7						
M1	M2	M3	M4	M5	M6	M7
0.2072	0.2027	0.1881	0.2068	0.2157	0.2276	0.1784
0.0000	0.0000	0.0000	0.0000	0.0000	0.0000	0.0000
0.0000	0.0000	0.0000	0.0000	0.0000	0.0000	0.0000
0.0000	0.0000	0.0000	0.0000	0.0000	0.0000	0.0000
0.0000	0.0000	0.0000	0.0000	0.0000	0.0000	0.0000
0.0000	0.0000	0.0000	0.0000	0.0000	0.0000	0.0000
0.0286	0.0239	0.0307	0.0290	0.0325	0.0313	0.0307
0.5931	0.5721	0.5324	0.5987	0.5581	0.4941	0.6036
0.0000	0.0000	0.0000	0.0000	0.0000	0.0000	0.0000
0.0000	0.0000	0.0000	0.0000	0.0000	0.0000	0.0000
0.0000	0.0000	0.0000	0.0000	0.0000	0.0000	0.0000
0.0000	0.0000	0.0000	0.0000	0.0000	0.0000	0.0000
0.0056	0.0117	0.0037	0.0021	0.0035	0.0045	0.0045
0.0000	0.0000	0.0000	0.0000	0.0000	0.0000	0.0000
0.0000	0.0000	0.0000	0.0000	0.0000	0.0000	0.0000
0.0000	0.0000	0.0000	0.0000	0.0000	0.0000	0.0000
0.0000	0.0000	0.0000	0.0000	0.0000	0.0000	0.0000
0.0000	0.0000	0.0000	0.0000	0.0000	0.0000	0.0000
0.0000	0.0000	0.0000	0.0000	0.0000	0.0000	0.0000
0.0000	0.0000	0.0000	0.0000	0.0000	0.0000	0.0000
0.0000	0.0000	0.0000	0.0000	0.0000	0.0000	0.0000
0.1537	0.1762	0.2369	0.1492	0.1772	0.2356	0.1686

1G D14						
M1	M2	M3	M4	M5	M6	M7
0.1900	0.1856	0.1875	0.1970	0.2130	0.1836	0.2229
0.0000	0.0000	0.0000	0.0000	0.0000	0.0000	0.0000
0.0000	0.0000	0.0000	0.0000	0.0000	0.0000	0.0000
0.0000	0.0000	0.0000	0.0000	0.0000	0.0000	0.0000
0.0000	0.0000	0.0000	0.0000	0.0000	0.0000	0.0000
0.0000	0.0000	0.0000	0.0000	0.0000	0.0000	0.0000
0.0356	0.0356	0.0294	0.0204	0.0321	0.0286	0.0556
0.5835	0.5264	0.6100	0.5703	0.5048	0.5394	0.4591
0.0000	0.0000	0.0000	0.0000	0.0000	0.0000	0.0000
0.0000	0.0000	0.0000	0.0000	0.0000	0.0000	0.0000
0.0000	0.0000	0.0000	0.0000	0.0000	0.0000	0.0000
0.0000	0.0000	0.0000	0.0000	0.0000	0.0000	0.0000
0.0016	0.0056	0.0060	0.0029	0.0027	0.0031	0.0016
0.0000	0.0000	0.0000	0.0000	0.0000	0.0000	0.0000
0.0000	0.0000	0.0000	0.0000	0.0000	0.0000	0.0000
0.0000	0.0000	0.0000	0.0000	0.0000	0.0000	0.0000
0.0000	0.0000	0.0000	0.0000	0.0000	0.0000	0.0000
0.0000	0.0000	0.0000	0.0000	0.0000	0.0000	0.0000
0.0000	0.0000	0.0000	0.0000	0.0000	0.0000	0.0000
0.0000	0.0000	0.0000	0.0000	0.0000	0.0000	0.0000
0.0000	0.0000	0.0000	0.0000	0.0000	0.0000	0.0000
0.1764	0.2317	0.1519	0.1943	0.2377	0.2340	0.2463

1G D21						
M1	M2	M3	M4	M5	M6	M7
0.2157	0.2025	0.2264	0.1624	0.2348	0.2120	0.2149
0.0000	0.0000	0.0000	0.0000	0.0000	0.0000	0.0000
0.0000	0.0000	0.0000	0.0000	0.0000	0.0000	0.0000
0.0000	0.0000	0.0000	0.0000	0.0000	0.0000	0.0000
0.0000	0.0000	0.0000	0.0000	0.0000	0.0000	0.0000
0.0000	0.0000	0.0000	0.0000	0.0000	0.0000	0.0000
0.0276	0.0391	0.0366	0.0346	0.0212	0.0438	0.0307
0.4176	0.5320	0.4040	0.5507	0.5061	0.5400	0.5199
0.0000	0.0000	0.0000	0.0000	0.0000	0.0000	0.0000
0.0000	0.0000	0.0000	0.0000	0.0000	0.0000	0.0000
0.0000	0.0000	0.0000	0.0000	0.0000	0.0000	0.0000
0.0000	0.0000	0.0056	0.0021	0.0000	0.0000	0.0000
0.0179	0.0076	0.0414	0.0095	0.0049	0.0021	0.0150
0.0000	0.0000	0.0000	0.0000	0.0000	0.0000	0.0000
0.0006	0.0000	0.0010	0.0000	0.0000	0.0000	0.0014
0.0000	0.0000	0.0000	0.0000	0.0000	0.0000	0.0000
0.0000	0.0000	0.0000	0.0000	0.0000	0.0000	0.0000
0.0000	0.0000	0.0000	0.0000	0.0000	0.0000	0.0000
0.0000	0.0000	0.0000	0.0000	0.0000	0.0000	0.0000
0.0000	0.0000	0.0000	0.0000	0.0000	0.0000	0.0000
0.0000	0.0000	0.0000	0.0000	0.0000	0.0000	0.0000
0.3120	0.2116	0.2729	0.2342	0.2233	0.1893	0.2070

1G D28						
M1	M2	M3	M4	M5	M6	M7
0.1653	0.1856	0.2066	0.2377	0.2324	0.1848	0.2025
0.0000	0.0000	0.0000	0.0000	0.0000	0.0000	0.0000
0.0000	0.0000	0.0000	0.0000	0.0000	0.0000	0.0000
0.0000	0.0000	0.0000	0.0000	0.0000	0.0000	0.0000
0.0000	0.0000	0.0000	0.0000	0.0000	0.0000	0.0000
0.0000	0.0000	0.0000	0.0000	0.0000	0.0000	0.0000
0.0296	0.0389	0.0373	0.0432	0.0457	0.0436	0.0358
0.5518	0.5232	0.5326	0.4310	0.5174	0.5390	0.5653
0.0000	0.0000	0.0000	0.0000	0.0000	0.0000	0.0000
0.0000	0.0000	0.0000	0.0000	0.0000	0.0000	0.0000
0.0000	0.0000	0.0000	0.0000	0.0000	0.0000	0.0000
0.0000	0.0000	0.0278	0.0185	0.0000	0.0000	0.0000
0.0097	0.0165	0.0021	0.0037	0.0045	0.0014	0.0060
0.0000	0.0000	0.0000	0.0000	0.0000	0.0000	0.0000
0.0014	0.0000	0.0037	0.0000	0.0000	0.0008	0.0014
0.0000	0.0000	0.0000	0.0000	0.0000	0.0000	0.0000
0.0000	0.0000	0.0000	0.0000	0.0000	0.0000	0.0000
0.0000	0.0000	0.0000	0.0000	0.0000	0.0000	0.0000
0.0000	0.0000	0.0000	0.0000	0.0000	0.0000	0.0000
0.0000	0.0000	0.0000	0.0000	0.0000	0.0000	0.0000
0.0000	0.0000	0.0000	0.0000	0.0000	0.0000	0.0000
0.2356	0.2243	0.1805	0.2533	0.1869	0.2239	0.1778

1G D35						
M1	M2	M3	M4	M5	M6	M7
0.3147	0.3056	0.3381	0.3363	0.3420	0.3126	0.2727
0.0000	0.0000	0.0000	0.0000	0.0000	0.0000	0.0000
0.0000	0.0000	0.0000	0.0000	0.0000	0.0000	0.0000
0.0000	0.0000	0.0000	0.0000	0.0000	0.0000	0.0000
0.0000	0.0000	0.0000	0.0000	0.0000	0.0000	0.0000
0.0000	0.0000	0.0000	0.0000	0.0000	0.0000	0.0000
0.0130	0.0161	0.0202	0.0187	0.0163	0.0216	0.0231
0.4083	0.4980	0.3731	0.3789	0.4330	0.4834	0.5419
0.0000	0.0000	0.0000	0.0000	0.0000	0.0000	0.0000
0.0000	0.0000	0.0000	0.0000	0.0000	0.0000	0.0000
0.0000	0.0000	0.0000	0.0000	0.0000	0.0000	0.0000
0.0000	0.0000	0.0119	0.0076	0.0000	0.0000	0.0000
0.0072	0.0082	0.0088	0.0146	0.0115	0.0093	0.0099
0.0000	0.0000	0.0000	0.0000	0.0000	0.0000	0.0000
0.0000	0.0000	0.0016	0.0000	0.0010	0.0000	0.0016
0.0000	0.0000	0.0000	0.0000	0.0000	0.0000	0.0000
0.0000	0.0000	0.0000	0.0000	0.0000	0.0000	0.0000
0.0000	0.0000	0.0000	0.0000	0.0000	0.0000	0.0000
0.0000	0.0000	0.0000	0.0000	0.0000	0.0000	0.0000
0.0000	0.0000	0.0000	0.0000	0.0000	0.0000	0.0000
0.0000	0.0000	0.0000	0.0000	0.0000	0.0000	0.0000
0.2350	0.1554	0.2282	0.2206	0.1743	0.1581	0.1371

1G D42						
M1	M2	M3	M4	M5	M6	M7
0.3398	0.3143	0.3340	0.3591	0.3849	0.3492	0.3511
0.0000	0.0000	0.0000	0.0000	0.0000	0.0000	0.0000
0.0000	0.0000	0.0000	0.0000	0.0000	0.0000	0.0000
0.0000	0.0000	0.0000	0.0000	0.0000	0.0000	0.0000
0.0000	0.0000	0.0000	0.0000	0.0000	0.0000	0.0000
0.0000	0.0000	0.0000	0.0000	0.0000	0.0000	0.0000
0.0237	0.0191	0.0228	0.0239	0.0105	0.0161	0.0185
0.4215	0.4583	0.4118	0.3947	0.3402	0.4145	0.3811
0.0000	0.0000	0.0000	0.0000	0.0000	0.0000	0.0000
0.0000	0.0000	0.0000	0.0000	0.0000	0.0000	0.0000
0.0000	0.0000	0.0000	0.0000	0.0000	0.0000	0.0000
0.0000	0.0000	0.0097	0.0070	0.0196	0.0062	0.0309
0.0191	0.0064	0.0082	0.0058	0.0076	0.0101	0.0138
0.0000	0.0000	0.0000	0.0000	0.0000	0.0000	0.0000
0.0023	0.0035	0.0004	0.0000	0.0000	0.0004	0.0000
0.0000	0.0000	0.0000	0.0000	0.0000	0.0000	0.0000
0.0000	0.0000	0.0000	0.0000	0.0000	0.0000	0.0000
0.0000	0.0000	0.0000	0.0000	0.0000	0.0000	0.0000
0.0000	0.0000	0.0000	0.0000	0.0000	0.0000	0.0000
0.0000	0.0000	0.0000	0.0000	0.0000	0.0000	0.0000
0.0000	0.0000	0.0000	0.0000	0.0000	0.0000	0.0000
0.1758	0.1834	0.1963	0.1883	0.2171	0.1858	0.1813

1G D49						
M1	M2	M3	M4	M5	M6	M7
0.3221	0.3307	0.3532	0.3377	0.3799	0.3278	0.3616
0.0000	0.0000	0.0000	0.0000	0.0000	0.0000	0.0000
0.0000	0.0000	0.0000	0.0000	0.0000	0.0000	0.0000
0.0000	0.0000	0.0000	0.0000	0.0000	0.0000	0.0000
0.0000	0.0000	0.0000	0.0000	0.0000	0.0000	0.0000
0.0000	0.0000	0.0000	0.0000	0.0000	0.0000	0.0000
0.0212	0.0261	0.0253	0.0158	0.0233	0.0193	0.0140
0.4696	0.4738	0.3719	0.3970	0.3945	0.4493	0.4126
0.0000	0.0000	0.0000	0.0000	0.0000	0.0000	0.0000
0.0000	0.0000	0.0000	0.0000	0.0000	0.0000	0.0000
0.0000	0.0000	0.0000	0.0000	0.0000	0.0000	0.0000
0.0000	0.0000	0.0222	0.0249	0.0346	0.0317	0.0249
0.0064	0.0064	0.0082	0.0185	0.0119	0.0058	0.0086
0.0000	0.0000	0.0000	0.0000	0.0000	0.0000	0.0000
0.0000	0.0000	0.0054	0.0014	0.0064	0.0014	0.0000
0.0000	0.0000	0.0000	0.0000	0.0000	0.0000	0.0000
0.0000	0.0000	0.0000	0.0000	0.0000	0.0000	0.0000
0.0000	0.0000	0.0000	0.0000	0.0000	0.0000	0.0000
0.0000	0.0000	0.0000	0.0000	0.0000	0.0000	0.0000
0.0000	0.0000	0.0000	0.0000	0.0000	0.0000	0.0000
0.0000	0.0000	0.0000	0.0000	0.0000	0.0000	0.0000
0.1630	0.1474	0.1974	0.1873	0.1297	0.1471	0.1593

1G D56						
M1	M2	M3	M4	M5	M6	M7
0.3579	0.2871	0.3659	0.3412	0.3470	0.3425	0.3709
0.0000	0.0000	0.0000	0.0000	0.0000	0.0000	0.0000
0.0000	0.0000	0.0000	0.0000	0.0000	0.0000	0.0000
0.0000	0.0000	0.0000	0.0000	0.0000	0.0000	0.0000
0.0000	0.0000	0.0000	0.0000	0.0000	0.0000	0.0000
0.0000	0.0000	0.0000	0.0000	0.0000	0.0000	0.0000
0.0173	0.0202	0.0216	0.0111	0.0150	0.0095	0.0161
0.4089	0.5085	0.4178	0.3583	0.4324	0.3624	0.3892
0.0000	0.0000	0.0000	0.0000	0.0000	0.0000	0.0000
0.0000	0.0000	0.0000	0.0000	0.0000	0.0000	0.0000
0.0000	0.0000	0.0000	0.0000	0.0000	0.0000	0.0000
0.0000	0.0000	0.0177	0.0115	0.0105	0.0132	0.0105
0.0084	0.0099	0.0049	0.0076	0.0051	0.0068	0.0115
0.0000	0.0000	0.0000	0.0000	0.0000	0.0000	0.0000
0.0000	0.0012	0.0000	0.0000	0.0010	0.0000	0.0000
0.0000	0.0000	0.0000	0.0000	0.0000	0.0000	0.0000
0.0000	0.0000	0.0000	0.0000	0.0000	0.0000	0.0000
0.0000	0.0000	0.0000	0.0000	0.0000	0.0000	0.0000
0.0000	0.0000	0.0000	0.0000	0.0000	0.0000	0.0000
0.0000	0.0000	0.0000	0.0000	0.0000	0.0000	0.0000
0.0000	0.0000	0.0000	0.0000	0.0000	0.0000	0.0000
0.1935	0.1576	0.1562	0.2507	0.1718	0.2449	0.1863

1G D63						
M1	M2	M3	M4	M5	M6	M7
0.2931	0.3112	0.3501	0.3299	0.3667	0.3248	0.3373
0.0000	0.0000	0.0000	0.0000	0.0000	0.0000	0.0000
0.0000	0.0000	0.0000	0.0000	0.0000	0.0000	0.0000
0.0000	0.0000	0.0000	0.0000	0.0000	0.0000	0.0000
0.0000	0.0000	0.0000	0.0000	0.0000	0.0000	0.0000
0.0000	0.0000	0.0000	0.0000	0.0000	0.0000	0.0000
0.0173	0.0191	0.0163	0.0200	0.0181	0.0130	0.0152
0.4773	0.4933	0.4118	0.4235	0.3935	0.4633	0.3991
0.0000	0.0000	0.0000	0.0000	0.0000	0.0000	0.0000
0.0000	0.0000	0.0000	0.0000	0.0000	0.0000	0.0000
0.0000	0.0000	0.0000	0.0000	0.0000	0.0000	0.0000
0.0000	0.0000	0.0115	0.0064	0.0093	0.0082	0.0115
0.0088	0.0058	0.0064	0.0047	0.0072	0.0103	0.0154
0.0000	0.0000	0.0000	0.0000	0.0000	0.0000	0.0000
0.0008	0.0000	0.0037	0.0000	0.0023	0.0000	0.0000
0.0000	0.0000	0.0000	0.0000	0.0000	0.0000	0.0000
0.0000	0.0000	0.0000	0.0000	0.0000	0.0000	0.0000
0.0000	0.0000	0.0000	0.0000	0.0000	0.0000	0.0000
0.0000	0.0000	0.0000	0.0000	0.0000	0.0000	0.0000
0.0000	0.0000	0.0000	0.0000	0.0000	0.0000	0.0000
0.0000	0.0000	0.0000	0.0000	0.0000	0.0000	0.0000
0.1863	0.1560	0.1832	0.1992	0.1842	0.1618	0.2070

1G D70

M1	M2	M3	M4	M5	M6	M7
0.3772	0.2982	0.3542	0.3460	0.3908	0.3305	0.3579
0.0000	0.0000	0.0000	0.0000	0.0000	0.0000	0.0000
0.0000	0.0000	0.0000	0.0000	0.0000	0.0000	0.0000
0.0000	0.0000	0.0000	0.0000	0.0000	0.0000	0.0000
0.0000	0.0000	0.0000	0.0000	0.0000	0.0000	0.0000
0.0000	0.0000	0.0000	0.0000	0.0000	0.0000	0.0000
0.0189	0.0200	0.0185	0.0134	0.0169	0.0156	0.0193
0.3624	0.4505	0.3682	0.3707	0.3118	0.4396	0.4098
0.0000	0.0000	0.0000	0.0000	0.0000	0.0000	0.0000
0.0000	0.0000	0.0000	0.0000	0.0000	0.0000	0.0000
0.0000	0.0000	0.0000	0.0000	0.0000	0.0000	0.0000
0.0000	0.0000	0.0134	0.0535	0.0091	0.0103	0.0103
0.0101	0.0150	0.0142	0.0099	0.0144	0.0097	0.0080
0.0000	0.0000	0.0000	0.0000	0.0000	0.0000	0.0000
0.0000	0.0025	0.0072	0.0021	0.0027	0.0000	0.0000
0.0000	0.0000	0.0000	0.0000	0.0000	0.0000	0.0000
0.0000	0.0000	0.0000	0.0000	0.0000	0.0000	0.0000
0.0000	0.0000	0.0000	0.0000	0.0000	0.0000	0.0000
0.0000	0.0000	0.0000	0.0000	0.0000	0.0000	0.0000
0.0000	0.0000	0.0000	0.0000	0.0000	0.0000	0.0000
0.0000	0.0000	0.0000	0.0000	0.0000	0.0000	0.0000
0.2188	0.1959	0.2085	0.1858	0.2383	0.1741	0.1795

Antibiotic-disrupted microbiota, multiple gavage (3G)						
3G D4						
M1	M2	M3	M4	M5	M6	M7
0.1943	0.2101	0.2284	0.2066	0.2169	0.2122	0.2056
0.0000	0.0000	0.0000	0.0000	0.0000	0.0000	0.0000
0.0000	0.0000	0.0000	0.0000	0.0000	0.0000	0.0000
0.0000	0.0000	0.0000	0.0000	0.0000	0.0000	0.0000
0.0000	0.0000	0.0000	0.0000	0.0000	0.0000	0.0000
0.0000	0.0000	0.0000	0.0000	0.0000	0.0000	0.0000
0.0401	0.0440	0.0331	0.0459	0.0373	0.0237	0.0463
0.5194	0.5332	0.5203	0.5435	0.5215	0.4746	0.5596
0.0000	0.0000	0.0000	0.0000	0.0000	0.0000	0.0000
0.0000	0.0000	0.0000	0.0000	0.0000	0.0000	0.0000
0.0000	0.0000	0.0000	0.0000	0.0000	0.0000	0.0000
0.0000	0.0000	0.0000	0.0000	0.0000	0.0000	0.0000
0.0043	0.0043	0.0051	0.0074	0.0233	0.0282	0.0074
0.0000	0.0000	0.0000	0.0000	0.0000	0.0000	0.0000
0.0000	0.0000	0.0000	0.0000	0.0000	0.0000	0.0000
0.0000	0.0000	0.0000	0.0000	0.0000	0.0000	0.0000
0.0000	0.0000	0.0000	0.0000	0.0000	0.0000	0.0000
0.0000	0.0000	0.0000	0.0000	0.0000	0.0000	0.0000
0.0000	0.0000	0.0000	0.0000	0.0000	0.0000	0.0000
0.0000	0.0000	0.0000	0.0000	0.0000	0.0000	0.0000
0.0000	0.0000	0.0000	0.0000	0.0000	0.0000	0.0000
0.2278	0.1957	0.1982	0.1819	0.1825	0.2431	0.1657

3G D7						
M1	M2	M3	M4	M5	M6	M7
0.1735	0.1937	0.1817	0.2299	0.1970	0.1918	0.2040
0.0000	0.0000	0.0000	0.0000	0.0000	0.0000	0.0000
0.0000	0.0000	0.0000	0.0000	0.0000	0.0000	0.0000
0.0000	0.0000	0.0000	0.0000	0.0000	0.0000	0.0000
0.0000	0.0000	0.0000	0.0000	0.0000	0.0000	0.0000
0.0000	0.0000	0.0000	0.0000	0.0000	0.0000	0.0000
0.0335	0.0315	0.0212	0.0407	0.0298	0.0280	0.0241
0.6057	0.5686	0.5843	0.4915	0.5958	0.5719	0.5623
0.0000	0.0000	0.0000	0.0000	0.0000	0.0000	0.0000
0.0000	0.0000	0.0000	0.0000	0.0000	0.0000	0.0000
0.0000	0.0000	0.0000	0.0000	0.0000	0.0000	0.0000
0.0000	0.0000	0.0000	0.0000	0.0000	0.0000	0.0000
0.0023	0.0023	0.0023	0.0025	0.0031	0.0074	0.0039
0.0000	0.0000	0.0000	0.0000	0.0000	0.0000	0.0000
0.0000	0.0000	0.0000	0.0000	0.0000	0.0000	0.0000
0.0000	0.0000	0.0000	0.0000	0.0000	0.0000	0.0000
0.0000	0.0000	0.0000	0.0000	0.0000	0.0000	0.0000
0.0000	0.0000	0.0000	0.0000	0.0000	0.0000	0.0000
0.0000	0.0000	0.0000	0.0000	0.0000	0.0000	0.0000
0.0000	0.0000	0.0000	0.0000	0.0000	0.0000	0.0000
0.0000	0.0000	0.0000	0.0000	0.0000	0.0000	0.0000
0.1675	0.1914	0.1819	0.2040	0.1603	0.1893	0.1955

3G D14						
M1	M2	M3	M4	M5	M6	M7
0.1807	0.1504	0.1799	0.1941	0.1924	0.2021	0.2105
0.0000	0.0000	0.0000	0.0000	0.0000	0.0000	0.0000
0.0000	0.0000	0.0000	0.0000	0.0000	0.0000	0.0000
0.0000	0.0000	0.0000	0.0000	0.0000	0.0000	0.0000
0.0000	0.0000	0.0000	0.0000	0.0000	0.0000	0.0000
0.0000	0.0000	0.0000	0.0000	0.0000	0.0000	0.0000
0.0257	0.0385	0.0375	0.0370	0.0364	0.0438	0.0389
0.5900	0.6324	0.5425	0.5129	0.5629	0.5295	0.4855
0.0000	0.0000	0.0000	0.0000	0.0000	0.0000	0.0000
0.0000	0.0000	0.0000	0.0000	0.0000	0.0000	0.0000
0.0000	0.0000	0.0000	0.0000	0.0000	0.0000	0.0000
0.0025	0.0000	0.0000	0.0000	0.0000	0.0000	0.0000
0.0074	0.0016	0.0012	0.0080	0.0021	0.0023	0.0008
0.0000	0.0000	0.0037	0.0000	0.0000	0.0000	0.0000
0.0000	0.0000	0.0000	0.0000	0.0000	0.0000	0.0000
0.0000	0.0000	0.0000	0.0000	0.0000	0.0000	0.0000
0.0000	0.0000	0.0000	0.0000	0.0000	0.0000	0.0000
0.0000	0.0000	0.0000	0.0000	0.0000	0.0000	0.0000
0.0000	0.0000	0.0000	0.0000	0.0000	0.0000	0.0000
0.0000	0.0000	0.0054	0.0000	0.0000	0.0000	0.0000
0.0000	0.0000	0.0000	0.0000	0.0000	0.0006	0.0000
0.1825	0.1702	0.1994	0.2165	0.1961	0.2128	0.2536

3G D21						
M1	M2	M3	M4	M5	M6	M7
0.2418	0.2562	0.2684	0.2361	0.2085	0.2251	0.1879
0.0000	0.0000	0.0000	0.0000	0.0000	0.0000	0.0000
0.0000	0.0000	0.0000	0.0000	0.0000	0.0000	0.0000
0.0000	0.0000	0.0000	0.0000	0.0000	0.0000	0.0000
0.0000	0.0000	0.0000	0.0000	0.0000	0.0000	0.0000
0.0000	0.0000	0.0000	0.0000	0.0000	0.0000	0.0000
0.0364	0.0200	0.0249	0.0218	0.0292	0.0426	0.0428
0.3643	0.3219	0.3534	0.3717	0.4610	0.4725	0.4875
0.0000	0.0000	0.0000	0.0000	0.0000	0.0000	0.0000
0.0000	0.0021	0.0000	0.0000	0.0000	0.0000	0.0000
0.0000	0.0000	0.0000	0.0000	0.0000	0.0000	0.0000
0.0173	0.0206	0.0140	0.0142	0.0000	0.0000	0.0000
0.0068	0.0196	0.0134	0.0126	0.0051	0.0045	0.0025
0.0000	0.0000	0.0029	0.0035	0.0000	0.0000	0.0000
0.0000	0.0000	0.0000	0.0000	0.0035	0.0027	0.0076
0.0000	0.0000	0.0000	0.0000	0.0000	0.0000	0.0000
0.0000	0.0000	0.0000	0.0000	0.0000	0.0000	0.0000
0.0000	0.0000	0.0000	0.0000	0.0000	0.0000	0.0000
0.0000	0.0000	0.0000	0.0000	0.0000	0.0000	0.0000
0.0029	0.0068	0.0033	0.0039	0.0000	0.0000	0.0000
0.0000	0.0000	0.0000	0.0000	0.0115	0.0146	0.0224
0.3229	0.3433	0.2949	0.3079	0.2756	0.2276	0.2437

3G D28						
M1	M2	M3	M4	M5	M6	M7
0.2282	0.2268	0.2385	0.2585	0.1895	0.2081	0.1924
0.0000	0.0000	0.0000	0.0000	0.0000	0.0000	0.0000
0.0000	0.0000	0.0000	0.0000	0.0000	0.0000	0.0000
0.0000	0.0000	0.0000	0.0000	0.0000	0.0000	0.0000
0.0000	0.0000	0.0000	0.0000	0.0000	0.0000	0.0000
0.0000	0.0000	0.0000	0.0000	0.0000	0.0000	0.0000
0.0214	0.0261	0.0231	0.0226	0.0245	0.0391	0.0395
0.4044	0.4534	0.4445	0.4050	0.4604	0.5151	0.5240
0.0000	0.0000	0.0000	0.0000	0.0000	0.0000	0.0000
0.0000	0.0000	0.0000	0.0000	0.0000	0.0000	0.0000
0.0000	0.0000	0.0000	0.0000	0.0000	0.0000	0.0000
0.0163	0.0298	0.0099	0.0095	0.0000	0.0000	0.0000
0.0358	0.0222	0.0123	0.0056	0.0208	0.0049	0.0154
0.0000	0.0000	0.0023	0.0008	0.0000	0.0000	0.0000
0.0000	0.0000	0.0000	0.0000	0.0058	0.0012	0.0031
0.0000	0.0000	0.0000	0.0000	0.0000	0.0000	0.0000
0.0000	0.0000	0.0000	0.0000	0.0000	0.0000	0.0000
0.0000	0.0000	0.0000	0.0000	0.0000	0.0000	0.0000
0.0000	0.0000	0.0000	0.0000	0.0000	0.0000	0.0000
0.0097	0.0056	0.0039	0.0000	0.0000	0.0000	0.0000
0.0000	0.0000	0.0000	0.0000	0.0175	0.0152	0.0128
0.2758	0.2262	0.2359	0.2661	0.2758	0.2091	0.2027

3G D35						
M1	M2	M3	M4	M5	M6	M7
0.3313	0.3727	0.4009	0.3433	0.3136	0.2898	0.2626
0.0000	0.0000	0.0000	0.0000	0.0000	0.0000	0.0000
0.0000	0.0000	0.0000	0.0000	0.0000	0.0000	0.0000
0.0000	0.0000	0.0000	0.0000	0.0000	0.0000	0.0000
0.0000	0.0000	0.0000	0.0000	0.0000	0.0000	0.0000
0.0000	0.0000	0.0000	0.0000	0.0000	0.0000	0.0000
0.0111	0.0257	0.0093	0.0107	0.0111	0.0276	0.0228
0.3795	0.2865	0.3281	0.3789	0.4355	0.4832	0.5441
0.0000	0.0000	0.0000	0.0000	0.0000	0.0000	0.0000
0.0000	0.0000	0.0000	0.0000	0.0000	0.0000	0.0000
0.0000	0.0000	0.0000	0.0000	0.0000	0.0000	0.0000
0.0080	0.0239	0.0091	0.0093	0.0000	0.0000	0.0000
0.0103	0.0329	0.0084	0.0082	0.0086	0.0084	0.0091
0.0000	0.0000	0.0037	0.0058	0.0000	0.0000	0.0000
0.0000	0.0000	0.0002	0.0023	0.0019	0.0033	0.0000
0.0000	0.0000	0.0000	0.0000	0.0000	0.0000	0.0000
0.0000	0.0000	0.0000	0.0000	0.0000	0.0000	0.0000
0.0000	0.0000	0.0000	0.0000	0.0000	0.0000	0.0000
0.0000	0.0000	0.0000	0.0000	0.0000	0.0000	0.0000
0.0086	0.0222	0.0041	0.0023	0.0000	0.0000	0.0000
0.0000	0.0000	0.0000	0.0000	0.0198	0.0202	0.0097
0.2239	0.1945	0.2134	0.2128	0.1937	0.1480	0.1424

3G D42						
M1	M2	M3	M4	M5	M6	M7
0.3630	0.3542	0.2424	0.3614	0.3052	0.3161	0.3085
0.0000	0.0000	0.0000	0.0000	0.0000	0.0000	0.0000
0.0000	0.0000	0.0000	0.0000	0.0000	0.0000	0.0000
0.0000	0.0000	0.0000	0.0000	0.0000	0.0000	0.0000
0.0000	0.0000	0.0000	0.0000	0.0000	0.0000	0.0000
0.0000	0.0000	0.0000	0.0000	0.0000	0.0000	0.0000
0.0113	0.0072	0.0364	0.0132	0.0179	0.0095	0.0222
0.3264	0.3511	0.4612	0.3807	0.4032	0.4242	0.4414
0.0000	0.0000	0.0000	0.0000	0.0000	0.0000	0.0000
0.0000	0.0000	0.0000	0.0000	0.0000	0.0000	0.0000
0.0000	0.0000	0.0000	0.0000	0.0000	0.0000	0.0000
0.0148	0.0113	0.0218	0.0128	0.0000	0.0000	0.0000
0.0000	0.0115	0.0247	0.0115	0.0000	0.0084	0.0068
0.0000	0.0021	0.0000	0.0058	0.0000	0.0000	0.0000
0.0006	0.0016	0.0000	0.0049	0.0029	0.0000	0.0000
0.0000	0.0000	0.0000	0.0000	0.0000	0.0000	0.0000
0.0000	0.0000	0.0000	0.0000	0.0000	0.0000	0.0000
0.0000	0.0000	0.0000	0.0000	0.0000	0.0000	0.0000
0.0000	0.0000	0.0000	0.0000	0.0000	0.0000	0.0000
0.0031	0.0000	0.0000	0.0004	0.0000	0.0000	0.0000
0.0000	0.0000	0.0000	0.0000	0.0161	0.0056	0.0173
0.2474	0.2280	0.1838	0.1846	0.2334	0.2190	0.1916

3G D49						
M1	M2	M3	M4	M5	M6	M7
0.3406	0.3698	0.3433	0.3404	0.3040	0.3110	0.2840
0.0000	0.0000	0.0000	0.0000	0.0000	0.0000	0.0000
0.0000	0.0000	0.0000	0.0000	0.0000	0.0000	0.0000
0.0000	0.0000	0.0000	0.0000	0.0000	0.0000	0.0000
0.0000	0.0000	0.0000	0.0000	0.0000	0.0000	0.0000
0.0000	0.0000	0.0000	0.0000	0.0000	0.0000	0.0000
0.0156	0.0086	0.0088	0.0099	0.0198	0.0183	0.0206
0.4056	0.3499	0.3910	0.4130	0.4357	0.4303	0.4363
0.0000	0.0000	0.0000	0.0000	0.0000	0.0000	0.0000
0.0000	0.0000	0.0000	0.0000	0.0000	0.0000	0.0000
0.0000	0.0000	0.0000	0.0000	0.0000	0.0000	0.0000
0.0076	0.0078	0.0105	0.0148	0.0000	0.0000	0.0000
0.0146	0.0115	0.0064	0.0070	0.0054	0.0132	0.0049
0.0000	0.0045	0.0072	0.0000	0.0000	0.0000	0.0000
0.0000	0.0029	0.0000	0.0004	0.0142	0.0136	0.0093
0.0000	0.0000	0.0000	0.0000	0.0000	0.0000	0.0000
0.0000	0.0000	0.0000	0.0000	0.0000	0.0000	0.0000
0.0000	0.0000	0.0000	0.0000	0.0000	0.0000	0.0000
0.0000	0.0000	0.0000	0.0000	0.0000	0.0000	0.0000
0.0064	0.0029	0.0029	0.0078	0.0000	0.0000	0.0000
0.0000	0.0000	0.0000	0.0000	0.0086	0.0212	0.0123
0.1856	0.2159	0.2029	0.1823	0.1930	0.1760	0.2116

3G D56						
M1	M2	M3	M4	M5	M6	M7
0.3641	0.3995	0.3480	0.3579	0.3048	0.3328	0.3194
0.0000	0.0000	0.0000	0.0000	0.0000	0.0000	0.0000
0.0000	0.0000	0.0000	0.0000	0.0000	0.0000	0.0000
0.0000	0.0000	0.0000	0.0000	0.0000	0.0000	0.0000
0.0000	0.0000	0.0000	0.0000	0.0000	0.0000	0.0000
0.0000	0.0000	0.0000	0.0000	0.0000	0.0000	0.0000
0.0132	0.0109	0.0123	0.0165	0.0214	0.0167	0.0193
0.4007	0.3464	0.3375	0.3694	0.4256	0.3515	0.4454
0.0000	0.0000	0.0000	0.0000	0.0000	0.0000	0.0000
0.0000	0.0000	0.0000	0.0000	0.0000	0.0000	0.0000
0.0000	0.0000	0.0000	0.0000	0.0000	0.0000	0.0000
0.0109	0.0097	0.0072	0.0095	0.0000	0.0000	0.0000
0.0082	0.0082	0.0105	0.0132	0.0126	0.0123	0.0076
0.0000	0.0000	0.0000	0.0051	0.0000	0.0000	0.0000
0.0000	0.0000	0.0021	0.0027	0.0054	0.0014	0.0152
0.0000	0.0000	0.0000	0.0000	0.0000	0.0000	0.0000
0.0000	0.0000	0.0000	0.0000	0.0000	0.0000	0.0000
0.0000	0.0000	0.0000	0.0000	0.0000	0.0000	0.0000
0.0000	0.0000	0.0000	0.0000	0.0000	0.0000	0.0000
0.0012	0.0035	0.0043	0.0023	0.0000	0.0000	0.0000
0.0000	0.0000	0.0000	0.0000	0.0130	0.0235	0.0107
0.1776	0.1926	0.2451	0.2025	0.2013	0.2441	0.1692

3G D63						
M1	M2	M3	M4	M5	M6	M7
0.3394	0.3707	0.3414	0.3986	0.3490	0.3087	0.3392
0.0000	0.0000	0.0000	0.0000	0.0000	0.0000	0.0000
0.0000	0.0000	0.0000	0.0000	0.0000	0.0000	0.0000
0.0000	0.0000	0.0000	0.0000	0.0000	0.0000	0.0000
0.0000	0.0000	0.0000	0.0000	0.0000	0.0000	0.0000
0.0000	0.0000	0.0000	0.0000	0.0000	0.0000	0.0000
0.0117	0.0119	0.0136	0.0088	0.0146	0.0191	0.0216
0.3789	0.3647	0.4419	0.3834	0.4087	0.4507	0.4460
0.0000	0.0000	0.0000	0.0000	0.0000	0.0000	0.0000
0.0000	0.0000	0.0000	0.0000	0.0000	0.0000	0.0000
0.0000	0.0000	0.0000	0.0000	0.0000	0.0000	0.0000
0.0161	0.0148	0.0156	0.0138	0.0000	0.0000	0.0000
0.0128	0.0144	0.0099	0.0000	0.0078	0.0000	0.0047
0.0045	0.0074	0.0078	0.0047	0.0000	0.0000	0.0000
0.0021	0.0043	0.0016	0.0043	0.0023	0.0037	0.0091
0.0000	0.0000	0.0000	0.0000	0.0000	0.0000	0.0000
0.0000	0.0000	0.0000	0.0000	0.0000	0.0000	0.0000
0.0000	0.0000	0.0000	0.0000	0.0000	0.0000	0.0000
0.0000	0.0000	0.0000	0.0000	0.0000	0.0000	0.0000
0.0043	0.0000	0.0027	0.0019	0.0000	0.0000	0.0000
0.0000	0.0000	0.0000	0.0000	0.0243	0.0161	0.0171
0.1970	0.1807	0.1395	0.1632	0.1780	0.1860	0.1492

3G D70						
M1	M2	M3	M4	M5	M6	M7
0.3593	0.3587	0.3534	0.3805	0.2937	0.3412	0.2861
0.0000	0.0000	0.0006	0.0000	0.0000	0.0000	0.0000
0.0000	0.0000	0.0000	0.0000	0.0000	0.0000	0.0000
0.0000	0.0000	0.0000	0.0000	0.0000	0.0000	0.0000
0.0000	0.0000	0.0000	0.0000	0.0000	0.0000	0.0000
0.0000	0.0000	0.0000	0.0000	0.0000	0.0000	0.0000
0.0128	0.0119	0.0130	0.0113	0.0185	0.0154	0.0233
0.3651	0.3727	0.3536	0.3295	0.4394	0.4003	0.4655
0.0000	0.0000	0.0000	0.0000	0.0000	0.0000	0.0000
0.0000	0.0000	0.0000	0.0000	0.0000	0.0000	0.0000
0.0000	0.0000	0.0000	0.0000	0.0000	0.0000	0.0000
0.0088	0.0132	0.0095	0.0080	0.0000	0.0000	0.0000
0.0095	0.0097	0.0088	0.0095	0.0070	0.0076	0.0047
0.0066	0.0000	0.0000	0.0039	0.0000	0.0000	0.0000
0.0033	0.0019	0.0010	0.0000	0.0142	0.0072	0.0043
0.0000	0.0000	0.0000	0.0000	0.0000	0.0000	0.0000
0.0000	0.0000	0.0000	0.0000	0.0000	0.0000	0.0000
0.0000	0.0000	0.0000	0.0000	0.0000	0.0000	0.0000
0.0000	0.0000	0.0000	0.0000	0.0000	0.0000	0.0000
0.0014	0.0000	0.0000	0.0043	0.0000	0.0000	0.0000
0.0000	0.0000	0.0000	0.0000	0.0198	0.0206	0.0152
0.2122	0.2112	0.1988	0.2241	0.1904	0.1860	0.1809

TRANSPARENT METHODS

Preparation of donor caeca material for transplantation

For the native microbiota or antibiotic-disrupted microbiota group, donor material were derived from the caeca of three 20-week old female C57BL/6J non-littermate mice that were separately housed. The mice were bred and maintained at 22°C ± 2°C, under a 12-hr light-dark cycle, at the SAHMRI Bioresources animal facility, Adelaide, Australia. To induce antibiotic disruption of gut microbiota, donor mice of the antibiotic-disrupted microbiota received water containing erythromycin ethylsuccinate (20 mg/kg) for 90 days to achieve stable gut dysbiosis, as opposed to the plain water received by donor mice of the native microbiota. The antibiotic erythromycin was used as it resulted in disruption of the microbial community, but does not alter bacterial load levels in comparison to the native microbiota. Donor mice were maintained on a Teklad Global 18% Protein Rodent Diet (Envigo, Huntingdon, UK) throughout the study. Mice were killed by carbon dioxide inhalation. Caeca were harvested immediately and transferred to 15% anaerobic glycerol-phosphate buffered saline (PBS).

The subsequent processing was performed under anaerobic conditions (10% CO_2, 10% H_2, 80% N_2). Cecal contents were removed from encasing tissue, weighed, resuspended in 4x (w/v) anaerobic PBS, and homogenised by vortexing. The resulting suspension was passed through a Falcon 100 μM nylon cell filter (Thermofisher Scientific, Waltham, USA) to obtain the non-fibrous content. The supernatant from all three caeca was pooled, mixed with an equal volume of 30% anaerobic glycerol-PBS, and stored in a Hungate tube at -80°C until required. Prior to oral gavage, pooled cecal supernatant was diluted with 2x volume of anaerobic PBS (pH 7.2) and sealed in a glass vial within the anaerobic chamber.

Transplantation of donor caeca material into recipient mice

Six to seven week old germ-free female C57BL/6J mice were obtained from the Translational Research Institute (University of Queensland) and housed within germ-free isolators (Park Bioservices LLC, USA) at the SAHMRI Preclinical Imaging and Research Laboratories (PIRL) germ-free facility (Gilles Plains, Adelaide, Australia). Each germ-free mice group (n=7 per group) consisted of three cages, with each cage containing either two to three litter-matched mice. All germ-free mice maintained on autoclaved Teklad Global 18% Protein Rodent Diet (Envigo, Huntingdon, UK), and housed in an IsoCage P Bioexclusion system (Techniplast, Italy) at 22°C ± 2°C, under a 12-hr light-dark cycle throughout the study.

After a 10 day period of acclimatisation, cecal suspensions were instilled into germ-free mice under sterile conditions. Germ-free mice were inoculated with 150 μL of the appropriate donor cecal suspension (containing approximately 10^5-10^6 bacterial cells for both the native and antibiotic-dirupted microbiota) via an oral gavage. Fecal sampling was performed at baseline and throughout the experiment (Figure S1). Animal studies were performed in accordance to comply with the relevant guidelines (Australian Code for the care and use of animals for scientific purposes (8th edition 2013)) and approved by the South Australian Health And Medical Research Institute Animal Ethics Committee under the study reference SAM378.

Fecal pellet collection, DNA extraction, and bioinformatic processing

Mice were placed in sterile individual cages for fecal pellet collection. Fresh fecal pellets were transferred aseptically to 1.5 mL Eppendorf tubes and stored at -80°C prior to analysis. Fecal pellets were resuspended in 300 μL of PBS by vortexing, and pelleted by centrifugation at 13 000 × *g* for 10 min at 4°C. Supernatant was transferred to a sterile 2 mL screwcap tube and stored at -80°C. Pellets underwent DNA extraction by a combination of mechanical and chemical cell lysis methods using the DNeasy PowerSoil kit (QIAGEN, Hilden, Germany), according to the manufacturer's instructions and eluted in 100 μL of sterile DNase- and RNase-free water.

Amplicon libraries of the V4 hypervariable region for 16S rRNA gene amplicon sequencing were prepared from DNA extracts using modified universal bacterial primer pairs 515F and 806R (Choo et al., 2015). Amplicon libraries were indexed, cleaned, and sequenced according to the Illumina MiSeq 16S Metagenomic Sequencing Library Preparation protocol on a 2 x 300 bp Miseq reagent kit v3 at the David R Gunn Genomics Facility, South Australian Health and Medical Research Institute.

Paired-end 16S rRNA gene sequence reads were analysed using QIIME v2.0 (Bolyen et al., 2019). Briefly, de-noising was performed on de-multiplexed sequences using Dada2, with sequence reads truncated at a specific length based on a quality filtering score of 30 to remove low quality sequence region. Taxonomic classification of sequence variants were performed based on a pre-trained classifier composing of the V4 hypervariable region sequences of the SILVA 132 16S rRNA reference database clustered at 97% similarity (Quast et al., 2013). All samples were subsampled to 4,859 reads. Sufficient

coverage at this depth is confirmed by the rarefaction curve, which reached an asymptote. Taxon relative abundance were used in downstream analyses, genus-level relative abundance for the native and antibiotic-disrupted microbiota groups are available in Table S1 and Table S2, respectively. Sequence data is available from the Sequence Read Archive (SRA) repository under the accession number PRJNA592263.

Quantitative PCR
Previously described quantitative PCR (qPCR) assays were used to determine the abundance of *Akkermansia muciniphila* (Wang et al., 2011), *Bifidobacterium* spp (Rinttila et al., 2004) and *Blautia* spp. (Suchodolski et al., 2012), as well as the 16S rRNA gene for total bacterial load (Nadkarni et al., 2002). SYBR-based qPCR assays were performed using 200 nM of each primer, 1X PowerUP SYBR Green mastermix (Thermofisher Scientific, Waltham, USA), 1 µL of DNA, and sterile DNase- and RNase-free water to make up to a total reaction volume of 35 µL. Each reaction was divided to three, 10 µL, replicate reactions. qPCR cycling conditions were 50°C for 2 mins, 95°C for 10 mins, followed by 40 cycles of [95°C for 15 secs, 60°C for 1 min], and a melt curve of 95°C for 15 secs, 60°C for 1 min and 95°C for 15 secs. Total bacterial load was calculated using a standard curve generated from a known concentration of *Escherichia coli* DNA.

Measurement of fecal pH
Fecal samples from each mice were collected and pooled according to cage to a total of approximately 50 – 100mg. Faeces were resuspended in 9x volume of deionized water and the suspension vortexed for 1 min. The pH value of fecal suspension was them measured on a FE20 FiveEasy™ pH meter (Mettler-Toledo AG, Schwerzenbach, Switzerland).

Statistical analyses
Bacterial taxa that were not detected in donor material, or present as a single read in only one sample, were removed from recipient microbiota data. Representation of donor taxa in recipient mice was determined based on their presence in more than one mouse per group. Beta diversity analysis were performed based on weighted UniFrac distances computed between samples using QIIME. Compositional differences between groups and distance to the group centroid were determined based on the PERMANOVA with cage as a nested factor and PERMDISP test, respectively, using PRIMER (Anderson et al., 2008). Comparison between single and multiple gavage groups was performed by Mann-Whitney test using Graphpad PRISM (v8), and taxa relative abundances were compared using a linear mixed-effects model with the *lmerTest* package in R (v3.1-1).

SUPPLEMENTAL REFERENCES

Anderson, M.J., Gorley, R.N. and Clarke K.R. (2008). PERMANOVA+ for primer: Guide to software and statistical methods. In PRIMER-E (PRIMER-E Ltd Plymouth UK), pp. 15-104.

Bolyen, E., Rideout, J.R., Dillon, M.R., Bokulich, N.A., Abnet, C.C., Al-Ghalith, G.A., Alexander, H., Alm, E.J., Arumugam, M., Asnicar, F., et al. (2019). Reproducible, interactive, scalable and extensible microbiome data science using QIIME 2. Nat Biotechnol *37*, 852-857.

Choo, J.M., Leong, L.E.and Rogers, G.B. (2015). Sample storage conditions significantly influence faecal microbiome profiles. Sci Rep *5*, 16350.

Nadkarni, M.A., Martin, F.E., Jacques, N.A.and Hunter, N. (2002). Determination of bacterial load by real-time PCR using a broad-range (universal) probe and primers set. Microbiology *148*, 257-266.

Quast, C., Pruesse, E., Yilmaz, P., Gerken, J., Schweer, T., Yarza, P., Peplies, J.and Glockner, F.O. (2013). The SILVA ribosomal RNA gene database project: improved data processing and web-based tools. Nucleic Acids Res *41*, D590-596.

Rinttila, T., Kassinen, A., Malinen, E., Krogius, L.and Palva, A. (2004). Development of an extensive set of 16S rDNA-targeted primers for quantification of pathogenic and indigenous bacteria in faecal samples by real-time PCR. J Appl Microbiol *97*, 1166-1177.

Suchodolski, J.S., Markel, M.E., Garcia-Mazcorro, J.F., Unterer, S., Heilmann, R.M., Dowd, S.E., Kachroo, P., Ivanov, I., Minamoto, Y., Dillman, E.M., et al. (2012). The fecal microbiome in dogs with acute diarrhea and idiopathic inflammatory bowel disease. PLoS One *7*, e51907.

Wang, L., Christophersen, C.T., Sorich, M.J., Gerber, J.P., Angley, M.T.and Conlon, M.A. (2011). Low relative abundances of the mucolytic bacterium Akkermansia muciniphila and Bifidobacterium spp. in feces of children with autism. Appl Environ Microbiol *77*, 6718-6721.
